
JOURNAL INFORMATION
==============================
NLM Title Abbreviation: Infection
Journal ID: Infection
NLM Journal ID: 0365307

Infection

ISSN: 0300-8126
EISSN: 1439-0973

ARTICLE INFORMATION
==============================
PMCID: PMC7962627
PMID: 33725314
DOI: 10.1007/s15010-021-01588-8
Article ID: 1588
Article version: 1
Subjects: Abstracts

Abstracts of the 10th German/Austrian AIDS Conference

Electronic publication date: 2021 Mar 16
Print publication date: 2021
Volume: 49
Issue: Suppl 1
First page: 1
Last page: 59
Copyright: © The Author(s) 2021
License: Open AccessThis article is licensed under a Creative Commons Attribution 4.0 International License, which permits use, sharing, adaptation, distribution and reproduction in any medium or format, as long as you give appropriate credit to the original author(s) and the source, provide a link to the Creative Commons licence, and indicate if changes were made. The images or other third party material in this article are included in the article's Creative Commons licence, unless indicated otherwise in a credit line to the material. If material is not included in the article's Creative Commons licence and your intended use is not permitted by statutory regulation or exceeds the permitted use, you will need to obtain permission directly from the copyright holder. To view a copy of this licence, visit http://creativecommons.org/licenses/by/4.0/.
License URL: https://creativecommons.org/licenses/by/4.0/

issue-copyright-statement: © Springer-Verlag GmbH Germany, part of Springer Nature 2021

==============================

Infection (2021) 49 (Suppl 1):S1–S59
https://doi.org/10.1007/s15010-021-01588-8

© The Author(s) 2021

DÖAK 2021

DEUTSCH-
ÖSTERREICHISCHER
AIDS-KONGRESS

25th to 27th March 2021

### Abstracts of the 10th German/Austrian AIDS Conference

Dear colleagues,

Experts from Germany, Austria and other countries will meet online for the 10th German-Austrian AIDS conference from March 25–27, 2021. Originally planned to be held in Munich for the first time since 1996, the ongoing coronavirus pandemic forced us to host this as an entirely virtual conference.

We are glad to share with you that even during these difficult times, we have received a large number of high-quality abstract submissions. After a stringent peer review process the majority of abstracts was accepted either as oral or poster presentation.

We feel that this scientific conference with almost 200 presentations from academia, community and industry will provide an interactive forum for a highly valuable scientific exchange about current developments in HIV/AIDS care. Looking forward to seeing you all.

Christian Hoffmann, Oliver T. Keppler and Christoph Spinner

### Contents

### Abstracts

Virology, immunology. . . . . . . . . . . . . . . . . . . . . . . . . . . . . . . . . S4

Abstracts 001, 002, 003, 004, 005, 006

Antiretroviral therapy, new agents, long acting. . . . . . . . . . . . . . . . S6

Abstracts 007, 008, 009, 010, 011

Antiretroviral therapy: strategies. . . . . . . . . . . . . . . . . . . . . . . . . . . S8

Abstracts 012, 013, 014, 015, 016, 017, 018, 019, 020, 021, 022, 023, 024, 025, 026, 027, 028

Antiretroviral therapy: resistance . . . . . . . . . . . . . . . . . . . . . . . . S17

Abstracts 029, 030, 031, 032, 033

Antiretroviral therapy: costs and safety . . . . . . . . . . . . . . . . . . . . S20

Abstracts 034, 035, 036, 037, 038, 039, 040, 041, 042

Oncology. . . . . . . . . . . . . . . . . . . . . . . . . . . . . . . . . . . . . . . . . S24

Abstracts 043, 044, 045

AIDS, neurology and other clinical issues . . . . . . . . . . . . . . . . . . S26

Abstracts 046, 047, 048, 049, 050, 051, 052, 053, 054, 055, 056

HIV and COVID-19 . . . . . . . . . . . . . . . . . . . . . . . . . . . . . . . . . . S30

Abstracts 057, 058, 059, 060, 061, 062, 063, 064, 065, 066, 067, 068, 069, 070

Sexually transmitted diseases . . . . . . . . . . . . . . . . . . . . . . . . . . . S36

Abstracts 071, 072, 073, 074, 075, 076, 077, 078, 079, 080, 081, 082

Pediatrics, pregnancy. . . . . . . . . . . . . . . . . . . . . . . . . . . . . . . . S41

Abstracts 083, 084, 085, 086, 087, 088

Pre-exposure prophylaxis (PrEP). . . . . . . . . . . . . . . . . . . . . . . . . S43

Abstracts 089, 090, 091, 092, 093, 094, 095, 096, 097, 098, 099

Prevention other than PrEP . . . . . . . . . . . . . . . . . . . . . . . . . . . . S48

Abstracts 100, 101, 102

Chemsex, drug use . . . . . . . . . . . . . . . . . . . . . . . . . . . . . . . . . . S49

Abstracts 103, 104, 105, 106, 107, 108, 109

Social aspects, stigmatization, barriers. . . . . . . . . . . . . . . . . . . . . S52

Abstracts 110, 111, 112, 113, 114, 115, 116, 117

### Virology, immunology

### 001
### Paper-ID: 47270, O1

### Distinct immunoglobulin Fc-glycosylation patterns are associated with disease non-progression and broadly neutralising antibody responses in HIV-infected children

Maximilian Muenchhoff[1], Julia Roider[2], Amy Chung[3], Alter Galit[4], Philip Goulder[5]
*[1]Max von Pettenkofer Institute, LMU; [2]LMU München; [3]University of Melbourne; [4]Ragon-Institute; [5]Oxford University*

**Introduction:** A prophylactic HIV vaccine would ideally induce protective immunity during childhood, prior to sexual debut. Children develop potent broadly neutralising antibody (bnAb) responses faster and at higher frequencies than adults, but little is known about the underlying mechanisms, or the potential role of Fc-mediated effector functions in disease progression.
**Methods:** We therefore performed systems immunology, with immunoglobulin profiling, on treatment-naïve vertically HIV-infected children with progressive and non-progressive disease.
**Results:** Pediatric non-progressors (PNPs) showed distinct immunoglobulin profiles, characterized by higher IgG levels against p24 and increased ability to elicit potent gp120- and p24-specific Fc-mediated NK-cell effector functions, suggesting a protective role for these non-neutralizing antibodies in vivo. Consistent with higher levels of immune activation, Fcglycan structures in progressor children were characterized by inflammatory signatures, with reduced levels of galactosylation compared to HIV-uninfected children, whereas PNPs showed lower levels of Fc fucosylation. However, and in contrast to previous reports in adults, both groups of children showed higher levels of gp120-specific IgG Fc-glycan sialylation compared to bulk IgG. Importantly, higher levels of Fc-glycan sialylation were associated with increased bnAb breadth, providing the first evidence that Fc-sialylation may play an important role in driving affinity maturation of HIV specific antibodies in children, a mechanism that could be exploited for vaccination strategies against HIV acquisition.

### 002
### Paper-ID: 47270, O2

### Fc-Gamma-receptor-mediated trogocytosis of HIV co-receptor CCR5 facilitates HIV infection

Hong-Ru Chen, Manuel Albanese, Maximilian Münchhoff, Madeleine Gapp, Katharina Hofmann, Oliver Keppler
*Max von Pettenkofer Institute, LMU*

**Introduction:** HIV-1 infection of cells that do not express CD4 and/or CCR5 has been reported in various tissues in vivo. CCR5-negative resting CD4T-cells particularly contribute to the latent reservoir of HIV that can harbor transcriptionally silent proviruses, resulting in the obstacle of curing. In this study, we demonstrated how FcγRs mediate the transfer of CCR5 onto CD4T-cells through a process called trogocytosis, resulting in higher infection level of HIV-1.
**Methods:** In the in vitro setting, we co-cultured donor HEK293T cells expressing FcγRs and coreceptor CCR5 with the target SupT1, followed by assessing transfer levels of FcγRs and CCR5 with imaging and flow cytometry. Sera from patients of HIV-1 and other diseases were added in the culture, and the IgGs in the sera were further purified and analysed. The infection/fusion levels of CD4T-cells were assessed with R5- and X4-tropic HIV-1 after co-cultured primary CD4T-cells and M2 macrophages.
**Results:** Among all the FcγRs, CD32B was highly expressed on macrophages, and was able to trigger the most efficient transfer of CCR5 onto interacting target cells. Imaging, flow cytometric approaches and functional assays suggested that functional receptors with correct topology were transferred in membrane patches into the target cells' plasma membrane. Moreover, IgGs binding to T cells were able to boost trogocytosis by bridging between FcγRs and the target cells, and these IgGs were mainly found in the sera of HIV-1 patients. Most importantly, the receptor-complex-specific entry and infection of HIV-1 were drastically enhanced in resting CD4T-cells which had been co-cultured with M2 macrophages that express high levels of FcγRs.
**Conclusions:** We suggest a model in which this actively regulated transfer of bioactive receptors between closely interacting immune cells can transiently modify their functionality, which provides HIV-1 an expanded susceptibility on otherwise non-permissive cells that may contribute to the latent HIV reservoir in vivo.

### 003
### Paper-ID: 47291, O3

### Complement potentiates immune sensing of HIV-1 and early type I IFN responses

Doris Wilflingseder[1], Wilfried Posch[1], Marta Bermejo-Jambrina[2], Teunis Geijtenbeek[2]
*[1]Institut für Hygiene und Medizinische Mikrobiologie, Medizinische Universität Innsbruck; [2]Academic Medical Center, Amsterdam*

**Introduction:** Complement-opsonized HIV-1 triggers efficient antiviral type I interferon (IFN) responses in dendritic cells (DCs), which play an important role in protective responses at the earliest stages in retroviral infection. In contrast non-opsonized HIV-1 does not induce type I IFN responses as HIV-1 suppresses or escapes sensing by STING and MAVS associated sensors. It is unclear how complement opsonization of HIV-1 leads to type I IFN responses.
**Results:** Here we have identified a novel sensing pathway, where attachment of complementopsonized HIV-1 via complement receptors activates DCs in a CCR5/RIG-I/MAVS/TBK1-dependent fashion. Increased fusion of complement-opsonized HIV-1 via complement receptor 4 and CCR5 leads to increased incoming HIV-1 RNA in the cytoplasm which is sensed by RIG-I and to a lesser extent by MDA-5. Moreover, complement-opsonized HIV-1 down-modulated the MAVS suppressive Raf-1/PLK1 pathway, thereby opening the antiviral recognition pathway via MAVS. This in turn was followed by MAVS aggregation and subsequent TBK1/IRF3/NFκB activation in DCs exposed to complementcoated but not non-opsonized HIV-1. Thereby DCs were activated to produce increased type I IFN levels and improved HIV-1-specific adaptive immunity.
**Conclusions:** Our data strongly suggest that complement is important in the induction of efficient antiviral immune responses by preventing HIV-1 suppressive mechanisms as well as inducing specific cytosolic sensors.

**004**
**Paper-ID: 47328, O4**

### Differential susceptibility of CD4+ T-cell subsets to transcriptional modulation by HIV-1 latency-reversing agents assessed by single cell RNA sequencing

Julia Kazmierski[1], Dylan Postmus[1], Emanuel Wyler[2], Cornelius Fischer[3], Jenny Jansen[1], Karolin Meixenberger[4], Sarah Nathalie Vitcetz[3], Madlen Sohn[3], Sascha Sauer[3], Norbert Bannert[4], Markus Landthaler[2], Christine Goffinet[1]
*[1]Institute of Virology-Charité-Universitätsmedizin Berlin; [2]Berlin Institute for Medical Systems Biology-MaxDelbrück-Center for Molecular Medicine in the Helmholtz Association; [3]Scientific Genomics Platforms, Laboratory of Functional Genomics, Nutrigenomics and Systems Biology-Max-Delbrück-Center for Molecular Medicine; [4]Division of HIV and Other Retroviruses-Robert Koch Institute.*

**Introduction:** "Shock-and-kill" is one of the conceptually most advanced strategies towards establishment of an HIV-1 cure. Treatment with latency-reversing agents (LRAs), including histone deacetylase inhibitors with chromatin-remodelling capabilities, combined with anti-retroviral therapy, reactivates HIV-1 transcription in vivo. However, LRA treatment fails to significantly reduce the HIV-1 reservoir in HIV1-positive individuals, indicating that it is insufficient to eliminate latently infected cells. The global and T-cell-specific impact of individual LRAs on the transcriptome of CD4+ T-cells, the main HIV-1 reservoir containing cell type in vivo, remains understudied.
**Methods:** Here, using single cell RNA-sequencing and flow cytometry, we characterize LRA-induced alterations of the CD4+ T-cell subset composition of subpopulation-specific transcriptomes, and HIV-1 transcripts using Vorinostat and Panobinostat as two prototypic HDAC inhibitors.
**Results:** Ex vivo exposure of CD4+ T-cells from aviremic HIV-1-positive individuals to Panobinostat markedly reduced the percentage of TREG and TEM cells. Furthermore, Panobinostat altered expression of a multitude of interferon-regulated genes, resulting in suppression of several well-characterized antiviral genes. Exposure to Vorinostat resulted in a comparably mild change of cellular transcriptomic profile and T-cell subset composition. Nevertheless, selected interferon-regulated genes exhibited a subset-specific expression profile upon treatment. Finally, in both Panobinostat or Vorinostat-treated CD4+ T-cells from aviremic HIV-1-positive individuals, reads mapping to the HIV-1 genome were detected in a total of 50 cells, suggesting that HIV-1 reactivation may be detectable at the single cell level in CD4+ T-cells from HIV-1-positive individuals.
**Conclusion:** We conclude that ex vivo treatment with two individual HDAC inhibitors induces an overall proviral milieu in CD4+ T-cell subsets. While this proviral state might be favorable for efficient HIV-1 reactivation, we hypothesize that it may impede the instruction of activation of cellular and adaptive immunity required for effective killing of reactivated cells. Furthermore, current analysis focuses on identification of specific properties of single cells undergoing HIV-1 reactivation.

**005**
**Paper-ID: 47493, O5**

### LFA-1 and ICAM-1 are critical factors for fusion and cell-to-cell transmission of murine leukemia virus

Rebecca Engels, Lisa Falk, Manuel Albanese, Oliver Keppler, Xaver Sewald
*Max von Pettenkofer Institute, LMU*

**Introduction:** Retroviruses like human immunodeficiency virus (HIV) and the mouse pathogenic murine leukemia virus (MLV) can spread between leukocytes in a cell contact-dependent manner. Productively infected cells can transfer virus particles to neighboring lymphocytes across stable cell–cell contacts (cis-infection). In addition, cells that are not susceptible to retroviral infection can contribute to the efficient spread of retroviruses by a mechanism called trans-infection. Macrophages and dendritic cells expressing the lectin CD169 support trans-infection of lymphocytes by MLV and HIV in vitro and in vivo. Recently, intravital imaging of MLV infection in peripheral lymph nodes revealed stable cellcell contacts during trans- and cis-infection, indicating a role of cell adhesion in retrovirus spread in vivo. Here, we study the function of the cell adhesion proteins LFA-1 (CD11a/CD18 heterodimer) and ICAM-1 for retrovirus fusion and spread by trans- and cis-infection in vitro and in vivo.
**Results:** Using cell marker analysis following MLV infection, we identify central memory CD4+ T cells and FoxP3+ CD4+ T cells as target cells in peripheral lymph nodes. Interestingly, infection of CD11a- and ICAM-1-knockout (KO) mice results in reduced spreading of MLV within the susceptible cell populations compared to wild-type mice. In vitro co-culture assays for trans- and cis-infection in the presence of blocking antibodies and assays with primary leukocytes isolated from CD11a- and ICAM1-KO mice confirm a crucial role of LFA-1 and ICAM-1 in retrovirus spread at the cellular level.
Interestingly, both pathways critically dependent on the expression of ICAM-1 on donor cells and LFA-1 on target cells. These results are verified in vivo by adoptive transfer experiments. We further reveal a role of ICAM-1 and LFA-1 in virion fusion in vitro and in vivo by adapting the BlaM-based virion fusion assay.
**Conclusions:** This study provides novel mechanistic details about retroviral dissemination between tissue-specific lymphocyte subpopulations in vitro and in vivo.

**006**
**Paper-ID: 46356, O6**

### Follicular CD8 T cells in the peripheral blood of acutely HIV infected humans

Susanne Rüger[1], Kathrin Held[2], Lisa Rogers[2], Renate Stirner[1], Nils Postel[3], Elke Gersbacher[4], Joanna Eger[5], Johannes Bogner[1], Rika Draenert[6], Christof Geldmacher[2], Julia Roider[1]
*[1]Ludwig-Maximilians-University Munich, Internal Medicine Hospital and Clinic IV, University Hospital Munich; [2]Division of Infectious Diseases and Tropical Medicine, University Hospital, LMU Munich; [3]Prinzmed; [4]MUC Research, Clinical Research; [5]Zentrum für Innere Medizin und Infektiologie; [6]Ludwig-Maximilians University Munich, Antibiotic Stewardship Team*

**Introduction:** Most of the HIV-1 viral reservoir is located within lymphatic tissue and hence for an effective treatment/ cure strategy this niche has to be approached. CD8 T cells that express the follicular homing receptor CXCR5 have been identified both in lymphoid tissue as well as in circulation. These cells express a mostly memory-like phenotype and exert potentially more helper and regulatory than cytotoxic effector functions. We analysed the conditions under which follicular homing properties of circulating CTLs were induced in acute HIV infection and if HIV-specific follicular CD8 T cells developed in immediately treated subjects. The results were compared to those of chronically infected individuals.
**Methods:** PBMC samples from 27 acutely HIV-infected individuals immediately starting ART were analysed by flow cytometry at baseline, week 12 and 48 and compared to controls (table1). For functional characterization of HIV-specific CXCR5 + CD8 T cells, PBMCs were stimulated overnight with HIV peptides and assessed by intracellular cytokine staining assays.
**Results:** CXCR5 expression on CD8 T cells in acutely infected individuals was significantly increased between baseline and week 12. At baseline, chronically infected patients showed a significantly higher proportion of CXCR5 + CD8 T cells than acutely infected individuals. An inverse correlation between viral load and CXCR5 + CD8 T cell proportion was observed in acutely infected patients. The cross sectional analysis revealed that in contrast to mainly terminally differentiated CXCR5-CD8 T cells, CXCR5 + CD8 T cells express preferentially a less differentiated effector memory phenotype. The expression of the exhaustion markers TIM-3 and PD-1 was significantly increased on CXCR5 + CD8 T cells when compared to CXCR5-CD8 T cells. The presented results are preliminary as the analysis of the data is ongoing.
**Conclusion:** The results of this project will inform if and how follicular CD8 T cells might form part of future HIV cure strategies.

| | | n | %male | age[a] (y) | CD4-Count[a] (cells/µl) | VL[a] (copies/µl) |
|---|---|---|---|---|---|---|
| acute.infect. | TopHIVFUTURE[b] | 27 | 96% | 42 [29-47] | 418 [316,5-612,0] | 5.340.000 [1.000.000-10.000.000] |
| acute.infect. | TopHIVPAST[c] | 10 | 100% | 36 [31-45] | 991,5 [666,8-1164] | 0 |
| chron.infect.[b] | | 10 | 90% | 41 [34-45] | 351 [304,5-511,5] | 36.021 [11.562-57.372] |
| uninfected | | 5 | 40% | 48 [41-57] | n.a. | n.a. |
| Table1: Cohorte characteristics | | | | | | |
| [a]reported as median values; IQR shown in brackets; [b]data from the first sample (untreated); [c]data as blood samples drawn | | | | | | |

### Antiretroviral therapy: new agents, long acting

### 007
### Paper-ID: 46971, P1

### GS-6207 sustained delivery formulation supports 6-month dosing interval

Hans-Jürgen Stellbrink[1], Rebecca Begley[2], Justin Lutz[2], Martin Rhee[2], Hadas Dvory-Sobol[2], Anna Chiu[2], Steve K. West[2], Jessica Corpus[2], John Ling[2], Polina German[2]
*[1]ICH Study Center; [2] Gilead Sciences Inc*

**Introduction:** GS-6207, a potent, selective, first-in-class, multi-stage inhibitor of HIV-1 capsid is in development for treatment of HIV. Subcutaneous GS-6207 can be administered quarterly, or less frequently, and has potent antiviral activity in people with HIV. In this study, the safety and single ascending dose (SAD) pharmacokinetics (PK) of a new sustained-delivery SC GS-6207 formulation were evaluated in HIV negative participants.
**Methods:** In this ongoing, randomized, blinded, placebo-controlled SAD Phase 1 study, participants were randomized (4:1) to receive 300 mg/mL SC GS 6207 (n = 8/cohort) or placebo (N = 2/cohort), at 300 (1 × 1.0 mL) or 900 mg (3 × 1.0 mL or 2 × 1.5 mL). PK and safety data were collected through ~ 64 weeks post dose.
**Results:** All 30 participants completed dosing. Interim safety and PK data are available through 40 (300 mg), 28 (900 mg; 3 × 1.0 mL) and 20 weeks (900 mg; 2 × 1.5 mL) post-dose. SC GS-6207 was generally well tolerated. No serious or Grade 3 or 4 AEs related to study drug, or AEs leading to discontinuation occurred. The most common AEs were injection site induration (87%), erythema (70%), or pain (63%); all were mild. There were no clinically relevant ≥ Grade 3 laboratory abnormalities. GS-6207 exposures increased in a dose-proportional manner. Maximal concentrations of GS-6207 were achieved 11 to 14 weeks post-dose (Tmax), and GS-6207 apparent t1/2 was ~ 15 weeks. A slow initial release of GS-6207 was observed, and therapeutic plasma concentrations were sustained for at least 6 months following 900 mg SD. Similar PK following a 900 mg dose administered as either 3 × 1.0 mL or 2 × 1.5 mL SC injections was observed.
**Conclusions:** Preliminary PK and safety data suggest SC GS-6207 300 mg/mL (300 and 900 mg SD) is well-tolerated. 900 mg SD provides therapeutic concentrations for 6 months post-dose. These data support use of this formulation as an every 6 month agent in subsequent clinical trials.

### 008
### Paper-ID: 46804, P2

### Viral and participant factors influence virologic outcome to CAB + RPV LA: multivariable and baseline factor analyses across ATLAS, FLAIR, and ATLAS-2M

David A. Margolis[1], Jonathan M. Schapiro[2], Carlo Federico Perno[3], Daniel R. Kuritzkes[4], Romina Quercia[1], Parul Patel[1], Joseph W. Polli[1], Amy Cutrell[1], David Dorey[5], Yongwei Wang[1], Sterling Wu[5], Veerle van Eygen[6], Herta Crauwels[6], Susan Ford[5], Mark Baker[1], Christine Talarico[1], Marty St. Clair[1], Jerry Jeffrey[1], C. Thomas White[1], Simon Vanveggel[6], Rodica Van Solingen-Ristea[6], Kati Vandermeulen[6], William Spreen[1], Jan van Lunzen[1], Jenny Scherzer[1]
*[1]ViiV Healthcare; [2]National Hemophilia Center, Sheba Medical Center; [3]IRCCS Bambino Gesu' Pediatric Hospital; [4]Division of Infectious Diseases, Brigham and Women's Hospital; [5]GlaxoSmithKline; [6]Janssen Research and Development*

**Introduction:** Phase III studies demonstrated efficacy and safety of long-acting (LA) cabotegravir (CAB) and rilpivirine (RPV) Q4W in ATLAS and FLAIR, and Q8W in ATLAS-2M, with virologic suppression and confirmed virologic failure (CVF) rates of 94% and ~ 1%, respectively. Post-hoc analyses explored factors associated with virologic outcome at Wk48.
**Methods:** Multivariate analysis pooled data from 1039 HIV-infected adults naive to CAB + RPV to examine the influence of baseline viral/participant factors, dosing regimen, and plasma drug concentrations on CVF using regression modeling and variable selection procedure. Contribution of retained baseline factors alone or in combination to CVF was evaluated.
**Results:** 94.3% (980/1039) of participants on Q4W and Q8W dosing maintained virologic suppression through Wk48, with only 1.25% (13/1039) having CVF. Of 13 participants with CVF, 3 were from the EU, with none from Germany. Four covariates were significantly associated ($P < 0.05$) with increased CVF risk: baseline RPV resistance mutations, A6/A1 HIV-1 subtype, body mass index (BMI; associated with CAB pharmacokinetics), and Wk8 RPV concentration. A6/A1 and L74I highly correlated, but only 1 (1/57 [1.75%]; 95% CI 0.04–9.4) participant with L74I alone had CVF, consistent with the overall population rate. Other variables (e.g, Q4W or Q8W dosing, female at birth, other viral subtypes) had no significant association. Participants with 0 or 1 significant baseline factor had high virologic success rates (94.8% and 96.0%, respectively; Table). Combination of ≥ 2 factors was uncommon (3.37%; 35/1039), with 71.4% (25/35) maintaining HIV-1 suppression.
**Conclusions:** CAB + RPV LA demonstrated high efficacy in phase III studies and noninferiority to oral antiretroviral therapy for maintaining virologic suppression. No baseline factor alone predicted CVF. In 3% of participants with ≥ 2 baseline factors, including RPV resistance mutations, A6/A1 subtype, or higher BMI, CVF risk modestly increased. These findings should be contextualized with the high overall success rate of Q4W and Q8W regimens.

**Table. Week 48 Outcomes by Presence of Key Baseline Factors of RPV RAM, Subtype A6/A1, BMI ≥30 kg/m²**

| Baseline factors (number) | Virologic successes[1] | Confirmed virologic failure (%)[2] |
|---|---|---|
| 0 | 694/732 (94.8) | 3/732 (0.41) |
| 1 | 261/272 (96.0) | 1/272 (0.37)[3] |
| ≥2 | 25/35 (71.4) | 9/35 (25.7)[4] |
| TOTAL | 980/1039 (94.3) | 13/1039 (1.25) |
| (95% Confidence Interval) | (92.74%, 95.65%) | (0.67%, 2.13%) |

[1]Based on the FDA Snapshot algorithm of HIV-1 RNA <50 copies/mL. [2]Defined as two consecutive measurements of HIV-1 RNA >200 copies/mL. [3]Positive Predictive Value (PPV) <1%; Negative Predictive Value (NPV) 98%; sensitivity 8%; specificity 74%. [4]PPV 26%; NPV 99.6%; sensitivity 69%; specificity 97.5%.

### 009
### Paper-ID: 46790, P3

### Safety and efficacy of cabotegravir + rilpivirine long acting with and without oral lead-in: FLAIR week 124 results

Ronald D'Amico[1], Chloe Orkin[2], Enrique Bernal Morell[3], Darrell H. S. Tan[4], Harold Katner[5], Yashna Singh[6], Prof. Hans-Jürgen Stellbrink[7], Elena Belonosova[8], Rebecca DeMoor[9], Sandy Griffith[1], Shanker Thiagarajah[9], Rodica Van Solingen-Ristea[10], Herta Crauwels[10], Susan L. Ford[9], Parul Patel[1], Amy Cutrell[1], Kimberly Y. Smith[1], Kati Vandermeulen[10], David A. Margolis[1], Marty St. Clair[1], William R. Spreen[1], Teresa Finkenzeller[1].

*[1]ViiV Healthcare; [2]Queen Mary University; [3]Hospital General Universitario Reina Sofía; [4]Division of Infectious Diseases, St. Michael's Hospital; [5]Mercer University Medical School; [6]Desmond Tutu HIV Foundation; [7]ICH Study Center; [8]Orel Regional Center for AIDS; [9]GlaxoSmithKline; [10]Janssen Research and Development*

**Introduction:** FLAIR (NCT02938520), a phase III randomized, open-label study, established noninferiority of switching virologically suppressed participants from daily oral dolutegravir/abacavir/lamivudine (CAR) to monthly cabotegravir (CAB) + rilpivirine (RPV) long-acting (LA) following a CAB + RPV oral lead-in (OLI) over 2 y. We report results from the Extension Phase, focusing on efficacy, safety, and tolerability of switching CAR participants to LA therapy with or without OLI.

**Methods:** ART-naive participants achieving virologic suppression (HIV-1 RNA < 50 c/mL) with CAR during the 20-week Induction Phase were randomized (1:1) to continue CAR or switch to LA (283/arm). Participants randomized to LA therapy received an OLI of CAB + RPV once daily for ≥ 4 wk before receiving monthly injectable CAB + RPV LA. At W100, CAR participants could switch to LA therapy (Extension Switch population), either directly (Direct to Inject [DTI] arm) or with a 4-wk OLI (OLI arm), or withdraw. Endpoints assessed at W124 for the Extension Switch population: plasma HIV1 RNA ≥ 50 c/mL and < 50 c/mL, confirmed virologic failure (CVF; 2 consecutive HIV-1 RNA ≥ 200 c/mL), safety, and tolerability.

**Results:** 111 and 121 CAR participants transitioned to CAB + RPV LA, entering the DTI or OLI arms, respectively. At W124, 1 participant (< 1%) in each arm had HIV-1 RNA ≥ 50 c/mL (Table). 99% and 93% of participants in the DTI and OLI arms maintained virologic suppression (HIV-1 RNA < 50 c/mL), respectively. One participant in the DTI arm developed CVF at W112. AEs leading to withdrawal were infrequent. There was one grade 4 drug-related AE in the DTI arm (mixed cellularity Hodgkin's lymphoma). The number of participants experiencing serious AEs was comparable between arms. CAB + RPV LA was well tolerated; injection-site reactions were the most common AE, with most classified as mild/moderate.

**Conclusions:** Switching directly to LA therapy without OLI demonstrated similar efficacy, safety, and tolerability at W124 to treatment with OLI. Suggesting that CAB + RPV LA, with or without OLI, is a well-tolerated, effective maintenance therapy.

**Table. Key Outcomes at Week 124 Data Analysis**

| Outcome, n (%) Extension Switch Population | DTI arm n=111 | OLI arm n=121 |
|---|---|---|
| HIV-1 RNA <50c/mL at W124* | 110 (99.1) | 113 (93.4) |
| HIV-1 RNA ≥50c/mL at W124* | 1 (0.9) | 1 (0.8) |
| Data in window not <50c/mL | 0 | 1 (0.8)† |
| Discontinued due to lack of efficacy | 1 (0.9) | 0 |
| Discontinued due to other reasons while not suppressed | 0 | 0 |
| No virologic data in W124 window | 0 | 7 (5.8) |
| Discontinued study due to AE or death | 0 | 2 (1.7)‡ |
| Discontinued study for other reasons | 0 | 5 (4.1)§ |
| Number of injections | 2314 | 2128 |
| Number of ISR events | 576 | 338 |
| Grade 1 events – mild | 478 | 271 |
| Grade 2 events – moderate | 97 | 62 |
| Grade 3 events – severe | 1 | 5 |
| ISR duration ≤7 days | 516 | 290 |
| Median ISR duration, days | 3 | 3 |
| Withdrawals due to ISRs | 0 | 1 (0.8) |
| Incidence of skin and subcutaneous tissue disorders (dermatologic AEs) | 19 (17.1)‖ | 10 (8.3) |
| Maximum Grade 3 or 4 AEs | 5 (4.5) | 9 (7.4) |
| Maximum Grade 3 or 4 AEs excluding ISRs | 4 (3.6) | 5 (4.1) |
| Maximum drug-related Grade 3 or 4 AEs excluding ISRs | 1 (0.9) | 0 |
| Maximum Grade 3 or 4 emergent chemistry toxicities | 13 (11.7) | 5 (4.1) |
| Serious AEs | 4 (3.6) | 5 (4.1) |
| Mean change (range) from Extension Baseline¶ in ALT, IU/L | −1.1 (−157, 80) | 1.1 (−37, 45) |
| Mean change (range) from Extension Baseline¶ in AST, IU/L | −0.3 (−257, 308) | −0.7 (−124, 84) |
| Mean change (range) from Extension Baseline¶ in bilirubin, μmol/L | 1.5 (−14, 24) | 1.0 (−8, 14) |
| Liver monitoring/stopping events | 0 | 0 |

AE, adverse event; ALT, alanine aminotransferase; AST, aspartate aminotransferase; DTI, Direct to Inject; ISR, injection site reaction; OLI, oral lead-in; W, Week.
*Per FDA Snapshot algorithm.
†Participant had HIV-1 RNA of 57c/mL.
‡Two discontinuations after OLI: 1 injection site pain, 1 increased weight.
§Two discontinuations during OLI: 1 subject relocation, 1 pregnancy. Three discontinuations after OLI: 1 burden of procedures and intolerability of injections, 1 burden of travel, 1 use of prohibited medicine.
‖Includes 1 cyst and 2 herpes zoster, which were not coded to "skin and subcutaneous tissue disorders".
¶W100.

### 010
### Paper-ID: 46797, P4

### Cabotegravir + rilpivirine long-acting as HIV-1 maintenance therapy: ATLAS week 96 results

Susan Swindells[1], Thomas Lutz[2], Lelanie Van Zyl[3], Norma Porteiro[4], Paul Benn[5], Jenny O. Huang[6], Conn M. Harrington[5], Kai Hove[6], Susan L. Ford[6], Christine L. Talarico[5], Vasiliki Chounta[5], Herta Crauwels[7], Rodica Van Solingen-Ristea[7], Simon Vanveggel[7], David A. Margolis[5], Kimberly Y. Smith[5], Kati Vandermeulen[7], William R. Spreen[5], Alexander Baum[8]

*[1]University of Nebraska Medical Center; [2]Infektiologikum; [3]Syzygy Clinical Research Services; [4]Fundación IDEAA; [5]ViiV Healthcare; [6]GlaxoSmithKline; [7]Janssen Research and Development; [8]ViiV Healthcare GmbH*

**Introduction:** Long-acting (LA) injectable therapies have potential to address some challenges of daily oral ART (eg, pill fatigue, drug/food interactions, stigma, suboptimal adherence). ATLAS (NCT02951052) is a phase III, multicenter, open-label study. W48 data demonstrated switching to monthly injectable cabotegravir (CAB) + rilpivirine (RPV) LA was noninferior to continuing 3-drug daily oral ART (CAR) for adults with HIV-1.

**Methods:** Virologically suppressed ART-experienced participants were randomized (1:1) to continue CAR or switch to LA therapy for a 52-wk Maintenance Phase (MP). After completion, participants could withdraw, transition to ATLAS-2M (NCT03299049; investigating CAB + RPV LA Q8W vs CAB + RPV LA Q4W) or enter an Extension Phase (EP). Participants entering the EP at W52 continued LA therapy (LA arm) or switched from CAR to CAB + RPV LA (Switch arm). Endpoints assessed at W96: plasma HIV1 RNA < 50 c/mL and ≥ 50 c/mL, confirmed virologic failure (CVF; 2 consecutive HIV-1 RNA ≥ 200 c/mL), safety, tolerability, and patient-reported outcomes.

**Results:** Most participants completing the MP transitioned to ATLAS-2M (88%, 502/572), leaving 52 in ATLAS for inclusion in the W96 data analysis. Of these, 100% (23/23) and 97% (28/29) in LA and Switch arms maintained virologic suppression at W96 data analysis, respectively (Table). No participants had CVF during the EP. Safety and tolerability data for LA and Switch arm participants were comparable, similar to data reported during the MP. Most common drug-related adverse events were injection-site reactions, which were generally mild/moderate and of short duration (median duration, 3 days). All Switch arm participants responding to the questionnaire at W96 (100%, 27/27) preferred LA to their previous daily oral regimen.

**Conclusions:** CAB + RPV LA maintained virologic suppression in most participants who entered the EP and were present at the W96 data analysis, with no CVFs or new safety signals identified. These longer-term efficacy/safety data as well as patient preference data support the therapeutic potential of CAB + RPV LA.

**Table. Key Outcomes**

| Outcome, n (%) Intention-to-Treat Exposed and Switch Population | LA arm (Day 1 to W52) n=308 | CAR arm (Day 1 to W52) n=308 | LA arm (W52 to W96) n=280* | Switch arm (W52 to W96) n=174† |
|---|---|---|---|---|
| Present at W96 data analysis‡ | N/A | N/A | 23 | 29 |
| HIV-1 RNA ≥50 c/mL at W96 data analysis | N/A | N/A | 0 | 1 |
| HIV-1 RNA <50 c/mL at W96 data analysis | N/A | N/A | 23 | 28 |
| Number of injections | 6978 | N/A | 1363 | 1264 |
| Number of ISR events | 1460 | N/A | 154 | 238 |
| Grade 1 ISR events – mild | 1156 | N/A | 134 | 184 |
| Grade 2 ISR events – moderate | 283 | N/A | 20 | 51 |
| Grade 3 ISR events – severe | 21 | N/A | 0 | 3 |
| ISR duration ≤7 days | 1288 | N/A | 113 | 199 |
| Number of participants discontinuing due to ISRs | 4 (1) | N/A | 0 | 1 (<1) |
| Overall AEs | 294 (95) | 220 (71) | 1§ | 105 (60) |
| Maximum Grade 3 or 4 AEs | 35 (11) | 23 (7) | 2§ | 7 (4) |
| Drug-related AEs | 255 (83) | 8 (3) | 0§ | 79 (45) |
| Maximum Grade 3 or 4 drug-related AEs | 14 (5) | 1 (<1) | 0§ | 4 (2) |
| AEs leading to withdrawal | 14 (5) | 5 (2) | 2§‖ | 1 (<1)¶ |
| Drug-related AEs leading to withdrawal | 10 (3) | 1 (<1) | 1§ | 1 (<1) |
| Serious AEs** | 13 (4) | 14 (5) | 2§ | 2 (1) |

AE, adverse event; ISR, injection site reaction; LA, long-acting; W, week.
*Number of participants at W52 visit.
†All participants from the CAR arm who received ≥1 dose of cabotegravir/rilpivirine during the Extension Phase.
‡W96±4 weeks.
§Number of new participants with AEs during the Extension Phase.
‖Includes acute hepatitis B and fear.
¶Injection site pain.
** No serious AEs were classified as related to cabotegravir/rilpivirine.

### 011
### Paper-ID: 46759, P5

### Factors associated with interest in a long-acting HIV regimen: perspectives of people living with HIV and physicians in western Europe

Babatunde Akinwunmi[1], Daniel Buchenberger[2], Jenny Scherzer[3], Martina Bode[3], Paolo Rizzini[3], Fabio Vecchio[3], Laetitia Roustand[4], Gaelle Nachbaur[4], Laurent Finkielsztejn[3], Vasiliki Chounta[3], Nicolas Van de Velde[3], Nelles Ruppert[3]
*[1]Zatum LLC; [2]Ipsos Insights LLC; [3]ViiV Healthcare; [4]GlaxoSmithKline*

**Introduction:** Current antiretroviral treatments (ARTs) require daily oral dosing—a challenge for some people living with HIV (PLWHIV). We assessed the interest of PLWHIV and physicians in a longacting regimen (LAR).

**Methods:** Two web-based surveys were administered to 120 HIV physicians and 688 ART-treated PLWHIV from France, Germany, Italy, and the United Kingdom from June to August 2019. A balanced description of a hypothetical LAR was provided, including its efficacy, administration, possible side effects, patient-reported outcomes, and affordability. Interest in receiving ("very" or "highly interested") or ("definitely" or "probably") offering the LAR in different situations, with perceived benefits and concerns, were assessed.

**Results:** Of PLWHIV, 66.4% were men, and 60.6% homosexual. Overall, 65.8% (453/688) of PLWHIV were very or highly interested in trying the LAR, especially those aged < 50 years, 69.8% [338/484]) vs those aged ≥ 50 years (56.4% [115/204]) and those with (75.5% [123/163]) vs without (62.9% [330/525]) the perception that HIV limits day-to-day life (all P < 0.05). PLWHIV with unmet needs felt.

LAR would help with strong medical needs (malabsorption and interfering gastrointestinal conditions, > 90%), suboptimal adherence (80.5% [301/374]), confidentiality or privacy concerns (87.6% [458/523]), and emotional wellbeing related to daily tablet requirements (79.0% [240/304]). Of physicians, willingness to offer LAR varied situationally (Figure). The most favored LAR attributes were ease of travel (56.3% [387/688]) for PLWHIV and increased patient contact (54.2% [65/120]) for physicians. Perceived negative attributes were scheduling challenges (37.2% [256/688]) and resource constraints (57.5% [69/120]) for PLWHIV and physicians, respectively. Physicians estimated 25.7% (SD = 23.1%) of their patients would switch; higher estimates were reported by providers who were male, metropolitan-based, and had fewer patients.

**Conclusions:** Physicians and PLWHIV viewed LAR as addressing unmet needs. Alternative routes of treatment, including LAR, may help improve treatment satisfaction, resulting in greater adherence and retention in care.

**Figure. Degree of Physician Willingness to Offer a Hypothetical Long-Acting Regimen Called Regimen Z for Different Medical or Emotional Challenges Among Their HIV+ Patients**

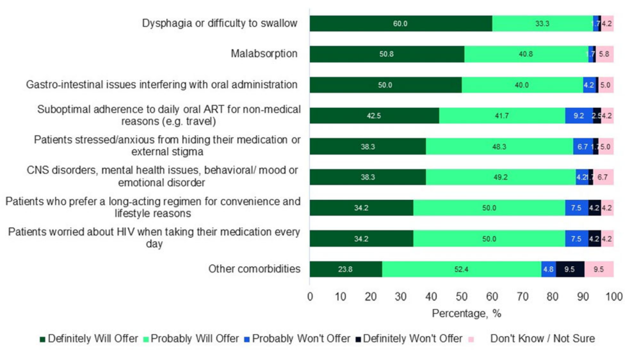

### Antiretroviral therapy: strategies

### 012
### Paper-ID: 46802, O1

### Long-term development of CD4 values in German HIV patients under successful antiretroviral therapy—analysis from the ClinSurv cohort 1999–2018

Uwe Koppe[1], Kirsten Pörtner[1], Christian Kollan[1], Barbara Gunsenheimer-Bartmeyer[1], Matthias an der Heiden[1], Daniel Schmidt[1], Dirk Schürmann[2], Hans-Jürgen Stellbrink[3], Matthias Stoll[4], Jan-Christian Wasmuth[5], Stefan Esser[6], ClinSurv HIV Study Group ClinSurv HIV Study Group[7]

*[1]Robert Koch Institute; [2]Charité–Universitätsmedizin Berlin; [3]Infektionsmedizinisches Centrum Hamburg (ICH); [4]Medical University of Hanover; [5]University Hospital Bonn; [6]University Hospital Essen, University Duisburg-Essen; [7]ClinSurv HIV Study Group*

**Introduction:** Antiretroviral therapy (ART) causes HIV suppression and restoration of immune function in many patients. We investigated the long-term development of CD4 values in HIV patients in Germany under continuous ART.
**Methods:** We included treatment-naïve patients from the ClinSurv-HIV study starting ART between 1999 and 2018, who received uninterrupted ART and did not have virological failure (2 consecutive viral load measurements > 200 copies/µl within 3 months). We analysed the development of CD4 values and the proportion of patients reaching ≥ 650 CD4 cells/µl or remaining < 350 cells/µl.
**Results:** At baseline, we included 11,217 patients with a median age of 38 years (IQR 31–47). 81.1% were male and the median follow-up time was 3.9 years (IQR 1.6–7.7). The mean CD4-value at baseline was 250 cells/µl (95% CI 246–253) and 3.9% of the participants had ≥ 650 cells/µl. The mean CD4-value rose to 473, 537, 594, 630, 674, and 681 cells/µl after 1, 2, 4, 6, 10, and 15 years of continuous ART (Fig. 1). While the strongest CD4 increase occurred within the first 4 years of treatment, the mean CD4 values continued to rise. The proportion of patients reaching CD4 values ≥ 650 cells/µl was 21.3%, 28.9%, 36.8%, 41.1%, 49.0%, and 52.3% after 1, 2, 4, 6, 10, and 15 years. In contrast, the proportion of patients with CD4 cell counts < 350 cells/µl was 35.8%, 25.3%, 17.6%, 12.9%, 10.0%, and 11.2% after 1, 2, 4, 6, 10, and 15 years.
**Conclusion:** Long-term ART without virological failure lead to reconstitution of CD4 values. CD4 counts continued to increase even within the range generally regarded as normal, and as long as 15 years on virological suppression. However, about 10% of the patients had CD4 values < 350 cells/µl after ≥ 10 years of ART. Determinants of long-term CD4-development should be further investigated.
ClinSurv HIV Study Group: Johannes Bogner Bjoern Jensen.
Gerd Fätkenheuer; Jörg Janne Vehreschild; Olaf Degen; Guido Schäfer; Dirk Schürmann Jürgen Rockstroh; Jan-Christian Wasmuth; Andreas Plettenberg Thore Lorenzen; Heinz-August Horst; Carlos Fritzsche Matthias Stoll; Stefan Esser; Annette Haberl; Christoph Stephan; Hans-Jürgen Stellbrink; Axel Adam; Stephan Fenske; Stefan Hansen; Christian Hoffmann; Michael Sabranski; Carl Knud Schewe Thomas Buhk

Figure 1: Development of CD4 values during ART treatment

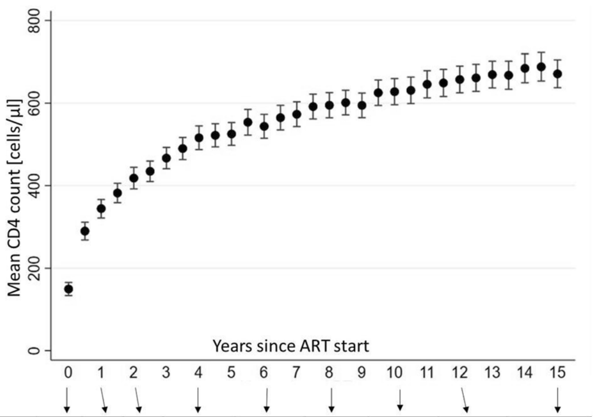

| Patients (n) | 11,217 | 9,433 | 7,812 | 5,529 | 3,954 | 2,693 | 1,703 | 1,034 | 457 |
|---|---|---|---|---|---|---|---|---|---|

**013**
**Paper-ID: 46939, O2**

**CD4/CD8-ratio normalisation by first-line ART-class combination in ClinSurv, a large multicentre HIV-cohort study from Germany 1999–2018**

Kirsten Pörtner[1], Christian Kollan[2], Matthias an der Heiden[2], Barbara Gunsenheimer-Bartmeyer[2], Daniel Schmidt[2], Stefan Esser[3], Andre Fuchs[4], Björn-Erik Ole Jensen[4], Jürgen Rockstroh[5], Guido Schäfer[6], Dirk Schürmann[7], Matthias Stoll[8], Jan-Christian Wasmuth[5], Uwe Koppe[2], On the behalf of the ClinSurv HIV Study Group[9]
*[1]Robert Koch Institute, Postgraduate Training for Applied Epidemiology (PAE), EPIET associated programme; [2]Robert Koch Institute; [3]University Hospital Essen, University Duisburg-Essen; [4]Heinrich Heine University Düsseldorf; [5]University Hospital Bonn; [6]University Medical Centre Hamburg-Eppendorf; [7]Charité -Universitätsmedizin Berlin; [8]Medical University of Hanover; [9]Multiple*

**Introduction:** INSTI-containing antiretroviral treatment (ART) is a recommended first-line therapy for HIV-positive patients. Little is known on the reasons for persistent pathological values of the CD4/CD8-ratio (≤ 1) despite modern ART, leading to increased morbidity and mortality. We investigated CD4/CD8-rationormalisation in patients under first-line therapy in the ClinSurv-HIV cohort to identify factors associated with ratio-normalisation.
**Methods:** We analysed data from 1999 to 2018 from ClinSurv, the largest German multicentre long-term observational HIV-cohort including treatment-naïve, adult patients with a continuous, identical ARTclass-combination containing INSTI, NNRTI or PI. The outcome was the CD4/CD8-ratio-normalisation (defined as > 1 on two consecutive visits within one year). We identified factors associated with rationormalisation during first-line therapy using uni- and multivariable Cox regression.
**Results:** We included 6,736 patients, median (IQR) age at baseline was 38 (31–47) years, 3,548/5,877 (60%) were men who have sex with men (859/6,736 unknown). Median observational time for patients on first-line therapy was 2.5 (0.5–5.5) years. Mean (range) CD4-cell-count at baseline was 278 (0–1770) cells/µl, mean CD4/CD8-ratio 0.3 (0–17). Overall, 22% reached ratio-normalisation within 1.5 (median, IQR 0.5–3.2) years. Heterosexual transmission, age 18–29 years, higher CD4-cell-count at baseline, and viral load ≤ 100,000 copies/ml at baseline as well as an INSTI- or NNRTI-containing firstline regimen were variables univariably associated with ratio-normalisation. After adjusting for transmission route, age, CD4-cell-count, and viral load at baseline, patients treated with

INSTI-regimens normalised their CD4/CD8-ratio more frequently than those under PIregimens (adjusted RR 1.20, 95% CI 1.04–1.39, p = 0.014, table 1).

**Conclusion:** Only a minority of patients reached a normalised CD4/CD8-ratio under first-line therapy within 2.5 (median) years of observation. Patients treated with INSTI-regimens had the highest probability of normalisation compared to NNRTI- and PI-regimens, even after adjusting for transmission route, age, CD4-cell-count and viral load at baseline. INSTI-regimens might be more beneficial as first-line therapy regarding ratio-normalisation.

Table 1. Univariable and multivariable analysis (adjusted for transmission route, age, CD4-cell-count and viral load at baseline) to investigate factors associated with CD4/CD8-ratio-normalisation

| | Univariable analysis | | | Multivariable analysis | | |
|---|---|---|---|---|---|---|
| | RR | p-value | 95% CI | aRR | p-value | 95% CI |
| **Transmission route** | | | | | | |
| Men who have sex with men | 1 | | | 1 | | |
| Intravenous drug use | 0.68 | 0.031 | 0.48-0.96 | 0.87 | 0.440 | 0.61-1.24 |
| Heterosexual | 1.29 | <0.001 | 1.13-1.47 | 1.56 | <0.001 | 1.36-1.78 |
| Origin from high prevalence country | 0.81 | 0.017 | 0.69-0.96 | 1.16 | 0.103 | 0.97-1.38 |
| Other | 2.08 | 0.039 | 1.04-4.18 | 2.02 | 0.048 | 1.01-4.07 |
| Unknown | 0.97 | 0.711 | 0.83-1.14 | 1.14 | 0.105 | 0.97-1.34 |
| | | | | | | |
| **Age [years]** | | | | | | |
| 18-29 | 1.30 | <0.001 | 1.13-1.50 | 1.12 | 0.110 | 0.97-1.29 |
| 30-39 | 1 | | | 1 | | |
| 40-49 | 0.87 | 0.044 | 0.77-1.00 | 0.90 | 0.102 | 0.79-1.02 |
| 50-59 | 0.99 | 0.877 | 0.84-1.16 | 1.01 | 0.856 | 0.86-1.20 |
| 60+ | 0.94 | 0.618 | 0.74-1.20 | 0.93 | 0.586 | 0.73-1.20 |
| | | | | | | |
| **CD4-cell-count at baseline [cells/µl]** | | | | | | |
| ≤200 | 1 | | | 1 | | |
| 201-350 | 2.93 | <0.001 | 2.52-3.42 | 2.96 | <0.001 | 2.53-3.47 |
| 351-500 | 4.58 | <0.001 | 3.90-5.38 | 4.82 | <0.001 | 4.07-5.71 |
| >50 | 8.20 | <0.001 | 6.96-9.66 | 8.63 | <0.001 | 7.23-10.31 |
| | | | | | | |
| **Viral load at baseline [copies/ml]** | | | | | | |
| ≤1000 | 1.00 | 0.95 | 0.86-1.15 | 0.76 | <0.001 | 0.66-0.89 |
| 1001-10,000 | 0.95 | 0.524 | 0.81-1.11 | 0.84 | 0.031 | 0.72-0.98 |
| 10,001-100,000 | 1 | | | 1 | | |
| >100,000 | 0.68 | <0.001 | 0.60-0.77 | 1.01 | 0.865 | 0.89-1.15 |
| | | | | | | |
| **ART-class-combination** | | | | | | |
| INSTI/NRTI | 1.93 | <0.001 | 1.68-2.22 | 1.20 | 0.014 | 1.04-1.39 |
| NNRTI/NRTI | 1.40 | <0.001 | 1.24-1.57 | 1.11 | 0.096 | 0.98-1.25 |
| PI/NRTI | 1 | | | 1 | | |

RR: Rate ratio, aRR: adjusted rate ratio, CI: confidence interval, ART: antiretroviral therapy, INSTI: integrase strand transfer inhibitors, NNRTI: non-nucleoside reverse transcriptase inhibitor, NRTI: nucleoside reverse transcriptase inhibitor, PI: protease inhibitor

ClinSurv HIV Study Group: Johannes Bogner; Bjoern Jensen; Gerd Fätkenheuer; Jörg Janne Vehreschild; Olaf Degen, Guido Schäfer; Dirk Schürmann; Jürgen Rockstroh; Jan-Christian Wasmuth; Andreas Plettenberg; Thore Lorenzen; Heinz-August Horst; Carlos Fritzsche; Matthias Stoll; Stefan Esser; Annette Haberl; Christoph Stephan; Hans-Jürgen Stellbrink; Axel Adam; Stephan Fenske; Stefan Hansen; Christian Hoffmann; Michael Sabranski; Carl Knud Schewe; Thomas Buhk;

### 014
### Paper-ID: 47309, P1

### Two-drug regimen (2DR) for initial HIV treatment?—lessons learned from 20 years RESINA cohort

Nadine Lübke[1], Björn Jensen[2], Michael Böhm[3], Elena Knops[3], Eva Heger[3], Jörg Timm[1], Rolf Kaiser[3]
*[1]Institute of Virology, Heinrich-Heine-University, University Hospital; [2]Department of Gastroenterology, Hepatology and Infectious Diseases, Heinrich-Heine-University, University Hospital; [3]Institute of Virology, University of Cologne*

**Introduction:** Since November 2019 the two-drug regimens (2DR) Dovato® containing lamivudine (3TC) and Dolutegravir (DTG) is recommended by the European guidelines for first-line treatment in HIVinfected patients. Thus, we analysed differences in time to viral suppression in the RESINA cohort and screened for transmitted drug resistance mutations affecting the use of Dovato® in first-line treatment.

**Material:** Overall, 4815 patients from the RESINA cohort, a study initiated in 2000 for the surveillance of transmitted HIV drug resistance, were included in the analysis. Baseline drug resistance was analysed for 3TC and INIs (Tzou et al., 2019).

**Results:** In total, 4591 patients with a majority of men (81.6%) were included. Documented therapies were available in 4219 cases (87.6%) with 3577 corresponding sequence data. Viral loads of HIV before treatment initiation were stable since 2001 with 60% < 100,000, 30% > 100,000 and 10% > 500,000 copies/ml. In contrast, the number of patients with low CD4+ T-cells < 200 cells/µl decreased from 62 to 14%. The time to viral suppression after cART initiation significantly differed between 3DR therapies depending on the drug class, INIs < PIs < NNRTIs (median 10.7w; 16.9w; 19.0w, respectively, p < 0.0001). Independent of the drug class, 2DR and 3DR showed no significant differences in time to viral suppression (13.4 w and 14.6 w, respectively; p = 0.509). The prevalence of baseline drug resistance was rare with 0.4% of the M184V mutation specific for 3TC and 0.7% of transmitted INI mutations.

**Conclusion:** INI-based therapies showed a significant faster time to viral suppression compared to NNRTI- of PIbased therapies, confirming the high antiretroviral potency of INIs. Since only few 2DR therapies were documented, inferiority to 3DR could not be detected. Baseline resistance to 3TC or INIs was only rare detected in our cohort, supporting the potential use of Dovato® for initial HIV treatment.

### 015
### Paper-ID: 47312, P2

### The DoDo experience: an alternative 2DR of doravirine and dolutegravir

Ulrich Seybold, Florian E. Gottwald, Gerardo J. Ibarra Fonseca, Johannes R. Bogner
*LMU Klinikum Innenstadt, Ludwig-Maximilians-Universität München*

**Introduction:** Two-drug regimens (2DR) combining the integrase-inhibitor (INI) dolutegravir (DTG) with either the nucleoside reverse transcriptase-inhibitor (NRTI) lamivudine (3TC) or the non-NRTI (NNRTI) rilpivirine (RPV) are among recommended antiretroviral treatment (ART) options for people living with HIV/AIDS (PLH). However, resistance-associated mutations (RAM) in reverse transcriptase (RT) and drug-drug-interactions (DDI) limit their use. The next-generation NNRTI Doravirine (DOR) was introduced in 2018 yet data on its use with DTG remain scarce.
**Methods:** Descriptive analysis of all PLH started on DOR/DTG (DoDo) at a university outpatient clinic.
**Results:** Through December 2020 28 PLH started DoDo. Median age was 56.5 years (range 19–79), female/male ratio was 7/21. HIV-associated/AIDS-defining conditions had been diagnosed in 17 (61%), the median CD4-nadir was 181/µl (range 4–543). The vast majority had an extensive ART history (table) with RPV/DTG as preceding regimen in 8 patients (29%). Protease-RAM were documented in 17 patients (61%), RT-RAM in 15 (54%), which were considered to affect other NNRTI in 11 (39%), resulting in off-label use of DOR. The main reasons for the switch to DoDo were tolerability (10, 36%), DDI (9, 32%), and cardiovascular risk (5, 18%); 17 patients (61%) received gastric acid inhibitors. Viral load (VL) was < 50 cp/ml in all patients, median CD4 count was 562.5/µl (range 298–1077). DoDo was stopped in 5 patients (18%) after a median of 3 months (range 0–11): in 4 due to symptoms persisting despite the switch, 1 returned to RPV/DTG after stopping pantoprazole. Twenty-three patients have remained on DoDo for a median of 8 months (range 1–22). In all patients VL has been continuously suppressed since the switch to DoDo.
**Conclusions:** DOR and DTG (DoDo) can be a valuable 2DR option also in heavily pretreated PLH. Further study of this well tolerated combination with low potential for DDI is warranted.

| Characteristic | total N=28 | n | (%) |
|---|---|---|---|
| **Sex** | female | 7 | 25% |
| | male | 21 | 75% |
| **Medical history** | CDC category B | 7 | 25% |
| | AIDS | 10 | 36% |
| **Age** [y] | (median, range) | 56.5 | (19 - 79) |
| **CD4 nadir** [/µl] | (median, range) | 181 | (4 - 543) |
| **Years on ART** | (median, range) | 21 | (1 - 28) |
| **# regimens** | (median, range) | 6 | (2 - 15) |
| **ART experience** | NRTI | 28 | 100% |
| | NNRTI | 24 | 86% |
| | PI | 21 | 75% |
| | INSTI | 24 | 86% |
| | T20 | 1 | 4% |
| | MVC | 0 | 0 |
| **Switch from** | RPV/DTG | 8 | 29% |
| **Documented RAM** | Reverse transcriptase | 15 | 54% |
| | affecting other NNRTI | 11 | 39% |
| | Protease | 15 | 54% |
| | Integrase | 0 | 0 |
| **Tropism testing** | CCR5 | 3 | 11% |
| **(V3 loop analysis)** | dual/mixed | 2 | 7% |
| | CXCR4 | 1 | 4% |

### 016
### Paper-ID: 47265, P3

### Dolutegravir based two-drug regimen in clinical routine

Hannes Höller[1], Hans Jäger[1], Celia Jonsson-Oldenbüttel[1], Silke Heldwein[1], Farhad Schabaz[1]; Ariane von Krosigk[1], Carmen Wiese[1], Dr. Eva Wolf[2], Sebastian Noe[1]
*[1]MVZ München am Goetheplatz; [2]MUC Research GmbH*

**Introduction:** Dolutegravir based two-drug regimens (2DRs) have demonstrated non-inferiority to three-drug regimens (3DR) in clinical trials. However, there is concern that results from these highly selected subjects do not allow generalization for clinical routine.
**Methods:** Retrospective analysis from a large HIV-center in Munich, Germany. People living with HIV (PLWH) on dolutegravir based two-drug (2DR) or three-drug (3DR) regimens were included into the analysis, with left-censoring for other dolutegravir-containing regimens for the 2DR group (i.e. a prior therapy with a 3DR was not considered for analysis in a patient with a 2DR treatment). Data after December 31st 2019 were censored.
**Results:** 912 PLWH were included into this analysis. Median age was 47 (IQR 38; 55) years, 730 (80.8%) were male, and 379 (42%) on a 2DR. Median time of observation was 17 (IQR 7; 41) months, in which 165 switches occurred; maximal observed times on 2DRs and 3DRs were 5.6 and 6 years, respectively. There were 45 (5%) PLWH with known M184V/I mutations at initiation of therapy, of which 28 (62.2%) were in the 3DR arm and 9 (20%) in the DTG/3TC stratum of the 2DR arm. Probability of discontinuation of the regimen under observation was significantly higher in the 3DR arm (p = 0.006). Median time on therapy was not achieved in any of the groups by the end of the current observational period. Discontinuation for suspected adverse drug effects occured in 16 (4.2%) and 72 (19%) (p < 0.001), for insufficient viral suppression in 2 (0.5%) and 14 (2.7%) (p = 0.031) in the 2DR and 3DR arms, respectively.
**Conclusion:** We found longer times until discontinuation of dolutegravir-based 2DRs when compared to 3DRs. Due to the design of the study, this cannot be explained by the switch of patients on 3DRs to 2DRs. In contrast, discontinuation for adverse effects was significantly less frequent in the 2DR group with no higher risk for insufficient viral suppression.

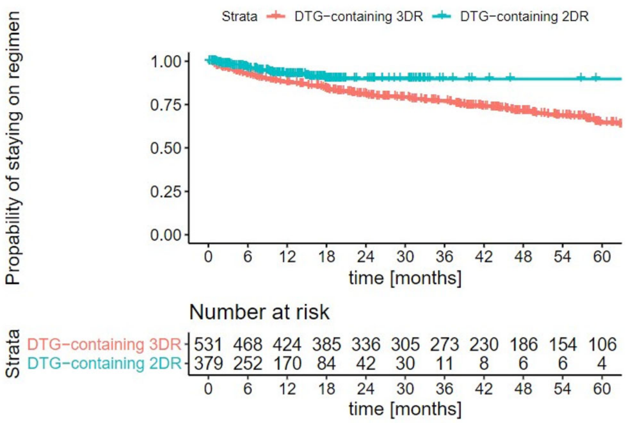

### 017
### Paper-ID: 46976, P4

### Significant increase in patients on dual ART due to new therapeutic regimens: Dual Therapy Regimens during 2016–2020

Heribert Knechten[1], Frank Wiesmann[1], Patrick Braun[1], Sascha Lengen[2], Wolfgang Köthemann[3], Daniel Gillor[3], Mechthild Knickmann[2], Anton Neuwirth[3], Petra Panstruga[1], Nazifa Quirishi[4], Katja Römer[4], Stefan Scholten[5]
*[1]PZB Aachen; [2]Insto GmbH; [3]MVZ Innere Medizin Köln; [4]Praxis Gotenring Köln, [5]Praxis Hohenstaufenring Köln*

**Background:** This retrospective cohort analysis focused on the proportion and treatment success of diverse dual treatment strategies between 2016 and 2020.

**Methods:** Within the North Rhine cohort, records of therapy-experienced patients from 16–19 centers were collected annually between 2016 (n = 2247) and 2020 (n = 4137). Data on therapy and virological response were documented for the first quarter of each year. Treatment success was determined as the proportion of patients with suppressed viral load < 50 copies/mL. Switch to dual therapies (2DR) occurred mostly in patients with prior successful viral suppression.
**Results:** During observation time, the overall proportion of 2DRs remained nearly constant until 2019. The proportion of patients on 2DR was 7.4% in 2016, 6.9% in 2017, 6.3% in 2018, and 6.6% in 2019. However, a significant increase in patients on 2DRs was observed in 2020, when the proportion increased to 14.2% (n = 588). The treatment success of dual regimens in therapy-experienced patients in 2016–2020 was 89.7%, 90.7%, 95.4%, 92.8%, and 94.3% respectively and was comparable to the treatment success in the entire cohort (91.2–96.4%). The proportion of patients on protease inhibitor (PI) + integrase inhibitor (INI) decreased from 64 to 41% in 2019 and to 20.1% in 2020. A similar trend was seen for the combination of nucleosidic reverse transcriptase inhibitors (NRTI) + PI (18–9% and 2.4%, respectively). On the other hand, combinations of NRTI + INI increased from 4 to 63.1% (mainly 3TC + DTG) and combinations of one non-nucleosidic reverse transcriptase inhibitors (NNRTI) + INI from 6% to 11.1%. In 2020, the most frequently documented combinations consisted of 3TC + DTG (62%), DRV/r + DTG (12.4%), RPV + DTG (10.2%), and DRV/r + RAL (4.6%).
**Conclusion:** In 2020, the proportion of patients on dual ART more than doubled in comparison to the same period of the previous year. In the four years prior, the proportion had remained fairly constant. Approximately 94% of all patients on dual therapy in 2020 showed a sustained viral load below the limit of detection which was comparable to the treatment success in the overall population.

### 018
### Paper-ID: 46763, P5

### Influences on treatment priorities among PLHIV—the link between specific treatment challenges and importance of various treatment goals

Alexandra Wigger[1], Bettina Berkowitsch[2], Ama Appiah[3], Rüdiger Allmann[1], Adam Stubbs[3], Chinyere Okoli[3], Patricia de los Rios[4]
*[1]ViiV Healthcare Munich Germany; [2]GSK Austria; [3]ViiV Brentford UK; [4]ViiV Quebec Canada*

**Introduction:** Little is known about how treatment challenges influence perceived treatment priorities. We investigated this key issue among people living with HIV (PLHIV).
**Methods:** Data were from 170 adult PLHIV from Austria (AT, 50) and Germany (DE, 120) in the Positive Perspectives 2 study. We assessed the relationship between perceived unmet need and treatment priorities using descriptive and multivariable analyses (Fig. 1).
**Results:** Mean age was 42.1 years and 60.0% [102/170] identified as men. Overall, 51.8% ever disguised their HIV medications (AT = 64.0% vs DE = 46.7%, p = 0.039); 37.1% experienced ART side effects; 34.1% felt daily ART cued bad memories; 31.8% had difficulty swallowing; and 24.1% were stressed by daily dosing. Furthermore, 28.8% [49/170] felt ART limited their life, and these PLHIV were more dissatisfied with treatment than those not feeling limited (44.9% [22/49] vs 28.1% [34/121], p = 0.035). Overall, 47.6% [81/170] preferred nondaily ART, and 68.2% [116/170] were open to ART with fewer medicines. Among those with HIV for ≥ 1 year (pooled n = 163), current treatment priorities were: minimizing side effects (54.6%), ensuring viral suppression (50.9%); minimizing potential for longterm effects (49.7%); managing HIV-related illnesses (38.0%); reducing number of medicines (37.4%), minimizing DDIs (36.8%), and increasing dosing flexibility (31.9%). Among these individuals diagnosed for ≥ 1 year, significant associations were seen between polypharmacy and goal of less DDIs (AOR = 8.43, 95% CI 3.45–20.55), and between presence of comorbidities and goal of managing HIV-related illnesses (AOR = 2.53; 95% CI 1.19–5.39). Switching of previous ARTs to reduce number of medicines was associated with current goal of reducing number of medicines (AOR = 3.18, 95% CI 1.06–9.51), vs those who never switched/switched for other reasons. Those worried about long-term ART impacts had 2.54 (1.26–5.14) higher odds of prioritizing the minimizing of long-term ART side effects vs those not worried.
**Conclusion:** There was a strong link between specific treatment challenges and perceived treatment priorities. Flexible regimens catering to diverse needs of PLHIV may improve treatment satisfaction.

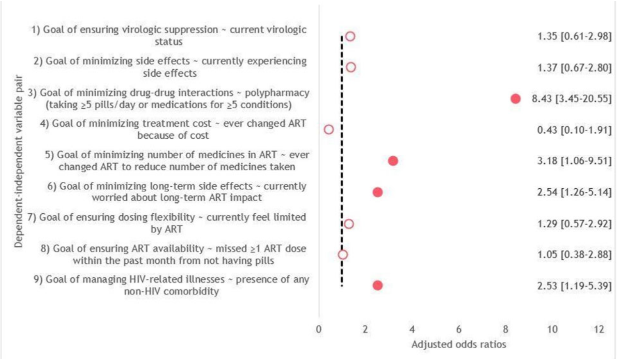

Note: Perceived treatment priorities/goals were those issues that PLHIV indicated they would consider as important if they were starting HIV treatment today. Hollow markers are statistically non-significant at $p < 0.05$. Analyses controlled for age, gender, sexual orientation, and housing type as a marker of socio-economic position (own/rent vs. shelter/shared housing/other).

### 019
### Paper-ID: 46753, P6

### Durable efficacy of dolutegravir (DTG) + lamivudine (3TC) in antiretroviral treatment–naive adults with HIV-1 infection—3 year results from the GEMINI studies

Pedro Cahn[1], Juan Sierra Madero[2], José Arribas[3], Andrea Antinori[4], Roberto Ortiz[5], Amanda Clarke[6], Chien-Ching Hung[7], Jürgen Rockstroh[8], Pierre-Marie Girard[9], Jörg Sievers[10], Choy Man[10], Rimgaile Urbaityte[11], Mark Underwood[10], Keith Pappa[10], Kimberly Smith[10], Martin Gartland[10], Michael Aboud[10], Jean van Wyk[10], Brian Wynne[10], Katharina Bernhardt[10]
*[1]Fundación Huésped; [2]Instituto Nacional de Ciencias Médicas y Nutrición Salvador Zubirán; [3]Hospital La Paz; [4]Istituto Nazionale per le Malattie Infettive Lazzaro Spallanzani; [5]Bliss Healthcare Services; [6]Royal Sussex County Hospital and Brighton & Sussex Medical School; [7]National Taiwan University Hospital; [8]Rheinische FriedrichWilhelms Universität; [9] Hôpital Saint Antoine; [10]ViiV Healthcare; [11]GlaxoSmithKline*

**Introduction:** Two-drug regimens may reduce cumulative drug exposure during lifelong HIV treatment. In GEMINI-1&-2 (NCT02831673/NCT02831764), DTG + 3TC was non-inferior to DTG + TDF/FTC at Wk48 and Wk96 in ART-naive adults.
**Methods:** GEMINI-1&-2 are identical, double-blind, multicentre, phase III studies. Participants with screening HIV-1 RNA ≤ 500,000 c/mL were randomised 1:1 (stratified by HIV-1 RNA and CD4+ count) to once-daily DTG + 3TC or DTG + TDF/FTC. Primary endpoint was proportion of participants with plasma HIV-1 RNA < 50c/mL at Wk48 (Snapshot algorithm). We present efficacy and safety data from prespecified 144-week secondary analyses.

Estimates and CIs were based on a stratified analysis using Cochran–Mantel–Haenszel weights.

**Results:** 714 and 719 adults were randomised and treated in GEMINI-1&-2, respectively. At baseline, 20% had HIV-1 RNA > 100,000 c/mL, 8% had CD4+ < 200 cells/mm$^3$. At Wk144, DTG + 3TC was noninferior to DTG + TDF/FTC in both GEMINI-1&-2 and the pooled analysis (Table). Response rates in participants with baseline HIV-1 RNA > 100,000 c/mL were high and similar between arms. Consistent with Wk48/96, response rates remained lower in DTG + 3TC participants with baseline CD4+ < 200 cells/mm$^3$. Across both studies, 12 DTG + 3TC participants (1 since Wk96) and 9 DTG + TDF/FTC participants (2 since Wk96) met protocol-defined confirmed virologic withdrawal (CVW) criteria through Wk144; none had treatment-emergent INSTI or NRTI resistance mutations. One non-CVW DTG + 3TC participant with reported non-adherence developed M184V (Wk132; HIV-1 RNA 61,927 c/mL) and R263R/K at Wk144 (135 c/mL), conferring a 1.8-fold change in DTG susceptibility. Overall AE rates were similar; rates of withdrawals due to AEs were low in both arms. Risk of drugrelated AEs was significantly lower with DTG + 3TC than DTG + TDF/FTC (20% vs 27%; relative risk ratio, 0.76; 95% CI 0.63–0.92). Post-baseline bone and renal biomarker changes favoured DTG + 3TC.

**Conclusions:** DTG + 3TC remains non-inferior to DTG + TDF/FTC in ART-naive adults at Wk144. Both regimens were well tolerated. Results demonstrate durable efficacy and potency of DTG + 3TC, further supporting it as a first-line HIV treatment option.

**Table.** Proportion of Participants With Plasma HIV-1 RNA <50 c/mL at Week 144: Snapshot Analysis (ITT-E Population)

| | | GEMINI-1 | GEMINI-2 | Pooled |
|---|---|---|---|---|
| Snapshot responders | DTG+3TC | 281/356 (79%) | 303/360 (84%) | 584/716 (82%) |
| | DTG+TDF/FTC | 296/358 (83%) | 303/359 (84%) | 599/717 (84%) |
| Adjusted difference (95% CI) | | −3.6 (−9.4, 2.1) | 0.0 (−5.3, 5.3) | −1.8 (−5.8, 2.1) |

10% non-inferiority margin.

### 020
### Paper-ID: 46754, P7

### Switching to DTG/3TC fixed-dose combination (FDC) is noninferior to continuing a TAF-based regimen (TBR) in maintaining virologic suppression through 96 weeks: TANGO study

Jean van Wyk[1], Faïza Ajana[2], Fiona Bisshop[3], Stéphane De Wit[4], Olayemi Osiyemi[5], Joaquín Portilla[6], Jean-Pierre Routy[7], Christoph Wyen[8], Mounir Ait-Khaled[1], Maria-Claudia Nascimento[1], Keith Pappa[1], Ruolan Wang[1], Jonathan Wright[9], Brian Wynne[1], Michael Aboud[1], Kimberly Smith[1], Martin Rubach[1]

*[1]ViiV Healthcare; [2]Centre Hospitalier de Tourcoing; [3]Holdsworth House Medical Brisbane; [4]CHU St-Pierre; [5]Triple O Research Institute PA; [6]Hospital General Universitario de Alicante; [7]McGill University Health Center; [8]Praxis am Ebertplatz; [9]GlaxoSmithKline*

**Introduction:** DTG/3TC FDC was non-inferior to TBRs through the Wk48 primary endpoint in TANGO. Here we present Wk96 secondary endpoint analyses.

**Methods:** TANGO, a randomized, open-label, non-inferiority phase III study, evaluates efficacy and safety of switching to once-daily DTG/3TC in virologically suppressed (> 6 months, no prior virologic failure [VF], no major NRTI or INSTI resistance) adults with HIV-1 vs continuing TBR, over 148 weeks. Participants were randomized 1:1, stratified by baseline third agent class. Wk96 analysis assessed non-inferiority for VF and virologic success (VS; Snapshot algorithm, ITT-E population).

**Results:** 741 randomized/exposed participants: DTG/3TC, 369; TBR, 372. For Snapshot VF, switching to DTG/3TC was non-inferior to continuing TBR at Wk96 (ITT-E analysis): 0.3% vs 1.1%; adjusted difference: − 0.8% (95% CI − 2.0%, 0.4%) and superior to TBR (per protocol analysis): 0% vs 1.1%; adjusted difference: − 1.1% (95% CI − 2.3, − 0.0); P = 0.044 (2-sided). Snapshot VS was high in both arms, demonstrating non-inferiority (Table). Forty-four participants (5.9%) had missing data in the Wk96 window due to COVID-19. No DTG/3TC participants and 3 TBR participants (< 1%) met protocol-defined VF with no resistance observed at failure. Overall AE rates were similar between arms, with more drug-related AEs with DTG/3TC (Table). TC, LDL-C, and triglycerides improved significantly with DTG/3TC; HDL-C changes significantly favored TBR, with no difference in TC/HDL-C ratio between arms. Decreases in GFR by cystatin C were observed, which were significantly lower with DTG/3TC; proximal tubular function marker changes were small and similar across arms.

**Conclusions:** At Wk96, switching to DTG/3TC FDC was non-inferior to continuing TBR in maintaining virologic suppression in ART-experienced adults. The safety profile of DTG/3TC FDC was consistent with DTG and 3TC respective labels. DTG/3TC FDC offers a robust switch option with durable efficacy, good safety and tolerability, and a high barrier to resistance with zero protocol-defined VF through Wk96.

**Table.** Efficacy and Key Safety Results for the ITT-E and Safety Populations

| **Week 96 study outcome by Snapshot analysis (ITT-E population), n (%)** | **DTG/3TC (N=369)** | **TBR (N=372)** |
|---|---|---|
| HIV-1 RNA ≥50 c/mL (Snapshot virologic failure) | 1 (0.3) | 4 (1.1) |
| HIV-1 RNA <50 c/mL (Snapshot virologic success)[a] | 317 (85.9) | 294 (79.0) |
| No virologic data in Week 96 window | 51 (13.8) | 74 (19.9) |
| **Week 96 virologic success for efficacy evaluable population,[b] n (%)** | **(N=353)** | **(N=344)** |
| HIV-1 RNA <50 c/mL (Snapshot virologic success) | 317 (89.8) | 294 (85.5) |
| **Key safety results (safety population), n (%)** | **(N=369)** | **(N=371[c])** |
| Any AEs | 324 (87.8) | 325 (87.6) |
| AEs or deaths leading to withdrawal | 21 (5.7) | 4 (1.1) |
| Drug-related grade 2-5 AEs[d] | 21 (5.7) | 7 (1.9) |
| Serious AEs | 42 (11.4) | 35 (9.4) |

[a]Snapshot virologic success adjusted difference in (DTG/3TC) − TBR: 6.8% (95% CI: 1.4%, 12.3%). Estimates and confidence intervals were based on a stratified analysis using Cochran-Mantel-Haenszel weights adjusting for baseline third agent class (PI, NNRTI, INSTI). Non-inferiority for virologic failure and virologic success assessed using 4% and 8% non-inferiority margins, respectively.
[b]Sensitivity analysis excluding 16 and 28 participants in the DTG/3TC and TBR arms, respectively, because of no Week 96 HIV-1 RNA data due to COVID-19 pandemic impact. Snapshot virologic success adjusted difference in (DTG/3TC) − TBR: 4.3% (95% CI: −0.6%, 9.2%).
[c]1 participant was excluded due to receiving a TDF-based regimen instead of a TAF-based regimen
[d]2 deaths (1 homicide and 1 unknown reason) both unrelated to treatment occurred in the DTG/3TC arm.

### 021
### Paper-ID: 46788, P8

### Feasibility, efficacy, and safety of using dolutegravir/lamivudine (DTG/3TC) as a first-line regimen in a test-and-treat setting for newly diagnosed people living with HIV (PLWH): the STAT study

Charlotte-Paige Rolle[1], Mezgebe Berhe[2], Tulika Singh[3], Roberto Ortiz[4], Anson Wurapa[5], Moti Ramgopal[6], Peter A. Leone[7], Jessica E. Matthews[8], Marybeth Dalessandro[9], Mark R. Underwood[8], Konstantinos Angelis[9], Brian R. Wynne[8], Deanna Merrill[8], Christopher Nguyen[8], Jean van Wyk[8], Kathrin M. Dymek[8]

*[1]Orlando Immunology Center; [2]Baylor University Medical Center; [3]Desert AIDS Project; [4]Bliss Healthcare Services; [5]Infectious Disease Specialists of Atlanta; [6]Midway Immunology and Research Center; [7]School of Medicine, University of North Carolina at Chapel Hill; [8]ViiV Healthcare; [9]GlaxoSmithKline*

**Introduction:** DTG/3TC is indicated for treatment-naive PLWH. Questions remain about DTG/3TC in rapid-initiation settings because of potential transmitted resistance and HBV co-infection. STAT evaluates feasibility and efficacy of DTG/3TC in a US test-and-treat setting.

**Methods:** STAT is a single-arm pilot study wherein participants initiated DTG/3TC ≤ 14 days after HIV1 diagnosis without available screening/baseline laboratory results. Treatment was adjusted if results indicated HBV, DTG or 3TC resistance, or creatinine clearance < 30 mL/min/1.73 $m^2$; participants with treatment adjustment remained on study. Efficacy endpoints were proportions of participants with HIV-1 RNA < 50 c/mL at Wk24 regardless of ART among all participants (including those with missing data;'Intention-to-treat-exposed (ITT-E) Missing = Failure' analysis) and those with available Wk24 data ('Observed' analysis).

**Results:** Sixteen sites enrolled 131 participants: median age, 31 years; 8% female; 50% non-white. At baseline, 39%, 15% and 8% had HIV-1 RNA ≥ 100,000, ≥ 500,000 and ≥ 1,000,000 c/mL, respectively; 28% had CD4+ T-cell count < 200 cells/$mm^3$. Median time to enrollment from initial diagnosis was 5 days. Through Wk24, 8 participants had treatment modified: 5 for baseline HBV, 1 for baseline M184V, and 2 for other reasons (AE [rash] and participant withdrawal). Among all participants and those with available data, 78% and 92% achieved HIV-1 RNA < 50/mL, respectively (Table). All participants with available data who switched ART and remained on study at Wk24 had HIV-1 RNA < 50 c/mL. The participant with baseline M184V achieved HIV-1 RNA < 50 c/mL by Wk4 before regimen modification. Among 10 participants with baseline HIV-1 RNA ≥ 1,000,000 c/mL, 8 had HIV-1 RNA < 50 c/mL at Wk24 (1 discontinued before Wk24; 1 had HIV-1 RNA ≥ 50 c/mL).

**Conclusions:** Results demonstrate that first-line DTG/3TC is feasible and safe in a test-and-treat setting; appropriate adjustments for baseline resistance or HBV co-infection can occur safely via routine clinical care after rapid DTG/3TC initiation.

**Table.** Proportion of Participants With HIV-1 RNA <50 and <200 c/mL at Week 24 in Observed and ITT-E Missing=Failure Analyses

| Outcomes | DTG/3TC, n/N (%) |
|---|---|
| **Observed analysis (ie, participants with HIV-1 RNA data at Week 24 on any ART)** | |
| Participants with available HIV-1 RNA data at Week 24 | 111/131 (85) |
| HIV-1 RNA <50 c/mL | 102/111 (92)[a] |
| HIV-1 RNA <200 c/mL | 109/111 (98)[b] |
| **ITT-E Missing=Failure analysis at Week 24 (ie, all participants)** | |
| HIV-1 RNA <50 c/mL | 102/131 (78) |
| ART received at Week 24: on DTG/3TC | 97/131 (74) |
| ART received at Week 24: on modified ART[c] | 5/131 (4) |
| HIV-1 RNA ≥50 c/mL | 29/131 (22) |
| Data in window and HIV-1 RNA ≥50 c/mL | 9/131 (7) |
| On study but missing data in window | 5/131 (4)[d] |
| Discontinued study due to LFU/withdrew consent | 12/131 (9)[e] |
| Discontinued study for other reasons | 3/131 (2)[f] |

ART, antiretroviral therapy; DTG/3TC, dolutegravir/lamivudine; ITT-E, intention-to-treat–exposed; LFU, lost to follow-up.
[a]Of 102 participants, 97 were on DTG/3TC regimen. [b]Of 109 participants, 104 were on DTG/3TC regimen. [c]3 participants on modified ART were missing data at Week 24. [d]3 participants missed HIV-1 RNA assessment at Week 24 due to COVID-19. [e]7 due to LFU; 5 withdrew consent (3 relocations; 1 incarceration; 1 no reason reported). [f]3 due to physician decision (2 HIV-negative, 1 participant did not attend several scheduled appointments).

### 022
### Paper-ID: 46807, P9

### Is DTG + 3TC effective and safe in clinical practice? Evidence from real-world data

Yogesh Punekar[1], Daniel Parks[2], Jean van Wyk[1], Annemiek de Ruiter[1], Christina Schröder[3]
*[1]ViiV Healthcare; [2]GlaxoSmithKline; [3]ViiV Healthcare GmbH*

**Introduction:** Evidence from randomized controlled trials (RCTs) has shown that dolutegravir (DTG) + lamivudine (3TC) is an efficacious, safe, and durable regimen in treatment-naive and treatment experienced people living with HIV-1 (PLHIV). Several observational studies have also concluded that it is effective in clinical practice. The objective of this meta-analysis was to estimate effectiveness and safety of DTG + 3TC in treatment-experienced, virologically suppressed PLHIV by combining real world evidence (RWE) from clinical practice.

**Methods:** A systematic literature review of PubMed and Embase, along with 24 regional and international conferences, was conducted between January 2013 and December 2019 to identify non-RCT studies of DTG + 3TC in PLHIV. Eligible published articles presenting outcomes of interest were identified and extracted. Identified studies were included if they had acceptable levels of publication bias and heterogeneity, which were determined using funnel plots and I2 statistics, respectively. One-arm meta-analyses using the DerSimonian and Laird method were conducted to estimate effect sizes for virologic failure, virologic suppression, and discontinuations for DTG + 3TC.

**Results:** A total of 7 DTG + 3TC studies (N = 1800 PLHIV) reported data on treatment-experienced, virologically suppressed PLHIV for outcomes of interest at different time points. Results showed that among PLHIV switching to DTG + 3TC treatment, ≥ 90% maintained virologic suppression (ITT) with ≤ 1% virologic failures on DTG + 3TC.

**Conclusions:** DTG + 3TC is an effective, safe, and durable antiretroviral regimen with low rates of discontinuation in treatment-experienced PLHIV in clinical practice.

**Table.** Proportion of PLHIV With Virologic Failure, Virologic Suppression, and Discontinuations at Weeks 48 and 96

| | DTG + 3TC | | | |
|---|---|---|---|---|
| **Week 48** | Virologic failure (N=1800) | Virologic suppression ITT (N=1800) | Virologic suppression PP (N=1552) | Discontinuations (N=1800) |
| Week 48, mean (95% CI) | 0.008 (0.004-0.014) | 0.906 (0.849- 0.951) | 0.990 (0.983- 0.995) | 0.089 (0.048-0.139) |
| **Week 96** | Virologic failure (N=904) | Virologic suppression ITT (N=904) | Virologic suppression PP (N=767) | Discontinuations (N=904) |
| Week 96, mean (95% CI) | 0.005 (0.001-0.013) | 0.930 (0.831- 0.990) | 0.995 (0.976- 1.000) | 0.057 (0.004-0.151) |

CI, confidence interval; ITT, intention-to-treat; PP, per protocol.

### 023
### Paper-ID: 46808, P10

### Impact of treatment adherence on efficacy of DTG + 3TC and DTG + TDF/FTC: pooled analysis of the GEMINI-1 and -2 clinical studies

Mounir Ait-Khaled[1], Juan Sierra Madero[2], Vicente Estrada[3], Roberto Gulminetti[4], Debbie Hagins[5], Hung-Chin Tsai[6], Choy Man[1], Jörg Sievers[1], Rimgaile Urbaityte[7], Richard Grove[7], Andrew Zolopa[1], Brian Wynne[1], Jean van Wyk[1], Harald Fetscher[8]
*[1]ViiV Healthcare; [2]Instituto Nacional de Ciencias Médicas y Nutrición Salvador Zubirán; [3]Ciudad Universitaria; [4]Institute of Infectious Diseases, University of Pavia; [5]Georgia Department of Public Health, Coastal Health District, Chatham CARE Center; [6]Kaohsiung Veteran General Hospital; [7] GlaxoSmithKline; [8]ViiV Healthcare GmbH*

**Introduction:** GEMINI-1&-2 are double-blind, multicenter, phase III, non-inferiority studies evaluating efficacy and safety of once-daily DTG + 3TC in treatment-naive adults with screening HIV-1 RNA ≤ 500,000 c/mL (NCT02831673/NCT02831764). Participants were randomized 1:1 to DTG + 3TC or DTG + TDF/FTC. The primary endpoint was the proportion of participants with plasma HIV-1 RNA < 50 c/mL at Week 48 (Snapshot algorithm). DTG + 3TC was non-inferior to DTG + TDF/FTC at Weeks 48 and 96. Here we evaluate the impact of treatment adherence on Week 48 virologic response (VR) within the GEMINI trials as a post-hoc analysis.

**Methods:** Adherence was estimated using pill count data and categorized as ≥ 90% vs < 90%. Week 48 VR was measured as percentage of participants with HIV-1 RNA < 50 c/mL by FDA Snapshot and by last on-treatment viral load (VL) for ITT-E population for whom adherence could be derived. VR and differences between treatment arms within each adherence category were calculated along with exact unadjusted 95% confidence intervals.

**Results:** 5% of participants had < 90% adherence in both treatment arms. Baseline VL and CD4+ cell counts were similar across adherence categories. VR was lower in the < 90% adherence group vs ≥ 90% group but not different between the 2 treatment arms within the same adherence category: in the < 90% adherence group, DTG + 3TC VR was 69% vs 65% for DTG + TDF/FTC by Snapshot and 91% and 85%, respectively, by last on-treatment VL analysis (Table).

**Conclusions:** In the GEMINI studies, a lower Week 48 VR was observed in participants with < 90% adherence, but the impact of lower adherence on VR was similar in the DTG + 3TC compared with DTG + TDF/FTC arms. One limitation of the analysis was the small number of participants in the lower adherence subgroup. However, results provide additional information on the robustness of DTG + 3TC compared with 3-drug DTG-containing regimens and may suggest similar regimen forgiveness.

**Table.** Virologic Response (Using Snapshot at Week 48 or Last on Treatment VL) by Adherence Category (ITT-E Population[a])

| Efficacy endpoint | Adherence level category | DTG + 3TC n/N (%; 95% CI) | DTG + TDF/FTC n/N (%; 95% CI) | Treatment difference[b] (%; 95% CI) |
|---|---|---|---|---|
| HIV-1 RNA <50 c/mL (Snapshot) | ≥90% | 631/679 (93%; 90.7-94.7) | 647/677 (96%, 93.7-97.0) | −2.6% (−7.9%, 2.7%) |
| | <90% | 24/35 (69%; 50.7-83.1) | 22/34 (65%; 46.5-80.3) | 3.9% (−20.4%, 26.2%) |
| HIV-1 RNA <50 c/mL (last on treatment VL) | ≥90% | 661/679 (97%; 95.8-98.4) | 668/677 (99%; 97.5-99.4) | −1.3% (−6.7%, 4.1%) |
| | <90% | 32/35 (91%; 76.9-98.2) | 29/34 (85%; 68.9-95.0) | 6.1% (−17.6%, 28.8%) |

CI, confidence interval; DTG, dolutegravir; FTC, emtricitabine; ITT E, intention to treat exposed; 3TC, lamivudine; TDF, tenofovir disoproxil fumarate; VL, viral load. [a]Excluding 2 and 6 participants in the DTG + 3TC and the DTG + TDF/FTC treatment arms, respectively, for whom no adherence could be derived. [b]DTG + 3TC response rate - DTG + TDF/FTC response rate.

### 024
### Paper-ID: 46943, P11

### Characterization of treatment-naïve people living with HIV starting on antiretroviral treatment under real-life conditions—results from the German AURORA Study

Janina Bruening[1], Benedikt Funke[1], Christiane Cordes[2], Christoph Wyen[3], Christoph Stephan[4], Juergen Rockstroh[5], Nils Postel[6], Ramona Pauli[7], Christine Koegl[8], Vanessa Witte[1]

*[1]MSD Sharp & Dohme GmbH, Medical Affairs; [2]Praxis Dr. Cordes; [3]Praxis am Ebertplatz; [4]HIV-Center/Universitätsklinikum Frankfurt; [5]Medizinische Klinik und Poliklinik I Klinisches Studienzentrum Immunologie; [6]prinzmed; [7]Isarpraxis; [8]MUC Research, Clinical Research*

**Introduction:** The AURORA study characterized the profile of treatment-naïve people living with HIV (PLWH) in a real-world setting in Germany, aiming to elucidate factors that physicians have to take into account when selecting the optimal initial antiretroviral treatment (ART).

**Methods:** AURORA is a national, multi-center (n = 22), retrospective chart review to characterize the profile of treatment-naïve PLWH starting ART between 2017 and 2020. Items of interest included sociodemographic variables, co-morbidities, co-medication, vital signs, routine and HIV-related laboratory parameters, ART and reasons for ART choice (multiple responses).

**Results:** N = 354 patients were included (2017: N = 12; 2018: N = 115; 2019: N = 149; 2020: N = 78): female 16%, median age 38 years, Caucasian 85%, MSM 67%, late presenter (LP) 54% (CD4 abs. < 350/µl and/or AIDS def. events), median CD4 count 352/µL (IQR interquartile range: 153–556/µL), HIV-RNA > 100,000 copies/mL 46%. LP were more common in 2019 and 2020 than in 2018 (60%, 56%, vs 46%, respectively). 4% of LP (8/192) and 14% of non-LP (23/162) received dual therapy. While most common reasons for specific ART-choice were convenience, virologic control and tolerability, the choice of dual therapy for nonLP was clearly driven by tolerability (96%). Differences with respect to late presentation are shown in Fig. 1. Initial ART was INSTI-based in 84%, PI-based in 8% and NNRTI-based in 6% of patients; most common regimens were BIC/TAF/FTC (57%) followed by DTG/3TC (8%), DTG/ABC/3TC (7%) and DRV/COBI/TAF/FTC (6%). Differences of > 5%-points between LP and nonLP were seen for B/F/TAF (63% vs 51%), DTG/3TC (3% vs 14%) and D/C/F/TAF (9% vs 2%).

**Conclusions:** Within the previous three years, treatment initiation with advanced HIV-disease has still been common among PLWH in Germany (> 50% late presenters). ART-choice was driven by efficacy and likewise by patient factors such as tolerability and convenience. New drug-approvals in recent years were reflected in prescription patterns.

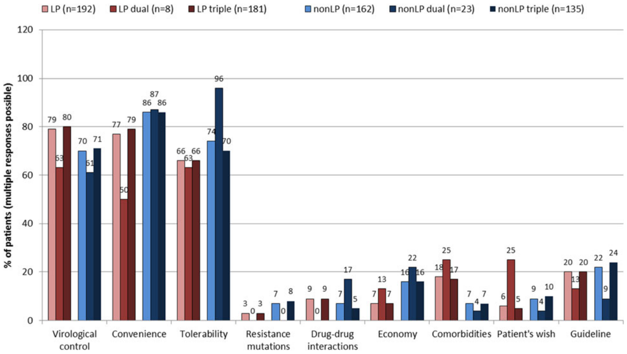

### 025
### Paper-ID: 46813, P12

### A daily single tablet regimen of bictegravir/emtricitabine/tenofovir alafenamide in virologically-suppressed adults living with HIV and end stage renal disease

Ansgar Rieke[1], Joseph J. Eron[2], Aimee Wilkin[3], Moti Ramgopal[4], Olayemi Osiyemi[5], Mehri McKellar[6], Jihad Slim[7], Polina German[8], Chris Blair[8], Christoph C. Carter[8], Diana M. Brainard[8], Sean E. Collins[8], Hal Martin[8]

*[1]Gemeinschaftspraxis Koblenz-Mayen Kemperhof; [2]The University of North Carolina at Chapel Hill; [3]Wake Forest University; [4]Midway Research Center; [5]Triple O Research Institute PA; [6]Duke University Medical Center; [7]Saint Michael's Medical Center; [8]Gilead Sciences Inc.*

**Introduction:** Treatment for people living with HIV (PLWH) and end stage renal disease (ESRD) on hemodialysis (HD) has previously required complex dose-adjusted regimens. We evaluated a daily regimen of elvitegravir/cobicistat/emtricitabine/tenofovir alafenamide (E/C/F/TAF) and established this treatment as effective and safe. After week (W)96, participants transitioned to daily B/F/TAF to assess whether efficacy and safety would be maintained on this STR that is guidelines-recommended for PLWH with eGFR > 30 mL/min.

**Methods:** Virologically suppressed adult PLWH with ESRD on chronic HD who completed W96 on E/C/F/TAF enrolled in the B/F/TAF extension for 48 weeks. Efficacy was assessed as the proportion of participants with HIV RNA < 50 copies/mL. Safety was assessed throughout the study, PK was assessed using sparse sampling at W4, 24 and 48.

**Results:** 55 enrolled, 36 completed E/C/F/TAF, 10 entered the B/F/TAF extension. The median age was 55yrs (range 34–63); median time on HD was 4yrs (range 2–16). All ten participants on B/F/TAF had HIV-1 RNA < 50 c/mL at W48. All participants had at least 1 adverse event (AE); one participant had a grade 3 AE and 3 had serious AEs; none were considered related to study drug. One participant had AEs attributed to study drug (grade 1 and 2), which resolved and did not lead to discontinuation of study drug. There were no clinically relevant changes in fasting lipids. In participants with evaluable data, mean bictegravir trough concentrations were lower compared to PLWH not on HD but remained 4- to 7-fold higher than the established protein-adjusted 95% effective concentration against wild-type virus.

**Conclusions:** Daily regimen of B/F/TAF maintained virologic suppression in PLWH on chronic HD. B/F/TAF was well-tolerated with no discontinuations. B/F/TAF may be an effective, safe and convenient once daily STR and ameliorate the need for dose adjustment in appropriate PLWH who require chronic HD.

### 026
### Paper-ID: 46951, P13

### Starting or switching to bictegravir/emtricitabine/tenofovir alafenamide (B/F/TAF) in clinical practice: pooled 12-month (12M) results from the global BICSTaR study

Christoph Spinner[1], Albrecht Stöhr[2], Alexander Wong[3], Joss de Wet[4], Jérémy Zeggagh[5], Laurent Hocqueloux[6], Berend van Welzen[7], Marion Heinzkill[8], Sabrinel Sahali[9], Almudena Torres Cornejo[9], Heribert Ramroth[10], Richard Haubrich[11], David Thorpe[10], Connie Kim[12]
*[1]Klinikum rechts der Isar-School of Medicine, Technical University of Munich; [2]IFI Studien und Projekte GmbH; [3]Department of Medicine, University of Saskatchewan; [4]Spectrum Health; [5]Service des Maladies Infectieuses, Hôpital Saint Louis, APHP; [6]Service des Maladies Infectieuses et Tropicales, CHR d'Orléans; [7]University Medical Centre Utrecht; [8]Gilead Sciences GmbH; [9]Gilead Sciences; [10]Gilead Sciences Ltd; [11]Gilead Sciences Inc.; [12]Gilead Sciences Canada Inc*

**Introduction:** The ongoing observational BICSTaR study aims to demonstrate effectiveness, safety and tolerability of B/F/TAF in routine clinical practice in at least 1400 antiretroviral treatment (ART)naïve (TN) and ART-experienced (TE) people living with HIV (PLHIV).

**Methods:** This 12M analysis of PLHIV receiving B/F/TAF in Europe and Canada assessed HIV1-RNA (missing data = excluded analysis), drug-related (DR) adverse events (AEs), persistence and weight/body-mass index (BMI) change.

**Results:** At the time of data cut-off (Mar 2020), 513 participants (n = 84 TN/n = 429 TE) completed 12M visit. Most were male (91%) and white (89%); median age was 38 (TN) and 49 (TE) years. Prevalence of comorbidities at baseline was 76%. 71%/18%/13% of TE participants switched from.

INSTI/NNRTI/PI-based regimens, respectively (26% TDF); 8% had a history of prior virologic failure. Baseline primary resistance prevalence by historical genotype was 9% (n = 43/513; 5% had resistance mutations associated with NNRTIs, 3% PIs, 3% NRTIs and 0.2% with INSTIs). At M12, 100% of TN (n = 74/74) and 96% (n = 357/373) TE participants had viral load (VL) < 50 copies/ml. Comparable effectiveness was observed in both male and female participants, including older individuals. No major resistance substitutions to the components of B/F/TAF emerged.

DRAEs occurred in 14% (n = 12/84) of TN and 15% (n = 64/429) of TE participants, discontinuations due to DRAE were low (TN 3.6% and 7.2% TE) and 90% of study participants remained on B/F/TAF (n = 462/513). Serious DRAEs were rare (0.4%; all TE).

At 12M, median (Q1, Q3) weight change was + 2.5 kg (0.5, 6.3) for TN (n = 48) and + 0.9 kg (− 1.0, 3.0) for TE (n = 269), with small changes in BMI of + 0.8 kg/m$^2$ (0.1, 1.9) for TN and + 0.3 kg/m$^2$ (− 0.3, 1.0) for TE.

**Conclusions:** The use of B/F/TAF in this real-world clinical cohort was associated with a high level of effectiveness and safety through 12M, inclusive of male, female and older PLHIV.

**Table.** Effectiveness and BMI categories

| Effectiveness at 12M (HIV RNA <50 copies/ml) | TN (n=84) | | TE (n=429) | | |
|---|---|---|---|---|---|
| Overall, % (n/N) | 100 (74/74) | | 96 (357/373) | | |
| Female, % (n/N) | 100 (6/6) | | 97 (29/30) | | |
| Male, % (n/N) | 100 (68/68) | | 96 (328/343) | | |
| ≥50 years of age, % (n/N) | 100 (16/16) | | 93 (170/182) | | |
| <50 years of age, % (n/N) | 100 (58/58) | | 98 (187/191) | | |
| Baseline resistance mutations, % (n/N) | | | | | |
| ○ M184V/I | - | | 100 (8/8) | | |
| ○ K65R | 100 (1/1) | | - | | |
| ○ G140S | - | | 0 (0/1)* | | |
| **BMI categories+** | **TN (n=48)** | | **TE (n=269)** | | |
| | Baseline | 12M | Baseline | 12M | |
| Underweight, <18.5 kg/m², % (n) | 6 (3) | 2 (1) | 2 (6) | 1 (3) | |
| Normal, 18.5–24.9 kg/m², % (n) | 60 (29) | 58 (28) | 49 (132) | 47 (125) | |
| Overweight, 25–29.9 kg/m², % (n) | 25 (12) | 29 (14) | 34 (91) | 37 (99) | |
| Obese ≥30 kg/m², % (n) | 8 (4) | 10 (5) | 15 (40) | 16 (42) | |

* M12 viral load was 71 copies/ml and participant was still on B/F/TAF treatment

+ BMI category according to WHO's BMI classification

### 027
### Paper-ID: 46968, P14

### Effectiveness and persistence of E/C/F/TAF, R/F/TAF or F/TAF + 3rd agent in late and very late presenters—final 24 month results from the German TAFNES cohort study

Nils Postel[1], Heiko Jessen[2], Ramona Pauli[3], Heribert Hillenbrand[4], Ansgar Rieke[5], Heribert Knechten[6], Hans-Jürgen Stellbrink[7], Claudia Emmerich[8], Sandra Schreiber[8], Frank Mack[8], Richard Haubrich[9]
*[1]Prinzmed; [2]Praxis Jessen2 + Kollegen; [3]Gemeinschaftspraxis Becker/Pauli; [4]MVZ Praxis City Ost; [5]Gemeinschaftspraxis Koblenz-Mayen Kemperhof; [6]PZB; [7]ICH Study Center Hamburg; [8]Gilead Sciences GmbH; [9]Gilead Sciences Inc.*

**Introduction:** The prospective TAFNES cohort was initiated to provide data on effectiveness and safety of emtricitabine/tenofovir alafenamide (F/TAF)-based regimens in routine clinical care in Germany. Here we present the final 24-month (M24) outcomes in ART-naïve PLWH (people living with HIV) comparing late presenters (LP; CD4 < 350/µL and/or history of AIDS) (subgroup: LP with advanced disease (LPAD; CD4 < 200/µL and/or AIDS) with PLWH in earlier disease stages (nonLP).

**Methods:** M24 evaluation of PLWH on F/TAF-based ART with elvitegravir/cobicistat (E/C/F/TAF) or rilpivirine (R/F/TAF) or F/TAF + 3rd agent: Effectiveness outcomes comprise viral response (HIV-RNA < 50 cp/mL) and study/study drug persistence. Differences in viral response were tested for significance applying logistic regression. Other outcomes included: non-serious/serious adverse drug reactions (ADRs/SADRs) and health-related-quality of life (HRQL) using SF-36 and HIV Symptom Index (HIV-SI).

**Results:** N = 296 ART-naive PLWH were eligible for M24 analysis (94% men, 9% CDC-C, median age 37 years), 105 of whom were LP (35%; 56 LPAD [19%]). 156 patients received E/C/F/TAF (30% LP), 41 received R/F/TAF (22% LP), and 99 received F/TAF + 3rd agent (49% LP). At M24, overall virologic response rate was 73% (n = 177/241), with no significant difference between LP (69%, n = 62/90; LPAD 60%, n = 28/47) and nonLP (76%, n = 115/151). Study/study drug persistence through M24 in LP and nonLP was 74% (LPAD 69%) and 80%, respectively. Discontinuations and documented ADRs are shown in the Table with low rates of discontinuation due to ADRs or virologic failure. Changes in HRQL scores reflect improvements within all subgroups for HIV-SI and in LP and LPAD for SF-36.

**Conclusions:** In TAFNES cohort on initial F/TAF-based ART, late presenters had similar high retention and virologic response rates as non-late presenters at M24. Discontinuations due to virologic failure or ADR were infrequent in both groups. Improvements in HRQL were observed in late presenters, particularly in those with advanced disease.

**Table.** Reasons for discontinuation of the study and/or study medication, documented ADRs/SADRs and changes in HRQL (in the subgroup with Baseline (BL) and month 24 (M24) data).

| n (%) | Overall (n=296) | LP (n=105) | Subgroup of LP: LPAD (n=56) | nonLP (n=191) |
|---|---|---|---|---|
| Discontinuations by month 24, n (%) | 101 (34) | 38 (36) | 24 (43) | 63 (33) |
| Due to | | | | |
| Therapy simplification | 13 (4.4) | 8 (7.6) | 5 (8.9) | 5 (2.6) |
| ADRs | 11 (3.7) | 6 (5.7) | 4 (7.1) | 5 (2.6) |
| Virologic failure | 4 (1.4) | 0 (0.0) | 0 (0.0) | 4 (2.1) |
| due to lost to follow-up | 41 (13.9) | 13 (12.4) | 8 (14.3) | 28 (14.7) |
| Documented ADRs*, events/patients (% of patients) | 19/14 (4.7) | 10/8 (7.6) | 3/3 (5.4) | 9/6 (3.1) |
| Documented serious ADRs (SADRs), events/patients (% of patients) | 0/0 (0) | 0/0 (0) | 0/0 (0) | 0/0 (0) |
| Change HIV-SI[1] from BL until M24, mean (+/-SD) [n] | -3.5 (11.0) [126] | -4.4 (11.4) [44] | -5.9 (12.1) [20] | -3.0 (10.9) [82] |
| Change SF36[2] physical component score from BL until M24, mean (+/-SD) [n] | +2.0 (9.1) [127] | +3.4 (8.8) [43] | +7.4 (9.6) [19] | +1.2 (9.2) [84] |
| Change SF36[2] mental component score from BL until M24, mean (+/-SD) [n] | +3.5 (12.3) [127] | +6.6 (12.4) [43] | +6.7 (8.7) [19] | +1.8 (12.0) [84] |

* In another 4 patients (3 nonLP and 1 LPAD) ADRs were not documented as ADR in eCRF, but documented as reason for discontinuation;
[1] range 0-80, higher scores indicate more bothering symptoms, [2]norm based scoring, higher scores indicate higher HRQL

### 028
### Paper-ID: 46973, P15

### Patient-reported outcomes after 1 year of routine clinical practice with bictegravir/emtricitabine/tenofovir alafenamide in people living with HIV: the BICSTaR cohort

Heribert Knechten[1], Christoph Stephan[2], Michael Waizmann[3], Jason Brunetta[4], Benoit Trottier[5], Claudine Duvivier[6], Berend van Welzen[7], Frank Mack[8], Harout Tossonian[9], David Thorpe[10], Almudena Torres Cornejo[11], Andrea Marongiu[10], Rafael Curbelo[11]
*[1]PZB; [2]University Hospital Frankfurt, Medical Clinic II; [3]Praxis Waizmann; [4]Maple Leaf Medical Clinic; [5]Clinique médicale urbaine du Quartier latin; [6]Service de Maladies Infectieuses et Tropicales, Centre d'Infectiologie NeckerPasteur, APHP-Hôpital Necker-Enfants maladies; [7]University Medical Centre Utrecht; [8]Gilead Sciences GmbH; [9]Gilead Sciences Canada Inc.; [10]Gilead Sciences Ltd; [11]Gilead Sciences*

**Introduction:** Patient-reported outcome (PRO) measures are directly completed by the patient to capture aspects of patient's health, such as mental health status, health related quality of life (HRQoL) and treatment satisfaction. The observational BICSTaR study prospectively collected PROs in PLWH initiating or switching to B/F/TAF.
**Methods:** PRO analysis from the BICSTaR study in antiretroviral-treatment naïve (TN) and treatmentexperienced (TE) participants from Germany, Canada, France and the Netherlands who completed PRO questionnaires at both baseline (BL) and month 12 (M12). PRO measurements included HRQoL (SF-36: Physical [PCS] and Mental Component Score [MCS]), health status (HIV-Symptom Index [HIVSI]), and patient satisfaction (HIVTSQs and HIVTSQc in TE only).
**Results:** Availability of PRO data at BL and M12 follow-up visits varied by instrument and treatment group. Participants were mainly male and TN were younger than TE participants, median age 38 vs 49 years, respectively. At baseline, mean summary scores in TN were PCS: 53.7 (standard deviation [SD]: 6.3) and MCS: 54.1 (7.4); PCS remained stable and MCS increased by a mean of 0.2 (10) by M12. In TN participants the most frequently reported bothersome symptom at BL was fatigue (69%). The frequency of bothersome symptoms decreased after M12 on B/F/TAF (Table 1). In TE patients, mean summary scores at baseline were PCS: 54.1 (7.4) and MCS: 46.8 (12.5); these remained stable at M12. The most frequently reported bothersome symptom at BL was fatigue (48%). Baseline HIVTSQs total score was high in TE, median 56 (50, 60), with further improvements following switch to B/F/TAF at M12, with an HIVTSQc median total score change of 20.
**Conclusions:** Analysis of PROs from BICSTaR showed that the greatest improvements from baseline after 12 months of B/F/TAF treatment were seen in the HRQoL MCS and in most commonly reported symptoms among the TN population and in treatment satisfaction among TE.

**Table.** HRQoL ((SF-36), HIV-SI, HIVTSQs total score and HIVTQc total score change results at baseline and month 12 by treatment naïve and experiences patients.

| PRO Measure | Treatment naïve | Treatment experienced |
|---|---|---|
| **SF-36* n** | **38** | **183** |
| Physical component score (PCS) | | |
| Baseline, mean (95% CI) | 53.7 (51.7, 55.8) | 54.1 (53.0, 55.2) |
| Month 12, mean (95% CI) | 54.0 (51.7, 56.2) | 53.8 (52.7, 54.8) |
| BL-M12 Δ change, mean (95% CI) | Δ 0.2 (-2.2, 2.7) | Δ -0.3 (-1.3, 0.6) |
| Mental component score (MCS) | | |
| Baseline, mean (95% CI) | 42.9 (39.1, 46.8) | 46.8 (45.0, 48.6) |
| Month 12, mean (95% CI) | 47.6 (44.1, 51.1) | 48.1 (46.4, 49.7) |
| BL-M12 Δ change, mean (95% CI) | Δ 4.7 (1.4, 7.9) | Δ 1.3 (0.0, 2.5) |
| **HIV-SI bothersome symptoms n** | **42** | **207** |
| Fatigue or loss of energy | | |
| Baseline, n (%) | 29 (69.0) | 94 (45.9) |
| Month 12, n (%) | 17 (39.5) | 100 (48.5) |
| BL-M12 Δ change | Δ -29.5% | Δ +2.6% |
| *Missing* | *1 (BL)* | *2 (BL); 1 (M12)* |
| Felt sad, down or depressed | | |
| Baseline, n (%) | 21 (48.8) | 64 (31.4) |
| Month 12, n (%) | 14 (32.6) | 56 (27.1) |
| BL-M12 Δ change | Δ -16.2% | Δ -4.3% |
| *Missing* | | *3 (M12)* |
| Felt nervous or anxious | | |
| Baseline, n (%) | 16 (38.1) | 51 (25.0) |
| Month 12, n (%) | 12 (27.9) | 46 (22.3) |
| BL-M12 Δ change | Δ -10.2% | Δ -2.07% |
| *Missing* | *1 (BL)* | *3 (BL); 1 (M12)* |
| Skin problems, such as rash, dryness or itching | | |
| Baseline, n (%) | 20 (46.5) | 48 (23.5) |
| Month 12, n (%) | 8 (18.6) | 48 (23.5) |
| BL-M12 Δ change | Δ -27.9% | Δ -0.3% |
| *Missing* | | *3 (BL)* |
| **HIVTSQs** n** | - | **395** |
| Total score at baseline, median (Q1, Q3) | - | 56.0 (50.0, 60.0) |
| **HIVTSQc** n** | - | **223** |
| Total score at baseline, median (Q1, Q3) | - | 20.0 (4.0, 28.0) |

* Summary scores were normed to a mean of 50. Higher scores represent better QoL

** HIVTSQs and HIVTSQc were only evaluated in treatment experiences patients. The treatment satisfaction total score ranges from 0 to 60. The higher the score, the greater the satisfaction with treatment. The treatment satisfaction (change) total score ranges from -30 to 30. The higher the score, the greater the improvement in satisfaction with treatment; the lower the score, the greater the deterioration in satisfaction with treatment. A score of 0 represents no change.

### Antiretroviral therapy: resistance

### 029
### Paper-ID: 47497, O1

### Evolution of drug resistance mutation archive in patients infected with multi-class resistant HIV-1 and with viral suppression

Christian Hoffmann[1], Eva Wolf[2], Patrick Braun[3], Markus Bickel[4], Albrecht Stoehr[5], Silke Heldwein[6], Heribert Knechten[3], Stefan Esser[7], Ivanka Krznaric[8], Christoph Wyen[9], Markus Müller[10], Jürgen Brust[11], Jan-Christian Wasmuth[12], Martin Däumer[13]
*[1]ICH Hamburg; [2]MUC Research GmbH; [3]PZB Aachen; [4]Infektiologikum Frankfurt; [5]ifi-Institut für interdisziplinäre Medizin; [6]MVZ Karlsplatz; [7]University of Essen; [8]Praxis im Brauereiviertel; [9]Praxis am Ebertplatz; [10]Praxis Schwabstrasse 26; [11]Mannheimer Onkologie Praxis; [12]University of Bonn; [13]Institut für Immunologie und Genetik*

**Introduction:** Little is known about the evolution of the drug resistance mutation (DRM) archive in proviral DNA.
**Methods:** LOWER is a nation-wide study of patients with major DRMs (using Stanford-HIVdb v8.6.1) in $\geq$ 3 ARV classes (of NRTIs, NNRTIs, PIs, INSTIs). In this subanalysis, we selected subjects with viral suppression (VS, < 50 copies/mL) and a pronounced resistance patterns in proviral DNA assessed by deep sequencing (DS): Subjects in whom all historically known DRMs were detected in proviral DNA ("conservers") were compared with those who "had lost" all their DRMs ("discarders").

**Results:** Among a total of 195 patients with VS enrolled in LOWER, we identified 52 subjects with a pronounced resistance pattern in proviral DNA. There were 30 (15.4%) conservers, among them 16 subjects in whom all DRMs were re-detected at a Sanger cut-off of 15% (14 subjects had at least one DRM detected by DS only). There were also 22 (11.3%) discarders. Conservers and discarders did not differ in terms of age, gender, HIV duration or subtype, tropism, antiretroviral regimen, current or nadir CD4 cell count. Moreover, there were no differences with regard to time of viral suppression (7.4 years versus 7.1 years) and the time of previous virological failure (VF, 3.0 years versus 4.6 years). As shown in the Figure, conservers were found among those with short VF and long VS, while discarders were found among patients with long VF and short VS.

**Conclusion:** In patients with multiclass-resistant HIV-1 and with viral suppression, around 15% and 11% had conserved or lost all their DRMs in proviral DNA, respectively. In this cohort, conservers and discarders did not differ in terms of demographic factors, several HIV parameters but also with regard to time of virological suppression and of previous virological failure.

**030**
**Paper-ID: 47496, P1**

### Viral suppression in patients with multi-class resistance despite no fully active antiretroviral agent in the current regimen—a case series from the LOWER study

Christian Hoffmann[1], Eva Wolf[2], Patrick Braun[3], Peter Gute[4], Albrecht Stoehr[5], Silke Heldwein[6], Heribert Knechten[3], Stefan Esser[7], Ivanka Krznaric[8], Christoph Wyen[9], Markus Müller[10], Jürgen Brust[11], Jan-Christian Wasmuth[12], Martin Däumer[13]
*[1]ICH Hamburg; [2]MUC Research GmbH; [3]PZB Aachen; [4]Infektiologikum Frankfurt; [5]ifi-Institut für interdisziplinäre Medizin; [6]MVZ Karlsplatz; [7]University of Essen; [8]Praxis an der Kulturbrauerei; [9]Praxis am Ebertplatz; [10]Praxis Schwabstrasse 26; [11]Mannheimer Onkologie Praxis; [12]University of Bonn; [13]Institut für Immunologie und Genetik*

**Introduction:** Archived drug resistance-associated mutations (DRMs) may compromise virologic efficacy of ART. Current data indicate that resistance testing performed on HIV DNA lacks sensitivity compared with cumulative DRMs available from historical genotypic resistance testing (GRT) using plasma RNA. However, there are anecdotal reports on patients with long-term viral suppression (VS) despite extensive resistance to current ART.

**Methods:** LOWER is a nation-wide study of patients with major DRMs (using Stanford-HIVdb v8.6.1) in ≥ 3 ARV classes (NRTIs, NNRTIs, PIs, INSTIs). Here, we focussed on the mutational patterns in proviral DNA in the subgroup of patients who had achieved VS despite a low genotypic susceptibility score (GSS, < 1) of current ART regimen, indicated by historical GRT. Mutational patterns in proviral DNA using deep sequencing (DS) and APOBEC filtering were compared with cumulative DRMs from historical GRTs.

**Results:** Of 195 patients with VS enrolled in LOWER, we identified 7 patients with less than one fully active ARV, among them 3 subjects with a GSS = 0 according to historical GRTs. ARVs and GSS according to historical GRT and proviral DNA testing are shown in the Table. In 5/7 patients, VS had already lasted more than one year. All patients were treated with a PI-based regimen and none received an INSTI. Of note, applying the GSS score for proviral DRMs using a Sanger-like cut-off of 15%, 6/7 patients had at least 1 active antiretroviral drug in their regimen.

**Conclusion:** Durable viral suppression is possible in some patients with historical multi-class resistance, even when interpretation of cumulative historical DRMs suggests less than one (or even no) active agent in the current ART regimen. These findings indicate that at least some DRMs could have disappeared from the latent reservoir over time. In most patients, GSS was higher when resistance testing from proviral DNA was applied.

**031**
**Paper-ID: 46817, P2**

### Sustained viral suppression after switch to bictegravir/emtricitabine/tenofovir alafenamide (B/F/TAF) among clinical trial participants with preexisting M184V/I

Jürgen Rockstroh[1], Kristen Andreatta[2], Rima Acosta[2], Michelle L. D'Antoni[2], Danielle P. Porter[2], Silvia Chang[2], Ross Martin[2], Madeleine Willkom[2], Ian McNicholl[2], Joel Gallant[2], Hiba Graham[2], Sean Collins[2], Hal Martin[2], Kirsten L. White[2]
*[1]University Hospital Bonn; [2]Gilead Sciences Inc.*

**Introduction:** Preexisting resistance can affect antiretroviral therapy efficacy in people living with HIV (PLWH). One of the most common treatment-emergent resistance substitutions is M184V or, to a lesser extent, M184I. This substitution can be transmitted, archived in the viral reservoir, and reactivated, even after reversion to wild-type virus in plasma. Studies 1844, 1878, 4030, 4580, 4449, and 1474 demonstrated the safety and efficacy of switching stably suppressed PLWH to B/F/TAF. In this pooled analysis, we investigated the prevalence of preexisting M184V/I and impact on virologic outcomes.

**Methods:** Participants enrolled were aged ≥ 18 years (1844, 1878, 4030, and 4580), ≥ 65 years (4449), or 6 to < 18 years (1474). Preexisting drug resistance was assessed by historical genotypes and/or retrospective proviral DNA genotyping. Virologic outcomes were based on last available on treatment HIV-1 RNA, where early discontinuation with HIV-1 RNA < 50 copies/mL was considered suppressed.

**Results:** Altogether, 2034 participants switched to B/F/TAF, and cumulative baseline genotypic data were available for 90% (1824/2034). Preexisting M184V/I was detected in 10% (182/1824): by proviral genotyping only (79%), historical genotype only (10%), or both (11%). In 20% (37/182), M184V/I was the only resistance substitution detected, while in 80% (145/182), other primary resistance substitutions were detected in addition to M184V/I. At last study visit, 98% (179/182) of participants with preexisting M184V/I had HIV-1 RNA < 50 copies/mL compared to 99% (1623/1642) of those with wild-type M184 and 99% (2012/2034) of the overall B/F/TAF study population. No B/F/TAF-treated participant developed new drug resistance (Table).

**Conclusions:** Preexisting M184V/I was detected in 10% of suppressed participants' baseline genotypes, the majority of which was previously undocumented. High rates of virologic suppression in participants who switched to B/F/TAF, and the absence of treatment-emergent resistance, indicate B/F/TAF may be an effective and durable treatment for virologically suppressed PLWH with documented M184V/I.

| | P ooled B/F/TAF | Study 1844 | Study 1878 | Study 4030 | Study 4580 | Study 4449 | Study 1474 |
|---|---|---|---|---|---|---|---|
| Prior regimen | - | DTG/ABC/3TC | Boosted DRV or ATV + either FTC/TDF or ABC/3TC | DTG + either FTC/TDF or FTC/TAF | Any 3rd agent + 2 NRTIs | EVG/COBI/FTC/TAF or any 3rd agent + F/TDF | Any 3rd agent + 2 NRTIs |
| Total number of participants switched to B/F/TAF, n | 2034 | 545 | 532 | 283 | 489 | 85 | 100 |
| Median B/F/TAF treatment duration, weeks | 48 | 96 | 101 | 48 | 48 | 48 | 48 |
| HIV-1 RNA <50 c/mL at last visit, % (n/N) | 99% (2012/2034) | 98% (535/545) | 99% (525/532) | >99% (282/283) | 99% (486/489) | 100% (85/85) | 99% (99/100) |
| Baseline genotype available, % (n/N) | 90% (1824/2034) | 96% (522/545) | 94% (498/532) | 84% (237/283) | 98% (468/489) | 96% (82/85) | 17% (17/100) |
| Baseline M184V/I, % (n/N) | 10% (182/1824) | 3% (17/522) | 12% (62/498) | 20% (47/237) | 11% (50/468) | 4% (3/82) | 18% (3/17) |
| M184V/I HIV-1 RNA <50 c/mL at last visit, % (n/N) | 98% (179/182) | 100% (17/17) | 95% (59/62) | 100% (47/47) | 100% (50/50) | 100% (3/3) | 100% (3/3) |
| Treatment emergent resistance, n | 0 | 0 | 0 | 0 | 0 | 0 | 0 |

### 032
### Paper-ID: 47279, P3

### A case of virologic failure on B/F/TAF associated with the E138K integrase mutation

Hans-Jürgen Stellbrink, Michael Sabranski
*ICH Hamburg*

We report the case of an ART-naive patient with advanced HIV disease (CD4 4/µl, VL 1.500.000 copies/ml, MAI disease) and subtype B infection without baseline protease and RT resistance. Viral load was undetectable on TDF/FTC + DRV/r and following switch to TAF/FTC/DRV/c for 72 months. TAF/FTC/DRV/c was then switched to TAF/FTC/BIC. After 11 months low-level viremia developed (89, 140, 94, and 94 copies/ml), persisting over 6 months. Another 6 months following a single undetectable viral load, 500 copies/ml were reached. No RT or PI resistance was found, but integrase sequencing showed E138K, predicted to be associated with CAB, EVG, DTG, and BIC resistance by the ANRS algorithm, low-level EVG and RAL resistance by HIVDB 8.9.1, and low-level BIC and DTG resistance by HIV-GRADE, but no resistance by Rega 10.0.0. There was no indication of reduced adherence, co-infection or increased immune activation (HLA-DR + T-cells, C-RP).

One month after switch back to TAF/FTC/DRV/c undetectability was reached again. As a pretreatment plasma sample was unavailable, PBMC resistance analysis was performed, showing an E138EK mixed population.

**Conclusions:** E138K so far is known as an accessory mutation occurring in conjunction with key integrase resistance mutations. In this case its sole presence was associated with virological failure, matching the predictions of some but not all interpretation algorithms. Proviral DNA revealed archived E138K in a mixture with the wild-type. As the switch from DRV/c to BIC was the only change in the regimen we suggest that E138K was selected for during low-level replication on BIC, although its impact on sensitivity remains a matter of debate. The case challenges the perception that lowlevel viremia on TAF/FTC/BIC may be ignored in view of its high genetic barrier. It also points at the potential clinical relevance of the even higher genetic barrier of darunavir in some cases.

### 033
### Paper-ID: 46962, P4

### Characterization of HIV-1 drug resistance causing second-line combined antiretroviral therapy failure in Ethiopia

Andre Fuchs[1], Tafese Beyene Tufa[1], Hans Martin Orth[1], Nadine Lübke[2], Elena Knops[3], Eva Heger[3], Godana Jarso[4], Zewdu Hurissa[5], Dieter Häussinger[1], Tom Luedde[1], Björn-Erik Ole Jensen[1], Rolf Kaiser[3], Torsten Feldt[1]
*[1]Hirsch Institute of Tropical Medicine; [2]Universitätsklinik Düsseldorf; [3]Universitätsklinik Köln; [4]Adama Hospital Medical College; [5]Arsi University*

**Introduction:** As a consequence of the improved availability of combined antiretroviral therapy (cART) in resource limited countries, an emergence of HIV drug resistance (HIVDR) has been observed. Molecular testing for HIVDR is not routinely performed in Ethiopia before switching treatment regimens. This crosssectional study assessed the prevalence and spectrum of HIVDR in patients with failure of second line cART treated at the HIV-clinics of two central Ethiopian hospitals.

**Methods:** In April and May 2019, plasma samples from HIV-1-infected patients with second-line treatment failure (defined as HIV load $> 1000$ cop/mL $\geq 6$ months after initiation of second-line cART) treated in Adama and Asella (Ethiopia), were collected. Genotyping and geno2pheno-based resistance analysis were performed in Cologne, Germany.

**Results:** Overall, 37/714 (5.2%) of screened patients receiving second-line cART presented with treatment failure. At cART initiation, 62.2% (23/37) were WHO stage III and mean CD4 cell count was 173 (16,496) /µL. All patients were infected with HIV-1 subtype C. At study inclusion, mean HIV-1 load was 169,428 cop/mL. The most frequently used substances in first-line therapy were D4T, 3TC, TDF, EFV, NVP, and AZT. Common second-line cART regimes were TDF-3TC-ATV/r (54.1%) and AZT-3TC-ATV/r (27.0%) (Table 1). Successful resistance testing (35/37, 95.6%) revealed a predominance of the resistance mutations M184V (57.1%), Y188C (25.7%), M46I/L (25.7%), and V82A/M (25.7%). High-level resistance against the NNRTIs NVP (62.9%) and EFV (48.6%), and the NRTI 3TC (57.1%) was common. Susceptibility to EFV prevailed in only 28.6% of cases. High-level resistance against the PIs ATV (28.6%) and LPV (14.3%), and against TDF (17.1%) were less common. No viral strains transmitting INI-resistance were detected.

**Conclusions:** The prevalence of virus variants carrying resistance against the national standard first- and secondline cART components was high. Resistance testing is advisable before switching to second- or thirdline cART. More therapeutic options including integrase inhibitors should be made available.

| Table 1: cART history of participants with 2nd line treatment failure (n=37) | |
|---|---|
| **Initial 1st line cART regimen** | **n (%)** |
| D4T-3TC-NVP | 15 (40.5%) |
| TDF-3TC-EFV | 8 (21.6%) |
| AZT-3TC-NVP | 5 (13.5%) |
| D4T-3TC-EFV | 4 (10.8%) |
| AZT-3TC-EFV | 3 (8.1%) |
| TDF-3TC-NVP | 1 |
| AZT-3TC-LPV/r | 1 |
| **Failing 1st line regimen before switch to 2nd line** | |
| TDF-3TC-EFV | 11 (29.7%) |
| D4T-3TC-NVP | 7 (18.9%) |
| AZT-3TC-NVP | 7 (18.9%) |
| AZT-3TC-EFV | 5 (13.5%) |
| TDF-3TC-NVP | 3 (8.1%) |
| Other | 4 (10.8%) |
| **2nd line cART regimen at the time of sampling** | |
| TDF-3TC-ATV/r | 20 (54.1%) |
| AZT-3TC-ATV/r | 10 (27.0%) |
| ABC-3TC-ATV/r | 4 (10.8%) |
| ABC-3TC-LPV/r | 2 (5.4%) |
| AZT-3TC-LPV/r | 1 |

### Antiretroviral therapy: costs and safety

### 034
### Paper-ID: 46536, O1

### PLWHIV and prescribers in Germany: Left alone with the copayment of tenofovir-alafenamide/emtricitabine fixed-dose tablets. A problem and its extent

Matthias Stoll[1], Christian Kollan[2], Daniel Schmidt[2]
*[1]Medizinische Hochschule Hannover (MHH), Aidshilfe Niedersachsen, Landesverband e.V. (AHN); [2]Robert Koch Institut*

**Introduction:** According to the German Social Code (SGB-V), the Federal Joint Committee (GBA) can set reference prices for pharmaceuticals as an upper limit of reimbursement for people with statutory health insurance (SHI) (§35, SGB-V). For the first time in 10/2020, a reference price has been set for a patented antiretroviral fixed-dose-combination (FDC): tenofovir-alafenamide/emtricitabine (TAF/FTC = Descovy®). Gilead-Sciences, its exclusive manufacturer negotiated discount agreements with ~ 52% of the SHIs (as of 01.Nov.2020). Besides that, the price for Descovy® remained. This results in monthly co-payments of around €200 per patient for approximately half of those with SHI who receive Descovy®. Unfortunately, only few patients are able to bear this high level of co-payments. Therefore, physicians now have to consider the particular SHI to know who is affected by the copayment. As a solution for patients needing TAF, Gilead suggested switching to a single tablet regimen (STR) containing TAF/FTC that are not yet affected by reference prices.
**Methods:** We used German SHI prescription data, representing ~ 86% of nationwide prescriptions, to determine the share of Descovy®. In addition, data on the co-prescribed anchor substances of a complete ARTcombination will be analyzed.
**Results:** More than 10,000 patients/month received Descovy® in 2017, with a stable number of over 5,000 in 2020, corresponding to almost 10% of those with SHI [Fig. 1].
**Conclusions:** The joint prescription of Descovy® with efavirenz, raltegravir and other anchor substances not available as a TAF/FTC containing STR is particularly common. In 2020–2500 antiretrovirally treated persons with SHI were affected by the co-payment in Germany. High co-payments for Descovy® made treatment changes necessary for most of them. The poorly communicated co-payment obligation may have forced well-treated patients to switch, triggered avoidable side effects, gambled away trust and, in some cases without an adequate alternative, led to barely solvable conflicts.

Figure 1:
Patients receiving FTC/TAF as a Fixed-Dose-Tablet (Descovy®) in Germany [05/2016 to 06/2020]

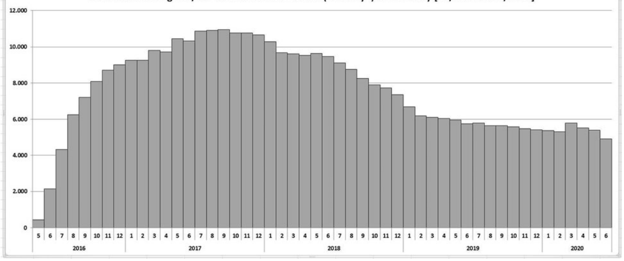

### 035
### Paper-ID: 47036, O2

### Metabolic and renal changes when switching to NRTI-free dolutegravir-containing 2 drug regimens—a sub-analysis of the DUALIS study

Malte Monin[1], Tim Kümmerle[2], Jochen Schneider[3], Christiane Cordes[4], Hans Heiken[5], Hans Jürgen Stellbrink[6], Ivanka Krznaric[7], Stefan Scholten[8], Björn Jensen[9], Heiko Jessen[10], Schulz Christian[11], Petra Spornraft-Ragaller[12], Pavel Khaykin[4], Eva Wolf[13], Christoph Spinner[3], Christoph Boesecke[1]
*[1]Bonn University Hospital; [2]Private Practice am Ebertplatz; [3]University Hospital Klinikum rechts der Isar; [4]Private Practice; [5]Private Practice Georgstraße; [6]ICH Study Center Hamburg; [7]Private Practice, Zentrum für Infektiologie, Prenzlauer Berg; [8]Private Practice Hohenstaufenring; [9] Heinrich Heine University Hospital Düsseldorf; [10]Private Practice Jessen + Kollegen; [11]University of Magdeburg; [12]Technical University of Dresden; [13]MUC Research, Clinical Research*

**Introduction:** Switching from 3 drug regimens (3DRs: boosted darunavir (bDRV)) and 2 nucleoside reverse transcriptase inhibitors (NRTIs), to 2DRs (bDRV and dolutegravir) was non-inferior to continuous 3DRs with regard to viral suppression in people living with HIV (PLWH) in the DUALIS study. This subanalysis focuses on changes in metabolic and renal parameters when sparing a NRTI backbone.
**Methods:** DUALIS was a randomized, open-label, multicenter (27) phase 3-trial. Participants were virally suppressed (HIV-RNA < 50 copies/mL) on 3DRs for at least 24 weeks. Data of metabolic and renal parameters at baseline and week 48 were compared.
**Results:** PLWH on 2DRs gained + 2.0 kg in body weight (− 0.2 to + 0.4) versus + 0.2 kg (− 1.9 to + 2.1) on 3DRs (p = 0.0006, see

Fig. 1). The BMI increased by + 0.6 kg/m$^2$ (− 0.1 to + 1.2 kg/m$^2$) and + 0.1 kg/m$^2$ (− 0.5 to + 0.7 kg/m$^2$), respectively (p = 0.0006). Total cholesterol increased by + 20.0 mg/dL (+ 3.0 to + 35.5 mg/dL) on 2DRs versus no increase (− 18.0 to + 15.5 mg/dL) on 3DRs (p < 0.001). The LDL-fraction increased by + 13.3 mg/dL (− 3.0 to + 31.3 mg/dL) and the HDL-fraction by + 4.9 mg/dL (− 1.0 to + 10.4 mg/dL) on 2DRs, whereas the LDL-fraction was stable (− 14.0 to + 18.0 mg/dL) and the HDL-fraction decreased by − 1.0 mg/dL (− 5.0 to + 4.0 mg/dL) on 3DRs (p < 0.001). The MDRD-eGFR decreased by − 7.8 mL/min/1.73m$^2$ (− 17.4 to − 0.3 mL/min/1.73m$^2$) on 2DRs versus − 0.4 mL/min/1.73m$^2$ (− 8.8 to + 5.7 mL/min/1.73m$^2$) on 3DRs (p = 0.0002). The Creatinine-CKD-EPI-eGFR decreased by − 8.0 mL/min/1.73m$^2$ (− 17.0 to − 0.6 mL/min/1.73m$^2$) on 2DRs versus –0.7 mL/min/1.73m$^2$ (− 9.4 to + 4.5 mL/min/1.73m$^2$) on 3DRs (p = 0.0002).

**Conclusion:** While being non-inferior regarding viral suppression, switching from 3 to 2DRs showed no advantages in metabolic or renal parameters over 48 weeks.

**Figure 1. Distribution of body weight changes at week 48 from baseline**

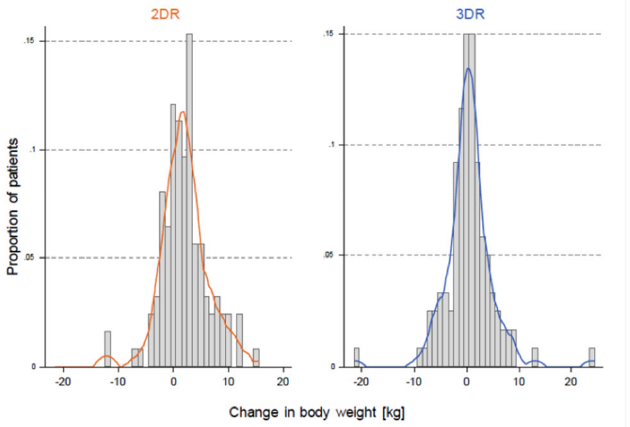

### 036
### Paper-ID: 47329, O3

### Weight gain and non-alcoholic fatty liver disease in HIV monoinfected patients - which impact have new generations of antiretroviral agents?

Jenny Bischoff[1], Carolynne Schwarze-Zander[1], Kathrin van Bremen[1], Christoph Boesecke[1], Jan-Christian Wasmuth[1], Jürgen K. Rockstroh[1], Jonel Trebicka[2]

*[1]Universitätsklinikum Bonn; [2]Universitätsklinikum Frankfurt am Main*

**Introduction:** While in the pre-ART era AIDS and underweight had shaped the fate of many people living with HIV, the introduction of antiretroviral therapy (ART) has brought about a change that goes far beyond the positive effects of weight normalization. Excessive weight gain and obesity pose new challenges for both, therapists and patients. Specifically, integrase strand transfer inhibitors (INSTIs) and tenofovir alafenamide/emtricitabine (TAF/FTC) have been discussed to contribute to a significant weight gain.

**Methods:** HIV monoinfected patients presenting at our outpatient clinic between August 2013 to December 2018 were enrolled. Liver stiffness and Hepatic steatosis (HS) were assessed annually by transient elastography using an M-probe of FibroScan. We compared weight gain and HS in patients being switched from TDF/FTC based treatment to TAF/F (n = 100), being administered an INSTI (n = 26) or both (n = 20) as well as in those patients remaining on TDF/FTC (n = 89) or INSTI based treatment (n = 31).

**Results:** 319 HIV+, mainly male (247/319; 77%) and Caucasian (243/319; 76·2%) were included. Overall, weight gain was 3·8 ± 3·8 kg (259) and HS increased significantly from 234·6 ± 57·4 dB/m (N = 240) to 253·7 ± 62·0 dB/m (N = 315). Weight increased significantly in those being switched from TDF/FTC to TAF/F from 76·5 ± 13·8 (N = 96) to 78·1 ± 17·7 (N = 92) and to 82·8 ± 24·7 (N = 97) after the switch. Distribution of HS grade S0-S3 changed towards a higher rate of S2 and S3 steatosis before and after switch (Fig. 1). Body weight remained stable in those individuals not being switched (TDF/FTC: 76·8 ± 13·7 (N = 85) vs. 78·7 ± 15·8 (N = 65); INSTI: 78·5 ± 10·6 (N = 29) to 80·0 ± 12·0 (N = 22)) Nevertheless, HS distribution changed also significantly in patients remaining on TDF/FTC (Fig. 1).

**Conclusion:** We observed several differences in HS progression and weight gain under different ART regimens and switches. Switching from TDF/FTC to TAF/F had a significant effect on HS distribution and weight gain, whereas remaining on TDF/FTC had just an effect on HS.

Figure 1: Hepatic steatosis distribution in HIV infected patients remaining on TDF/FTC and switching from TDF/FTC to TAF/F

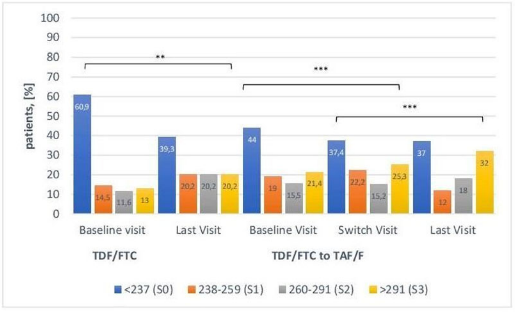

**p<0.01, *** p<0.001; CAP measurements [dB/m] by FibroScan®

### 037
### Paper-ID: 47499, O4

### Pharmacogenetics of dolutegravir and bictegravir neuropsychiatric adverse events

Christian Hoffmann[1], Alice MacKay[2], Carl Knud Schewe[3], Stefan Hansen[4], Thomas Buhk[4], Stefan Fenske[4], Hans-Jürgen Stellbrink[3], Axel Adam[4], Michael Sabranski[3], Marta Boffito[5], Andrew Owen[2], Emilie Elliot[2]

*[1]ICH Hamburg; [2]University of Liverpool; [3]ICH Study Center Hamburg; [4]ICH Study Center; [5]Chelsea and Westminster Hospital London*

**Introduction:** To date, no clear mechanism has been determined for the neuropsychiatric adverse events (NPAEs) associated with dolutegravir (DTG) and bictegravir (BIC). Pharmacogenetic variants of the OCT2 transmembrane transporter gene (SLC22A2) and UGT1A1 enzyme gene (UGT1A1*28) have been suggested for DTG. DTG and BIC are known substrates of the BCRP2 transporter on the blood brain barrier but variants in its coding gene (ABCG2) have yet to be investigated. This study examined the association between targeted transporter genetic variants and NPAEs leading to DTG and BIC drug cessation.

**Methods:** Cross-sectional, single centre pharmacogenetic study. Whole blood was collected from consented participants, who had either discontinued INSTIs secondary to NPAEs (cases) within their standard clinical care or who were randomly selected from a large pool of patients taking INSTIs with no side effects for at least 12 months (controls). Both groups completed drug toxicity questionnaires and clinical notes were reviewed retrospectively. ABCG2 421C > A (rs2231142) and SLC22A2 808C > A (rs316019) were genotyped using allelic discrimination assays and checked for Hardy–Weinberg equilibrium. Associations between genotypes, covariates and drug cessation were determined using univariate and multivariate linear regressions.
**Results:** 208 subjects were enrolled (189 males, 16 females, 3 transgender): 186 in the DTG analysis (42 cases) and 88 in the BIC analysis (21 cases; 66 participants had exposure to both drugs). 90% self described as Caucasian. Allele frequency for both genes mirrored European genotypic distributions. There was no difference in allelic distribution between cases and controls for either drug and either gene (individually or when combined). No significant association was seen with covariates.
**Conclusions:** There was no association between the ABCG2 and SLC22A2 genetic variants studied and NPAEs leading to DTG or BIC discontinuation in this cohort. The impact of genetic polymorphisms in transporters on neuropsychiatric tolerability of INSTIs in Caucasians appears limited.

### 038
### Paper-ID: 46952, P1

### HIV outcomes in Germany—beyond viral suppression

Georg Behrens[1], Katja Römer[2], Hendrik Streeck[3], Siegfried Schwarze[4], Sebastian Noe[5], Alexandra Wigger[6], Christian Thams[7], Jochen Jakobs[6], Christoph Spinner[8]
*[1]Medizinische Hochschule Hannover (MHH); [2]Gemeinschaftspraxis Gotenring; [3]Universitätsklinikum Bonn (AöR); [4]Projekt Information e.V.; [5]MVZ Karlsplatz; [6]ViiV Healthcare; [7]Gilead Sciences; [8]Technische Universität München*

**Introduction:** From the point of diagnosis, people living with HIV (PLHIV) must overcome significant challenges caused, among others, by public health policies and social reactions. Founded as a European initiative in 2016 (Link), HIV Outcomes has now been launched in Germany as an independent committee. The initiative not only aims to improve access to testing, treatment, innovative therapeutics and longterm care—it works towards greater awareness and demands political action going beyond viral suppression: long-term health outcomes and quality of life of PLHIV can only be achieved if treatment and care of PLHIV is viewed holistically and focused on the individual.
**Methods:** To support this approach, the initiative brings together key stakeholders: clinicians, academics, outpatient physicians, patient organizations and industry. Together they form the steering committee. HIV Outcomes aims to define the most pressing challenges for PLHIV in Germany from a community perspective and develop interdisciplinary proposals that can be implemented through political engagement.
**Results:** The committee defined the areas of action for 2021: Primarily, it will focus on parallels between HIV and COVID-19 and what lessons we can learn from them. Moreover, it will address the disruptive effect of COVID-19 on the health care system and how it affects the UNAIDS 2030 goals. It aims to tackle the reduction of discrimination and stigma, mental health problems, and focus on the opportunities of digitization in HIV care.
**Conclusion:** The initiative offers the steering committee an opportunity to identify and discuss the real challenges faced by PLHIV with representatives of politics and the healthcare system, thus providing food for thought for social and political debates. Being active on social media and conferences, like DÖAK 2021, would enable us to promote HIV Outcomes and establish a platform for exchange between patients, health care professionals and scientific as well as political community.

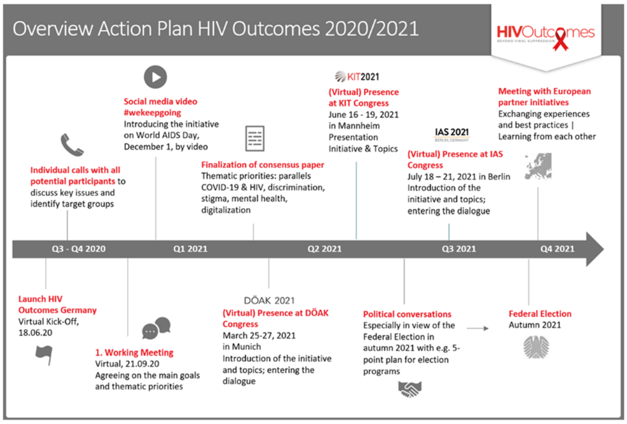

### 039
### Paper-ID: 46811, P2

### Clinical relevance of potential drug-drug interactions with bictegravir/emtricitabine/tenofovir alafenamide—real-world data from German IQVIA prescription database

Tim Umland[1], Hans-Jürgen Stellbrink[2], Marta Calvo[3], Astrid Zahn[3]
*[1]Alexander Apotheke; [2]Infektionsmedizinisches Centrum Hamburg (ICH); [3]Gilead Sciences GmbH*

**Introduction:** As people living with HIV (PLWH) age on antiretroviral treatment (ART), safe comedication is an increasing concern in everyday clinical practice. This analysis of longitudinal prescription information in PLWH in Germany focusses on the frequency of concomitant drugs and potential DDIs with ART in patients receiving B/F/TAF.
**Methods:** Data were obtained using the IMS® LRx database (IQVIA), which covered about 80% of prescriptions reimbursed by German statutory health insurance providers from 07/2018 to 06/2019. The study population consists of PLWH on continuous B/F/TAF for ≥ 3 months. Liverpool HIV Drug Interaction database was used to determine the potential DDIs between prescribed concomitant medications and B/F/TAF.
**Results:** Among 4,893 PLWH on B/F/TAF, 3,764 PLWH (77%) received ≥ 1 co-medication: 69% men, 13% women, 18% gender unknown; 59% aged 41–60 years; average number of co-medications was 4.0. Most commonly prescribed drugs classified by Anatomical Therapeutic Chemical level 3 (ATC3) were non-steroidal anti-rheumatic drugs (in 23% of patients on concomitant medications [N = 857]), antiulcerants (23%, N = 849), anti-depressants (15%, N = 566) and other analgesics/antipyretics (15%, N = 566) (Table 1). Potential relevant DDIs identified in ≥ 10 patients included metformin, betamethasone, dexamethasone, clarithromycin, itraconazole and verapamil. Contraindicated medications were used in < 0.25% of the cohort (Fig. 1). In ≥ 90% of PLWH receiving B/F/TAF, concomitant medications posed no or no relevant risk for interaction.
**Conclusions:** Overall, 77% of PLWH on B/F/TAF received ≥ 1 co-medication. Contraindicated medications were used in < 0.25% of the cohort. In those cases with potential relevant DDI, the individual medications can be replaced by other compounds of the same drug class member without potential interaction with B/F/TAF. Although this evaluation was limited by the exclusion of over-the counter drugs with potential for DDIs (e.g. mineral supplements or St. John´s wort), the overall potential for DDIs with B/F/TAF is low in clinical practice.

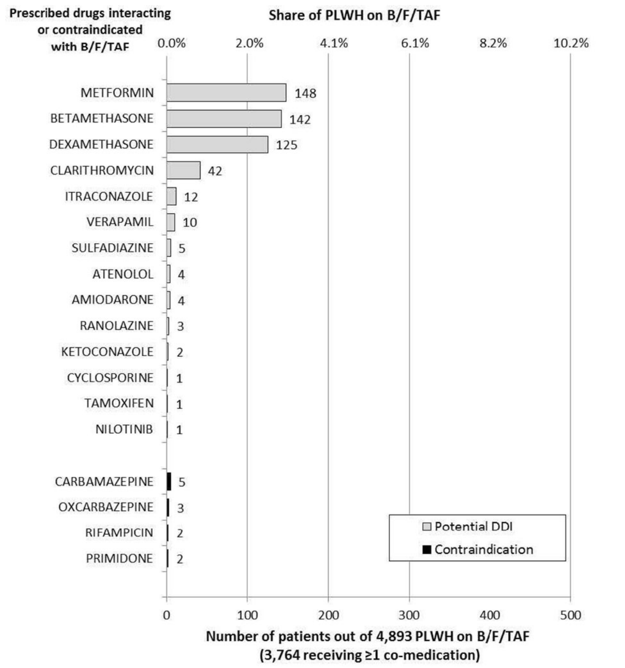

**Figure.** Prescribed drugs interacting with B/F/TAF and share of patients receiving respective drugs; *Potential DDI: exercise caution, close monitoring and dose adjustments may be required for certain patients*

### 040
### Paper-ID: 46965, P3

### Comparing 5-year body-mass-index gain of people living with HIV (PLH) with the general population

Laven Mavarani[1], Felix Maischack[2], Nico Reinsch[3], Sarah Albayrak-Rena[2], Franziska Kaiser[2], Anja Potthoff[4], Martin Hower[5], Sebastian Dolff[6], Norbert H. Brockmeyer[4], Dirk Schadendorf[2], Karl-Heinz Jöckel[1], Börge Schmidt[1], Stefan Esser[2]
*[1]Universitätsklinikum Essen, Institut für Medizinische Informatik, Biometrie und Epidemiologie (IMIBE); [2]Universitätsklinikum Essen, Klinik für Dermatologie und Venerologie, HPSTD Ambulanz; [3]Alfried Krupp Krankenhaus, Klinik für Kardiologie, Universität Witten/Herdecke; [4]Ruhr-Universität Bochum-Medizinische Fakultät, Klinik für Dermatologie, Venerologie und Allergologie; [5]Klinikum Dortmund, Abteilung für Pneumologie, Infektiologie und Innere Medizin; [6]Universitätsklinikum Essen, Klinik für Infektiologie*

**Introduction:** The prevalence of overweight and obesity has gained a steady increase worldwide within the last decades. It has been observed, that people living with HIV (PLH) under antiretroviral therapy (ART) may have an increased risk of gaining weight in adulthood. The present study aimed, whether PLH showed a higher body-mass-index (BMI) gain over 5 years of follow-up compared to the general population.
**Methods:** The HIV HEART (HIVH) study is a prospective cohort to assess cardiovascular risk in PLH. Descriptive statistics for baseline and 5-year-follow-up characteristics were calculated for HIVH and the population-based Heinz Nixdorf Recall Study (HNR) (aged 45–75 years). To adjust for the age- and sex-differences between study samples, age- and sex-adjusted linear regression models were calculated to assess the effect of HIV on BMI at baseline and percentage change in BMI after 5 years.
**Results:** Baseline characteristics of HIVH- and HNR-participants (N = 437 and N = 4126, respectively) are shown in Table 1. At baseline, 83% of the HIVH-participants are under ART. After 5 years of follow-up, 99% of HIVH is under ART treatment. Sex- and age-adjusted results of linear regression models showed 3.1 units lower baseline BMI in HIVH participants compared to HNR ($p < 0.0001$). After 5 years of follow-up, the percentage change in BMI was by 0.49% higher in HIVH participants compared to HNR ($p = 0.17$). Among the HIVH participants with and without ART at baseline, no differences in weight change were observed.
**Conclusions:** While PLH showed on average a lower BMI than the general population, BMI gain after 5 years was slightly higher in PLH. This weight gain in PLH might be associated with HIV specific risk factors, such as type of therapy and lifestyle, but could also be a result of coming-back-to-normal effects by ART.

*Table 1: Baseline and 5-year-follow-up characteristics of HIVH and HNR*

| | | HIVH | | HNR | |
|---|---|---|---|---|---|
| | | N | n(%) / MEAN ± SD | N | n(%) / MEAN ± SD |
| Baseline | | | | | |
| sex | female | 437 | 51 (11.7 %) | 4126 | 2090 (50.7 %) |
| age | [years] | 437 | 52.8 ± 6.6 | 4126 | 59 ± 7.7 |
| blood pressure | systolic [mmHg] | 437 | 137.9 ± 22.0 | 4121 | 132.4 ± 20.6 |
| | diastolic [mmHg] | 437 | 85.5 ± 12.1 | 4121 | 81.3 ± 10.8 |
| diabetes mellitus | yes | 437 | 29 (6.6 %) | 4126 | 510 (12.4 %) |
| total cholesterol | [mg/dl] | 425 | 223.6 ± 49.3 | 4111 | 229.2 ± 38.8 |
| LDL | [mg/dl] | 170 | 130.7 ± 39.9 | 4099 | 145.4 ± 36.0 |
| HDL | [mg/dl] | 391 | 48.4 ± 15.7 | 4109 | 58.5 ± 17.2 |
| weight | [kg] | 437 | 76.2 ± 13.2 | 4126 | 79.1 ± 15.0 |
| BMI | [kg/m²] | 437 | 24.6 ± 3.7 | 4126 | 27.8 ± 4.5 |
| waist circumference | [cm] | 173 | 91.8 ± 11.5 | 4124 | 93.8 ± 13.1 |
| hip circumference | [cm] | 173 | 93.1 ± 9.7 | 4125 | 103.0 ± 9.9 |
| waist-hip ratio | | 173 | 0.99 ± 0.07 | 4124 | 0.91 ± 0.09 |
| 5-Year Follow Up | | | | | |
| sex | female | 437 | 51 (11.7 %) | 4126 | 2090 (50.7 %) |
| age | [years] | 437 | 58.0 ± 6.6 | 4126 | 64 ± 7.6 |
| blood pressure | systolic [mmHg] | 437 | 139.6 ± 20.4 | 4116 | 134.1 ± 19.9 |
| | diastolic [mmHg] | 437 | 84.3 ± 11.5 | 4116 | 79.1 ± 10.5 |
| diabetes mellitus | yes | 437 | 55 (12.6 %) | 4126 | 771 (18.7 %) |
| total cholesterol | [mg/dl] | 431 | 220.1 ± 47.2 | 4098 | 225.2 ± 41.3 |
| LDL | [mg/dl] | 403 | 136.9 ± 38.6 | 4096 | 131.1 ± 34.8 |
| HDL | [mg/dl] | 405 | 49.5 ± 17.4 | 4096 | 60.1 ± 16.3 |
| weight | [kg] | 437 | 77.6 ± 14.4 | 4126 | 79.8 ± 15.5 |
| BMI | [kg/m²] | 437 | 25.1 ± 4.3 | 4126 | 28.2 ± 4.8 |
| waist circumference | [cm] | 400 | 93.8 ± 11.0 | 4126 | 96.0 ± 13.4 |
| hip circumference | [cm] | 400 | 93.1 ± 10.2 | 4126 | 103.3 ± 9.7 |
| waist-hip ratio | | 400 | 1.01 ± 0.07 | 4126 | 0.93 ± 0.09 |

### 041
### Paper-ID: 47262, P4

### Weight change in elderly people living with HIV

Sebastian Noe[1], Eva Wolf[2], Christian Hoffmann[3], Knud Schewe[3], Robert Baumann[4], Franz Mosthaf[5], Axel Baumgarten[6], Markus Müller[7], Nils Postel[8], Annamaria Balogh[2], Anna Ivanova[9], Nikola Hanhoff[10], Hans Jäger[1]
*[1]MVZ München am Goetheplatz; [2]MUC Research GmbH; [3]ICH Study Center Hamburg; [4]Praxis Dr. Baumann; [5]Gemeinschaftspraxis für Hämatologie, Onkologie und Infektiologie; [6]Zentrum für Infektiologie Berlin/Prenzelberg; [7]Gemeinschaftspraxis Schwabstraße 26; [8]Prinzmed; [9]I-Biostat; [10]Deutsche Arbeitsgemeinschaft niedergelassener Ärzte in der Versorgung HIV-Infizierter e.V. (dagnä)*

**Introduction:** Weight gain in people living with HIV (PLWH) and without HIV is of increasing interest. However, there is few and contradictory comparative data.
**Methods:** Post-hoc analysis of the longitudinal multicenter 50/2010 study, including PLWH underART, people with type 2 diabetes

(PT2D), or neither (controls) of ≥ 50 years of age between 2009 and 2011in Germany. General linear models were fit evaluating associations of covariates (group, age, sex, obesity [≥ 30 $kg/m^2$], HADS score [≥ 8], regular alcohol consumption, having a partner, ART class) with weight change.

**Result:** 615 subjects were included (487 [79.2%] men, 61.3 [SD 8.2] years mean age), i.e. 214 (34.8%) PLWH, 203 (33%) PT2D, and 198 (32.2%) controls. Mean weight change over 18 months was 0.1 kg (SD6.6) (p = 0.779), with 0.2 kg (SD 5.7) (p = 0.560) for PLWH, -0.2 kg (SD 8.1) (p = 0.670) for PT2D, and0.2 kg (SD 5.8) (p = 0.570) for controls (p = 0.995 between groups). Being obese (β = − 2.3, p < 0.001), being smoker (β = − 1.5, p = 0.02), and ≤ 60 years old (β = 1.1, p = 0.04) were associated with weight change in the entire study sample. Among PLWH, the final multivariate model on weight change over 18 months included being obese (β = − 6.5, p < 0.001), having a partner (β = 2, p = 0.012), being ≤ 60 years old (β = − 1.2, p = 0.112), regular alcohol consumption (β = 1.2, p = 0.18), and CD4 cells (log) (β = − 1.9, p = 0.005), as well as an interaction term for being obese and having a partner (β = 4.7, p = 0.195).

**Conclusion:** In the absence of potentially (excessive) weight promoting ARVs (including 2nd generation integrase inhibitors and tenofovir alafenamide), weight change for elderly people living with and without HIV seems to be comparable. In the overall study sample, being obese, or smoker were negatively, being ≤ 60 years of age was positively associated with weight change. Within the subgroup of PLWH, being obese, a smoker, ≤ 60 years old, and CD4 cells were negatively associated, while having a partner and alcohol consumption were positively associated with weight change.

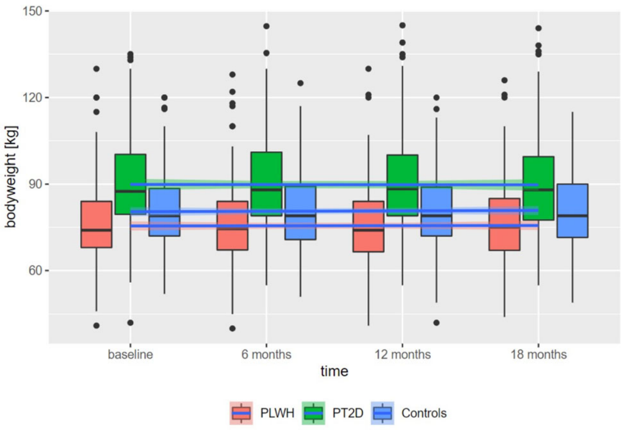

### 042
### Paper-ID: 47227, P5

### Adherence in the RADATA-cohort: correlation with age, gender and death in HIV-positive patients

Paul Plettenberg[1], Frieder Kuhlendahl[2], Thore Lorenzen[3], Albrecht Stoehr[2], Peter Buggisch[2], Jörg Petersen[2], Andreas Plettenberg[2]
*[1]Asklepios Medical School Hamburg; [2]ifi-Institut für interdisziplinäre Medizin; [3]DammtorPraxis*

**Introduction:** The expert advice system RADATA offers physicians advice for antiretroviral therapy. Clinical data including adherence were documented in a cohort. The importance of adherence for the success of ART is generally acknowledged. Still opinions differ on the impact of adherence on survival of HIVpositive patients. One difficulty in adherence-research is the method of inquiry. Self-reported adherence is typically associated with higher adherence results due to the bias to give more socially agreeable answers to the physician.

**Methods:** During an 18-year period, 1234 HIV-positive patients were included into the RADATA-system. By inclusion patients were given an adherence questionnaire. This was completed by 932 patients and afterwards sent to the experts. To ensure a truthful adherence report, patients were informed that the data were not given to their attending physician. Adherence > 90% was assumed if intake > 90% of the ART drugs was stated for the previous week. It was evaluated whether factors such as gender, age and death correlated with adherence > 90%.

**Results:** Information concerning death/survival is known for 703/932 patients. 608/703 patients (86.5%) reported adherence > 90%, 95 patients (13.5%) < 90%. 88.0% of 565 male and 80,4% of 138 female patients stated adherence > 90% (p = 0.02). Patients with adherence > 90% were older than patients with adherence < 90% (mean age 42 vs. 46 years, p = 0.002). 49/608 (8.1%) patients with adherence > 90% and 14/95 patients (14.7%) with adherence < 90% had died within the observation period (p = 0.034).

**Conclusion:** In this long-term cohort adherence > 90% correlates significantly with improved survival. Adherence > 90% was found more frequently in elderly patients and in male gender. The data show the importance of adherence to ART for prognosis of HIV-infection. This emphasizes the importance of adherence evaluation combined with measures for improvement of adherence in the management of HIV-infection.

| | | | adherence | | total |
|---|---|---|---|---|---|
| | | | >90% | <90% | |
| patients | alive | n (%) | 559 (91,9%) | 81 (85,3%) | 640 (91,0%) |
| | dead | n (%) | 49 (8,1%) | 14 (14,7%) | 63 (9,0%) |
| total | | n (%) | 608 (100,0%) | 95 (100,0%) | 703 (100,0%) |

### Oncology

### 043
### Paper-ID: 46955, O1

### Efficacy of trichloroacetic acid vs. electrocautery for the treatment of anal intraepithelial neoplasia marker lesions in HIV-positive patients

Stefan Esser[1], Alexander Kreuter[2], Anja Potthoff[3], Mark Oette[4], Konstantinos Bilbilis[5], Hildegard Lax[5], Heidi Wiehler[1], Aylin Yavuz[1], Norbert H. Brockmeyer[3], Karl-Heinz Jöckel[5], Dirk Schadendorf[1], Ulrike Wieland[6]
*[1]Universitätsklinikum Essen, Klinik für Dermatologie und Venerologie, HPSTD Ambulanz; [2]Helios St. Elisabeth Klinik Oberhausen, Klinik für Dermatologie, Venerologie und Allergologie, Universität Witten/Herdecke; [3]Ruhr-Universität Bochum-Medizinische Fakultät, Klinik für Dermatologie, Venerologie und Allergologie; [4]Krankenhaus der Augustinerinnen, Köln; [5]Universitätsklinikum Essen, Institut für Medizinische Informatik, Biometrie und Epidemiologie (IMIBE); [6]Universitätsklinikum Köln, Institut für Virologie*

**Background:** Anal cancer (AC) is increasing in HIV-positive patients (HIV+). Screening and treatment of anal intraepithelial neoplasia (AIN) as potential AC precursor are recommended in guidelines for HIV+. Current treatment options are suboptimal due to high relapse rates. Furthermore, data on AIN treatments from prospective trials are limited. Surgical and ablative treatments are expensive and complex in routine clinical care. We compared efficacy of electrocautery (ECA) as standard of care vs. topical trichloroacetic acid (TCA, 85%) for treatment of AIN.

**Methods:** The TECAIN Study is a 1:1 randomized, unblinded, multicenter, non-inferiority trial investigating efficacy and safety of ECA vs. TCA for treatment of AIN, evaluated by high-resolution anoscopy (HRA) and targeted biopsies of HPV-associated lesions. HIV+ with histopathologically confirmed AIN were recruited from HIV-outpatient clinics specialised in proctologic care. The primary efficacy endpoint was therapeutic success defined as clinically and histologically confirmed resolution (or regression) of AIN marker lesions (ML), chosen by the investigator at baseline visit, 4 weeks after the last treatment of maximal 4 interventions every 4 weeks over 16 weeks since randomization.

**Results:** 180 HIV+ (98% males, 81% MSM, mean age 44.6 ± 10.7 SD years) with complete data and 196 ML (38% AIN I, 62% high grade AIN (HGAIN)) were preliminary evaluated. 100 ML were treated with ECA and 96 with TCA. Outcome analysis showed a protocol defined treatment success in 67 (67%) ML of the ECA vs. 55 (57%) ML of TCA group (p = 0.16). After 171 ECA interventions (1.7 ECA/ML) 14 (14%) of the marker lesions showed a clinical and 27 (27%) a histological treatment failure vs. 22 (23%) clinical and 28 (29%) histological treatment failures after 170 TCA interventions (1.8 TCA/ML) (p = 0.10 clinical vs p = 0.73 histological treatment failure).

**Conclusion:** Efficacy of the treatment of AIN in HIV+ was not significantly different between ECA and TCA.

### 044
### Paper-ID: 46922, O2

### Hyperinflammation and aggressive lymphomas: observations from a single centre retrospective cohort study in Berlin, Germany

Pascal Migaud[1], Kai Hosmann[1], Keikawus Arastéh[1], Michael Rittweger[2], Hartmut Stocker[1], Markus Müller[1]
*[1]St.Joseph Krankenhaus Berlin-Tempelhof; [2]Praxis Wimersdorfer*

**Introduction:** In high-income countries lymphoproliferative diseases are the most common cause of death among people living with HIV (PLHIV). The mortality rate of lymphoproliferative diseases as a group appears to be higher in PLHIV as compared to HIV-negative patients. This may be due to a high rate of rare, aggressive lymphomas such as HHV-8-associated primary effusion lymphoma (PEL), plasmablastic lymphoma (PBL) and Burkitt lymphoma (BL) as well as a high incidence of associated hemophagocytic lymphohistiocytosis (HLH), a life-threatening hyperinflammatory syndrome that emerges on the basis of a dysregulated immune system. We describe the frequency of individual lymphoproliferative disease entities and the incidence of hyperinflammation among the entire cohort.

**Methods:** Retrospective cohort study comprising all adult HIV-infected patients with biopsy or cytology proven lymphomas that were treated by our team between October 2013 and July 2019. All patients were included in the German ARL cohort study.

**Results:** 75 patients (four female) were included in this analysis. The median age at diagnosis of malignant lymphoma was 50 (range 22–70) years. 32 (42%) patients presented with Hodgkin lymphoma. DLCBL, PBL, PEL and BL were diagnosed in 24 (32%), 8 (10%), 6 (8%) and 5 (7%) cases, respectively. After a median observation period of 23.5 months (range 0–74 months), the 1- and 2 year overall survival was 75% and 68%. At univariate analysis CD4(+) T-cell counts below 100/µl, nonsuppressed viral load and ART-naivety at the time of diagnosis were significantly associated with lower overall survival rates with p-values of 0.0023, 0.006 and 0.001, respectively. Six patients developed hyperinflammation in the course of their treatment/disease with positive HLH-Score 2004 and H-Score Saint-Antoine. HLH/Hyperinflammation was associated with lower overall survival (p = 0.005).

**Conclusions:** Physicians treating patients with HIV-related lymphomas should be aware of the possibility of hyperinflammation, especially in HHV-8 associated lymphomas and Morbus Hodgkin.

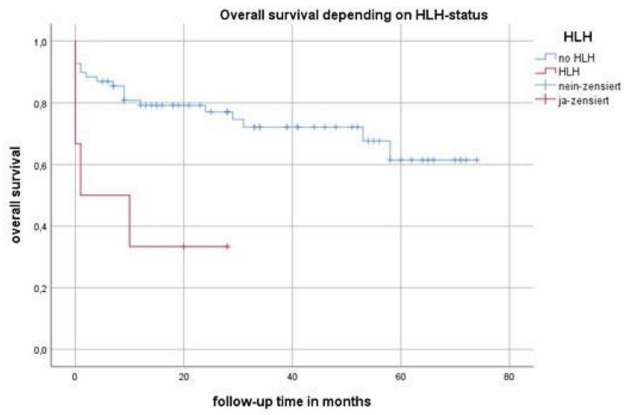

### 045
### Paper-ID: 47707, O3

### Treatment and outcome of men with HIV-associated germ cell tumors

Marcus Hentrich[1], Mark Bower[2], Gedske Daugaard[3], Annette Dieing[4], Markus Bickel[5], Massimiliano Berretta[6], Florian Lesmeister[1], Vindi Jurinovic[7], Albrecht Stöhr[8], Julia Heinzelbecker[9], Ivanka Krznaric[10], Klaus-Peter Dieckmann[11], Andrea Necchi[12], Pablo Maroto Rey[13], Jürgen Rockstroh[14], David Pfister[15], Margarida Brito[16], Burkhard Otremba[17], Christian Hoffmann[18]
*[1]Red Cross Hospital, University of Munich, Germany; [2]National Centre for HIV Malignancy, Chelsea & Westminster Hospital, London, UK; [3]Rigshospitalet, Copenhagen, Denmark; [4]Vivantes Klinikum am Urban, Berlin, Germany; [5]Infektiologikum, Frankfurt, Germany; [6]National Cancer Institute, Aviano, Italy; [7]University of Munich, Germany; [8]Institute for Interdisciplinary Medicine, Hamburg, Germany; [9]University of Homburg, Germany; [10]Center for Infectious Diseases, Berlin, Germany; [11]Asklepios Klinik Altona, Hamburg, Germany; [12]Fondazione IRCCS Istituto Nazionale dei Tumori, Milan, Italy; [13]Hospital de la Santa Creu i Sant Pau, Barcelona, Spain; [14]University of Bonn, Germany; [15]University of Cologne, Germany; [16]Instituto Português de Oncologia Francisco Gentil, Lissabon, Portugal; [17]Oncology Oldenburg, Germany; [18]IPM Study Center, Hamburg, Germany*

**Introduction:** Previous studies showed that men with HIV-associated germ cell tumors (HIV-GCT) have an inferior overall survival (OS) compared with their HIV-negative counterparts. Little data is available on treatment and outcome of HIV-GCT in the era of combined antiretroviral therapy (cART).

**Methods:** Men living with HIV aged ≥ 18 years (yrs) with a diagnosis of histologically proven GCT made from 01/1996 to 07/2018 were included in this retrospective international study. Primary outcomes were OS and progression-free survival (PFS).

**Results:** Data of 89 men from 23 institutions and 6 countries with a total of 92 HIV-GCC (2 synchronous and 1 metachronous bilateral GCT) were analysed, among them 64 (70%) seminomas and 28 (30%) nonseminomas. Median age was 36 yrs (range 22–52) and median time from HIV to GCT diagnosis was 5 yrs (range 0–29). Median CD4 count at GCT diagnosis was 420 cells/µl (range 3–1503) and 83% of pts were on cART. 44/80 (55%) patients with gonadal GCT had stage I, of which 22 (50%) were followed by active surveillance, and 11 (25%) received adjuvant chemotherapy (CT) or radiotherapy (RT). 39/46 patients with stage II/III GCT disease received CT and 6 (stage II seminoma) RT, and 1 nonseminoma patient underwent surgery. Antineoplastic treatment resulted in a fall of CD4-counts from median 353 to 285/µl in pts without cART while CD4-counts remained stable in pts on cART. Overall, 12/89 (13%) pts have died. Causes of death were refractory GCT (n = 5), an AIDS-defining illness (n = 3) and other (n = 4). After a median follow-up of 6.5 yrs (range, 0.3–20.9), the 5- and 10-year PFS rate was 81% and 73%, and the 5- and 10-year OS rate was 91% and 85%, respectively.
**Conclusion:** The 5- and 10-year PFS and OS rates of men with HIV-GCT are similar to those reported for HIV-negative GCC.

### AIDS, neurology and other clinical issues

### 046
### Paper-ID: 46882, O1

### Incidence of milder forms of HIV-associated neurological disorders (HAND) in the cART era

Gabriele Arendt, Florian Dimmers, Eser Orhan
*Neuro-Centrum Düsseldorf*

**Introduction:** After the introduction of highly active antiretroviral therapy (HAART) in the treatment of HIV-positive patients in 1996, incidence and prevalence of HIV-associated neurological disorders (HAND) declined until about 2002. At the beginning of the new century, prevalence of milder neurocognitve deficits (asymptomatic neurocognitive impairment = ANI and mild neurocognitive deficits = MNCD) rose again, presumely, because of the patients' longer survival times. The next marked step forward in therapy was the market introduction of the integrase strand inhibitors in 2007, which do not only effectively suppress viral load in blood, but do this also very rapidly. From this moment on, treatment was called "cART" (combination antiretroviral treatment). Finally, the opening of the START study (strategic timing of antiretroviral therapy) in 2015 provoked that every patient being identified as HIV+ , was offered treatment independent from CD4+ -cell count or plasma viral load., which ended up in very early individual treatment starts. In this study, we examined the effects of the new treatment procedures on the manifestation of neurocognitive deficits.
**Methods:** We analysed data of a prospective observative cohort study comprising almost 6000 patients recruited consecutively over three decades. They underwent neuropsychological testing according to the "Frascat"i-criteria (three step diagnostic nomenclature). We compared patients recruited from 2008-12-2019 with those recruited from 1996 to 2007.
**Results:** Incidence of ANI significantly declined from 12.13% in 2008 to 9.53% in 2019. MNCD and HIVassociated dementia incidence remained uninfluenced.
**Conclusions:** Obviously, the earliest and mildest form of HAND (ANI) can be prevented in the cART era, which is not true for MNCD and HIV-associated dementia. This underlines the effectivity of modern, often integrase strand inhibitors containing antiretroviral regimen and the advantages of an early treatment start.

### 047
### Paper-ID: 47047, O2

### Vaccination coverage among people living with HIV in Germany according to EACS recommendations

Sven Breitschwerdt, Carolynne Schwarze-Zander, Ahmad Al Tayy; Julia Mutevelli, Jan-Christian Wasmuth, Jürgen Rockstroh, Christoph Boesecke
*Universitätsklinik Bonn*

**Introduction:** In people living with HIV (PLWH) EACS guidelines recommend vaccinations against HAV, HBV, HPV, Influenza, Neisseria meningitidis, Streptococcus pneumoniae and VZV. Data on realization in clinical care are tenuous. Here we present an analysis on vaccination rates in an HIV outpatient clinic population at a German tertiary care center.
**Methods:** Using a questionnaire as well as the clinical database, all PLWH presenting to the HIV outpatient clinic of the University Hospital in Bonn, Germany between April and June 2018 had been screened for vaccination status with regard to Pneumococcus, Hepatitis A and B, seasonal Influenza, Varicella, Meningococcus and HPV.
**Results:** Overall, 305 PLWH were included (82.3% male, 17.7% female), the median age was 48 years (IQR 47–51). In 172 (56.4%) the CD4 T cell count was $\geq$ 200/µl, 65 (21.3%) were diagnosed at CDC C3, while for 289 (94.8%) virus load was undetectable. 270 participants (88.5%) had an immunization card at presentation, with no difference between the genders. The highest rates could be observed for vaccination against HAV (87.40%), followed by Influenza (75.70%) and Streptococcus pneumoniae (77.38%). Coverage of vaccinations against pneumococcus was high in PLWH > 65 years (79.17%), exceeding the rates among HIV-negative population. Influenza vaccination coverage was 75.70%, among > 60 years it was 83.33% with no difference between the genders, again higher compared to the HIV-negative population.
**Conclusions:** Vaccination rates among PLWH seem to be higher compared to general population, in particular for Influenza and Pneumococcus. However, as the EACS guidelines are still not fully implemented into daily routine with regard to other vaccinations, the need of vaccinations among health care professionals and PLWH needs to be emphasized.

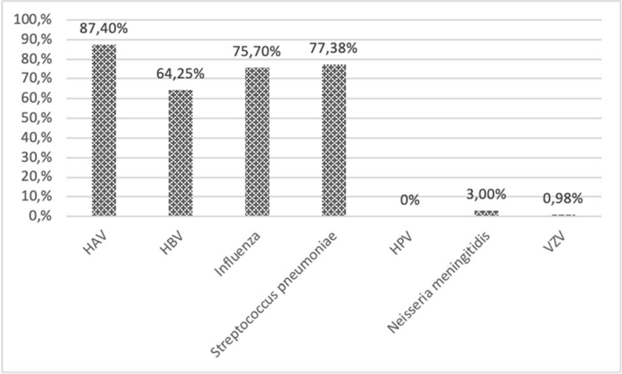

### 048
### Paper-ID: 46966, P1

### Systemic and neuro-efficiency of antiretroviral combinations in the cART in comparison with the HAART-era

Gabriele Arendt, Melanie Dickhoff, Eser Orhan
*Neuro-Centrum Düsseldorf*

**Introduction:** After the introduction of highly acitve antiretroviral therapy (HAART) into the treatment of HIVpositive patients in 1996 incidence and prevalence of virus-associated brain disease declined at first and after a couple of years continuously rose again. During these years, a three-step diagnostic nomenclature, the so-called "Frascati-criteria" was established (asymptomatic neurocognitive impairment = ANI, mild neurocognitive deficit = MNCD and HIV-associated dementia = HAD). After the introduction of an additional new pharmacological group, the integrase inhibitors = INI in 2007, treatment was named "cART" (combined antiretroviral therapy). In this study, the influence of cART on the systemic HIV-infection surrogate markers (CD4+ -cell count and plasma viral load) in parallel to the manifestation of neurocognitive deficits (results of neuropschological tests) in comparison with the HAART-era was analysed.
**Methods:** Three periods were analysed: 1996–2007 (HAART), 2007–2015 (cART) and 2015-06-30-2020 (period after the opening of the START study). According to the "Frascati-criteria", five neuropsychological tests were evaluated and correlated with the systemic surrogate markers of the infection.
**Results:** Suppression of plasma viral load became much more effective after 2007. In parallel, neuropsychological test results improved, especially after 2015, the period with an early therapy start.
**Conclusion:** The cART-era definitely initiated therapeutic improvement, especially early treatment start seems to play a positive role.

### 049
### Paper-ID: 46880, P2

### Frequent therapy changes in HIV-positive patients and their significance for the central nervous system (CNS)

Gabriele Arendt[1], Niki Kuzmicic[2], Eser Orhan[1]
[1]*Neuro-Centrum Düsseldorf;* [2]*neuro-Centrum Düsseldorf*

**Introduction:** Since 2007 antiretroviral therapy is called "cART" (combination antiretroviral therapy) because of the different pharmacological groups used in fixed combinations. Treatment has to be adapted during the course of the infection for different reasons, first because of virological failure, second because of side effects and third because of the doctors‘ wish to treat his/her patients with the best (newest) therapy option.
**Methods:** In a retrospective analysis, neuropsychological test results of 1301 HIV-positive patients with one or two treatment changes (group A) were compared with those of 508 patients with 3 or more changes (group B).
**Results:** Group B patients performed significantly worse in all tests applied, especially in motor, but also in neurocognitive tests (Trail-Making Tests form 1 + 2, Digit-Symbol-, Grooved-Pegboard- and Stroop Colour Test, AIDS-Dementia- Scale as well as in verbal fluency tests. These results were additionally influenced by a time factor; the earlier during the course of the infection treatment combinations were changed, the worse were the test results.
**Conclusion:** With respect to the brain, treatment changes should only be done when absolutely necessary.

### 050
### Paper-ID: 46975, P3

### Width of the third ventricle in HIV patients: correlation with neuropsychological deficits

Matthias Heit[1], Dominic Kaddu-Mulindwa[1], Gudrun Wagenpfeil[2], Stefanie Behnke[3], Mathias Fousse[1]
[1]*Universitätsklinik des Saarlandes;* [2]*Universität des Saarlandes, Institut für medizinische Biometrie, Homburg, Deutschland;* [3]*Knappschaftskrankenhaus Sulzbach (Saar)*

**Introduction:** Due to antiretroviral therapy (ART) with nearly normal life expectancy other comorbidities like neuropsychological deficits compromising the quality of life (qoL) in people living with HIV (PLWH). Previous studies showed a correlation between the width of the third ventricle (WTV) and neurocognitive disorders.
**Methods:** We investigated the correlation of neuropsychological disorders and HIV specific characteristics with WTV as a brain atrophy marker using transcranial sonography. We used Becks Depression Inventory (BDI) for depression screening, the questionnaires Fatigue Severity Scale (FSS) for fatigue and ShortForm-36 (SF36) for qoL and Consortium to establish a registry for Alzheimer's disease (CERAD-PLUS) as neuropsychological test battery.
**Results:** 52 PLWH (47 males) and 28 non-infected controls (23 males) with mean age of 50 years were examined. WTV correlated significantly with age ($r = 0.546$; $p < 0.01$). WTV was larger in PLWH (mean = 4088 mm) compared to controls (mean = 3288 mm) ($p = 0.085$). There was no correlation between duration of HIV-infection or CD4-nadir with WTV. PLWH had both significantly higher BDI-Score ($p = 0.005$) and FSS-Score ($p = 0.012$). Controls performed better in most terms of qoL (SF-36), with significant difference regarding the items "social functioning" ($p = 0.019$), "general mental health" ($p = 0.001$), "role limitations due to emotional" ($p = 0.018$), "vitality, energy or fatigue" ($p = 0.028$) and "general health perceptions" ($p = 0.012$). Controls showed slightly better performance of CERADPLUS with significant difference in neurocognitive subtest "semantic verbal fluency" ($p = 0.023$) and "Trail Making Test A" ($p = 0.030$). Nevertheless, there was no correlation between items of BDI, FSS, SF-36, CERAD-PLUS and WTV.
**Conclusion:** Although WTV is considered as a predictor of cognitive deficits in degenerative diseases, we only measured a trend towards WTV enlargement in PLWH without correlation to HIV specific characteristics. This may reflect the improved efficacy of ART.

### 051
### Paper-ID: 47288, P4

### Blocking the fourth 90: high prevalence of depression in HIV patients of the Bonn HIV-cohort

Gereon Jonas Rieke[1], Ahmad Salim[2], Carolynne Schwarze-Zander[1], Kathrin van Bremen[1], Jan-Christian Wasmuth[1], Jürgen K. Rockstroh[1], Christoph Boesecke[1]
[1]*Bonn University Hospital; German Center for Infection Research (DZIF);* [2]*Bonn University Hospital*

**Introduction:** In addition to the WHO's 90–90–90 goals a fourth 90 has been suggested focusing on good quality of life. With a lifetime prevalence of 16–20% depression is the most common mental illness. Data on depression in people living with HIV (PLWH) in Germany are sparse. Here we report the prevalence of depression in PLWH of the Bonn HIV-cohort.

**Methods:** Between July and September 2018 all patients attending the HIV outpatient clinic at Bonn university hospital for routine care were handed a sociodemographic questionnaire and a questionnaire based on the Beck Depression Inventory (BDI-II). Scores between 14 and 28 were considered mild to moderate depression while scores above were considered severe depression.
**Results:** Overall, 151 PLWH were enrolled, 24 had to be excluded due to incomplete questionnaires. Median age was 49 (IQR 40–55), 90.1% male, 9.9% female. Main HIV transmission risks were MSM (61.6%), heterosexual intercourse (21.2%) and IVDU (5.3%). Median CD4 T cell count was 652/µl (IQR: 494–841) with a median CD4-percentage of 31% (IQR 24–37%), in 96.2% HIV-RNA was undetectable (< 40 cop/ml). 24.5% had a history of treatment for a mental illness. 46/151 patients (36.2%) showed signs of depression. Among these 8 had severe depression. 25/38 (65.8%) of PLWH suffering from mild to moderate depression and 1/8 (12.5%) suffering from severe depression had not disclosed mental health issues in the questionnaire. 6/8 (75%) of patients with severe depression and 12/38 (31.6%) with mild to moderate depression conveyed that they had thought about suicide.
**Conclusion:** Our study shows that among PLWH of the Bonn HIV-cohort prevalence of depression assessed by validated scores is significantly higher than among the HIV-negative population. The high numbers of undiagnosed depression show that mental health awareness in HIV-patient care is of massive importance to sustain a good quality of life ("the fourth 90").

### 052
### Paper-ID: 47238, P5

### Which modern immunotherapies are used in people living with HIV (PLWH) with rheumatologic comorbidities? A case series

Guido Schäfer, Anja Hüfner, Stefan Schmiedel, Sabine Jordan, Olaf Degen
*Universitätsklinikum Hamburg-Eppendorf*

**Introduction:** Although the prevalence of autoimmune and rheumatologic inflammatory systemic diseases is high in the general population, there are few studies on this comorbidity in people living with HIV (PLWH). HIV infection is a common exclusion criterion in clinical trials of new immunomodulatory therapies. Therefore, published data on the success and safety of these therapies in PLWH is scarce.
**Methods:** Retrospective case series of own patients treated at our tertiary outpatient infectious diseases center regarding their HIV infection (and partly also regarding the rheumatologic / autoimmune systemic disease). A description and presentation of the course/outcome is given. Patients were identified through a systematic review of the clinical charts.
**Results:** We present (table 1) 6 patients who were managed with specific immunomodulatory therapies in our outpatient clinic during 2019/2020. Various therapeutic principles were used (including janus kinase inhibitors, TNF-alpha inhibitors, interleukin 12/23 inhibitors, interleukin 17-inhibitors). Regardless of the rheumatologic outcome, most patients showed an uncomplicated course regarding immune status and viral loads.
**Discussion:** Considering the therapeutic principles used (including janus kinase inhibitors), we present one of the most comprehensive case series of PLWH with inflammatory comorbidities published worldwide so far. In rheumatological clinical trials regarding new immunomodulatory therapies, PLWH have not been systematically studied so far, and HIV infection is a common exclusion criterion. Therefore, it can be hypothesized that PLWH are not treated with modern immunomodulatory therapies as it is recommended in recent guidelines of different rheumatologic diseases. Furthermore, safety of these drugs has not been investigated thoroughly yet. More systematic studies and analysis are needed and a multicenter cohort study is planned by our site.

**Table 1: patients characteristics**

| age/sex (year of HIV diagnosis) | rheumatic / inflammatory disease (year of diagnosis) | immunmodulatory therapy | antiretroviral therapy (during immunomodulatory course) | CD4-count (%) before immunomodulation - and 12/2020 | viral load before immunomodulation and 12/2020 // number of blips (>50cop/ml) during immunomodulation | Outcome of the rheumatic disease (12/2020) |
|---|---|---|---|---|---|---|
| 30J / M (2012) | PsA (2019) | Tofacitinib (JAKi): 08/2019 – 08/2020 | Odefsey | 801 (41%) – 1503 (48%) | < 50 (no blips) | remission (stop of antirheumatic therapie since 08/2020) |
| 32J / M (2014) | Pso (childhood) | Etanercept (TNFi) 06/2019 – 09/2019 (frequent bacterial infections / skin infections/reactions)<br>Ustekinumab (IL12/23i) – since 03/2020 | Genvoya, since 07/2019 Biktarvy | 712 (35%) – 764 (40%) | < 50 (no blips) | remission 10/20 |
| 45J / F (2014) | Pso + Acne inversa, PsA (1995) | Secukinumab (IL17i) 2015-01/2017 and since 11/2019<br>Brodalumab (IL17-25i) 06/18-11/19<br>Ixekizumab (IL17i) – 04/18-06/18<br>Adalimumab (TNFi) 01/2017-04/2018<br>MTX till 2011 (allergic reaction) | Dovato since 12/2019<br>Triumeq 05-12/2019<br>Tivicay+Descovy 10/2016 – 05/2019<br>Tivicay + Truvada 04/2014 – 10/2016 | 471 (31%) – 04/2014<br>1781 (54%) - 11/2020 | St. 07/2014 < 50 no Blips | persistent activity, increase of the dose of secukinumab 10/2020 |
| 80J / M (1997) | PsA and/or RA (> 15 years) | SSZ (since > 10 years till 11/20)<br>MTX (since 05/18)<br>Baricitinib (since 11/2020) | Atripla 02/08 – 12/16<br>Genvoya 12/16 – 10/19<br>Biktarvy 10/19 – 03/20<br>Dovato since 03/20 | 12/2008: 184 (25%)<br>09/2020: 335 (37%) | since 2009 no blips | Becouse of ongoing flares -change of therapie to MTX + Baricitinibe |
| 56J / W (2001) | MC (2014) | Adalimumab (TNFi) | Truvada + Kaletra 2007-06/18<br>Prezista/Norvir + Tivicay since 06/2018 | 1312 (58%) - 04/2018<br>1723 (58%) - 10/2020 | since 04/2018 (first presentation in our consultory) no blips | coloscopy 2018 - remission of MC |
| 32J / W (2020) | Pso (2020) | Brodalumab (IL17i) since 11/2020 | Biktarvy since 12/2020 | 719 (37%) | 133.000 cop/ml | First diagnosis of HIV simultaniously with Pso |

Abbreviations: ILi: Interleukin inhibition, JAKi: Janus kinase inhibitor, MC: Morbus Crohn, MTX: Methotrexate, PsA: psoriasis arthritis, Pso: Psoriasis, TNFi (Tumor-Nekrose-Faktor alpha Inhibitor)

### 053
### Paper-ID: 45301, P6

### Initial HIV diagnoses in Germany 2014—a retrospective analysis

Benjamin Schleenvoigt, Juliane Ankert[1], Hartmut Stocker[2], Albrecht Stoehr[3], Clara Lehmann[4], Christian Schulz[5], Hans Wesselmann[6], Marcus Bickel[7], Annette Haberl[8], Olaf Degen[9], Mathias Pletz[10], Ulrich Seybold[11], Christoph Stephan[8]
[1]*Universitätsklinikum Jena;* [2]*St. Joseph Krankenhaus Berlin;* [3]*Asklepios Klinik St. Georg/Asklepios Kliniken Hamburg GmbH;* [4]*Universitätsklinik Köln Klinik I für Innere Medizin;* [5]*Klinikum der Universität München;* [6]*Charité am Campus Virchow-Klinikum;* [7]*Infektiologikum Frankfurt;* [8]*HIVCENTER, Zentrum der Inneren Medizin/Medizinische Klinik II Universitätsklinikum Frankfurt, Goethe-Universität;* [9]*Ambulanzzentrum des UKE GmbH, Bereich Infektiologie;* [10]*Institut für Infektionsmedizin und Krankenhaushygiene, Universitätsklinikum Jena;* [11]*Sektion Klinische Infektiologie,*

*Medizinische Klinik und Poliklinik IV, Klinikum der Universität Ludwig-Maximilians-Universität München*

**Introduction:** Information on testing units in health care is scarce, particularly the group of late-presenters among the initial HIV diagnoses is still a challenge in Germany. Analysis of the impact of testing units and -reasons on the prevalence of initial HIV diagnoses and late-presentation, exemplary for the year 2014.

**Methods:** Cross sectional analysis of all individuals, treated in the Network HIV-Regional who were initially diagnosed with HIV in 2014; patient characteristics, demographic and clinical data, including information on HIV-testing were retrospectively and decentralised collected, pseudonymized and statistically evaluated.

**Results:** 971 individuals with initial HIV diagnoses from 31 specialised care centres throughout Germany (15 hospitals, 16 private practices) represent 27.5% of all National diagnoses –registrations from Robert-Koch-Institute for 2014, with similar results for CD4-cell count and HIV-transmission risk. The most common test site was at hospital (34.8%), followed by family doctor (19.6%) and medical specialist (16.1%). If the initial diagnosis was established in hospital, then the patients were in mean older than those tested on an ambulant care basis (42 vs. 37 years, $p = 0.001$), moreover the HI-viral load was higher (585 vs. 270 thousand-copies/mL, $p < 0.001$) and the CD4-cell count lower (265 vs. 414/µL, $p < 0.001$). In 208/971 individuals, at least one AIDS-defining disease was found, most frequently pneumocystis-pneumonia (45.5%), candidiasis (32.7%) and Kaposi sarcoma (10.6%). A regional comparison revealed for a younger age, a higher HIV-RNA viral load and more often clinical AIDS in eastern Germany.

**Conclusion:** This analysis from HIV-Regional for the year 2014, exemplary allows a deeper insight into initial HIV diagnoses, on the eve of important prevention tools introduction in Germany, e.g. HIV-home testing and pre-exposure prophylaxis. This cross-sectional analysis was representative for Germany and underscores the importance of specialised hospitals, in particular for eastern Germany, moreover the involvement of late presenters into HIV-health care.

### 054
### Paper-ID: 46938, P7

### Multisegmental myelitis after herpes zoster in an adult with undiagnosed HIV infection: a case report

Christine Brunner, Diyani Dewasurendra, Martin Gisinger, Maria Kitchen, Armin Muigg, Mario Sarcletti
*Medizinische Universität Innsbruck*

**History:** A 54-year old male suffered from progressive hemidysesthesia and hemiparesis of the right extremities during the past 3 months, polymorph skin lesions in the right lumbar region and weight loss of 12% of his body weight.

**Presentation and findings:** Neurological examination confirmed the hemihypoesthesia and spastic hemiparesis of the right arm and leg. Crusted skin lesions were localized in dermatomes L1-L3 as well as vesicles in dermatomes T6-T10 right. Varicella-Zoster-Virus DNA could be isolated from the vesicles as well as in the cerebrospinal fluid. Spinal MRI showed T2 hyperintense signals at the levels C4, T2, T6, and T7. An HIV infection was diagnosed with a CD4+ T-cell count of 10 cells/µL and a viral load of 5.63 log10 copies/ml. Other opportunistic infections or malignancies were ruled out.

**Treatment and clinical course:** With the diagnosis of zoster myelitis parenteral acyclovir (14 mg/kg bodyweight tid) was started immediately and 5 days later antiretroviral therapy (ART). Because of deterioration of hemiparesis, bladder dysfunction and new dysesthesia on the left side on day 9 of acyclovir therapy, a course of high dose methylprednisolone was given (14 mg/kg bodyweight intravenously for 3 days followed by 80 mg orally for 5 days). With this treatment there was no further progression of the neurological symptoms. Acyclovir was given intravenously for 4 weeks, followed by oral valacyclovir 1000 mg tid. The neurological symptoms remained stable and eventually improved slightly with intensive rehabilitation, but the patient is still wheel-chair bound and cannot control his bladder function. Under ART (bictegravir/tenofovirAF/emtricitabin) HIV viral load is undetectable, but CD4+ T-cells are still below 150cells/µl a year after ART initiation.

**Conclusion:** Myelitis is a rare complication of herpes zoster, which can lead to persistent disability. Early diagnosis and intensive treatment should prevent severe illness. As relapse can occur suppressive therapy might be reasonable.

### 055
### Paper-ID: 46712, P8

### Disseminated cryptococcal infection in HIV-1-infected man, complicated by paradoxical cryptococcal-IRIS and unmasking mycobacterium avium complex (MAC)–IRIS

Diyani Dewasurendra, Maria Kitchen, Martin Gisinger, Matthias Schmuth, Mario Sarcletti
*Medical University Innsbruck*

**Introduction:** Cryptococcosis and mycobacterium avium (MAC) infection are frequently associated with immune reconstitution inflammatory syndromes (IRIS) in AIDS patients. Elevated intracranial pressure during cryptococcosis sometimes requires frequent lumbar punctures.

**Methods:** We present a 32 years old HIV-1 infected man with disseminated cryptococcosis complicated by paradoxical crypotococcal-IRIS and unmasking MAC-IRIS.

**Results:** In 03/19 the patient presented with malaise, weight loss, diarrhoea, vomiting, dyspnoea. We diagnosed AIDS with disseminated cryptococcosis (brain, blood, lung, lymph nodes). The CSF opening pressure was 36 $cmH_2O$, therefore he received serial lumbar punctures. After 19 days of flucytosin/liposomal amphotericin-B and clinical improvement we switched to consolidation (fluconazole 400 mg/day) and after another 6 weeks to secondary prophylaxis (fluconazole 200 mg/day).

At day 35 of cryptococcosis treatment, we started antiretroviral therapy (ART: tenofovir-AF/emtricitabin/boosted darunavir). On day 30 of ART, high immune activation was detected (neopterin 259 nmol/l) and FDG-PET-CT showed inflammation pulmonar, generalised lymphadenopathy and Ileocolitis. Biopsy showed multiple cryptococci in a hilar lymph node and cryptococcal-IRIS was diagnosed. ART was continued and without additional therapy IRIS symptoms subsided during 14 days. 09/19 (25 weeks on ART) the patient developed cough and dyspnea. CTscan revealed hilar lymphadenopathy and obstruction of the right lobe bronchus by a pulmonary mass. Extensive evaluation revealed Mycobacterium avium (MAC) in the hilar lymph node (unmasking MAC-IRIS). ART was continued and after 8 weeks of Azithromycin/Ethambutol/Rifabutin and improvement, we discontinued rifabutin but continued Azithromycin/Ethambutol.

**Conclusion:** Cryptococcal- and mycobacterium avium-IRIS can cause severe disease in AIDS patients after the start of antiretroviral therapy (ART). Multiple immunological factors are causative for IRIS. To decrease the risk of cryptococcal- and/or MAC-IRIS, ART should be delayed after start of treatment against these opportunistic infections (OI).

### 056
### Paper-ID: 46596, P9

### Pyogenic spondylodiscitis in virologically suppressed HIV infected individuals: a case series

Maria Kitchen[1], Martin Gisinger[1], Diyani Dewasurendra[1], Armin Rieger[2], Mario Sarcletti[1]
[1]*Medizinische Universität Innsbruck;* [2]*Medizinische Universität Wien*

**Introduction:** The incidence of spondylodiscitis has increased in the last decades, and risk factors include older age, polymorbidity, immunosuppression, diabetes, intravenous drug use, and a history of bacterial infections. HIV infection has also been regarded a risk factor, but previous case series included patients without viral suppression or cases with mycobacterial infections in endemic countries. Here we show cases of bacterial spondylodiscitis in 5 patients under antiretroviral treatment.
**Method:** We present the demographic and clinical characteristics of spondylodiscitis seen between 2017 and 2020 in a single-centre cohort of HIV-infected adults.
**Results:** Five patients were diagnosed with spondylodiscitis of the cervical, thoracic, or lumbar spine. The mean age at diagnosis was 55 years (38–74), 3 were male, 2 female. All were virologically suppressed under HIV treatment, the mean CD4 count was 407/µL (111–710). Three patients had serious comorbidities. The causative pathogen was identified in all patients: Staphylococcus aureus in four cases, Klebsiella pneumoniae and mixed infection (Proprionibacterium acnes; Bacillus circulans) in a man who has sex with men and who presented with two episodes. Two patients were also diagnosed with endocarditis. Severe, non-radicular pain was the presenting symptom in all cases, MRT scans confirmed the diagnosis. All patients needed surgical intervention. The mean duration of hospital stay was 49 days (10–86) and the mean duration of antibiotic treatment 19 weeks (13–26) in the patients who survived. One patient died after 50 days in hospital. Details of the cases are shown in the table.
**Conclusion:** Pyogenic spondylodiscitis is a rare complication which might be seen more frequently as patients with HIV are getting older and increasingly suffer from comorbidities which put them at risk. Main symptom is a severe, non-radicular pain, and MRT scan is the gold standard for diagnosis. Awareness of the condition will help with faster diagnosis and better outcome.

| | Patient 1 | Patient 2 | Patient 3 | Patient 4 | Patient 5 | |
|---|---|---|---|---|---|---|
| Diagnosis | 4.6.2019 | 19.10.2018 | 17.10.2018 | 2.4.2020 | 25.7.2017 | 12.02.2019 |
| Age/sex | 38/m | 52/f | 56/f | 74/m | 54/m | 55/m |
| Transmission | MSM | IVDU | IVDU | MSM | MSM | |
| CD4 count | 414 | 246 | 111 | 554 | 710 | 875 |
| Years on ART | 8 | 27 | 24 | 22 | 6 | 7 |
| Comorbidity | ESRD | HCV Cirrhosis | Pneumonia | Hypertension | Acute HCV | None |
| Pain | Yes | Yes | Yes | Yes | Yes | Yes |
| Fever (>38°C) | Yes | Yes | Yes | Yes | Yes | No |
| Neurol. signs | Yes | Yes | Yes | No | No | No |
| WBC | 15.5 | 4.8 | 7.7 | 14.9 | 11.5 | 5.7 |
| CRP | 36.9 | 28.5 | 6.55 | 19.6 | 12.97 | 5.07 |
| ESR | 75 | 69 | 42 | 59 | 22 | 20 |
| Location | C3/4 C4/5 and epidural abscess | C6/7 and epidural abscess | Th9/10/11 and paravertebral | L4/5 and paravertebral | L4/5 | L3/4 |
| Pathogen | MSSA (blood/tissue) | MRSA (blood, tissue) | MSSA (blood) | MSSR (blood) | *Klebsiella sp.* (Blood, tissue) | *Propionibacterium; Bacillus circ.* (tissue) |
| Surgery | 5.6./26.6./6.7.19 | 23.11.18 | 30.10.18 | 9.4.20 | 18.8.17 | 13.2.19 |
| Duration Antibiotics | 26 weeks | 13 weeks | 17 weeks | 7 weeks until death | 13 weeks | 22 weeks |
| Endocarditis | Mitral valve; surgery 7.8.20 | Aortic valve; no surgery | Not investigated | Not investigated | No | No |
| Hospital stay | 86 days | 82 days | 30 days | 50 until death | 35 days | 10 days |
| Outcome | Healed | Healed | Healed | Death | Healed | Healed |

### HIV and COVID-19

### 057
### Paper-ID: 46921, O1

### SARS-CoV-2-seronegative HIV-1-infected subjects target CTL epitopes in the SARS-CoV-2 nucleoprotein cross-reactive to common cold coronaviruses

Katja Schmidt[1], Krystelle Nganou-Makamdop[2], Matthias Tenbusch[2], Boutaina El Kenz[3], Clara Maier[2], Dennis Lapuente[2], Klaus Überla[2], Bernd Spriewald[4], Silke Bergmann[1], Ellen G. Harrer[1], Thomas Harrer[1]
[1]*Department of Medicine 3,* [2]Institute of Clinical and Molecular Virology, [3]*Department of Medicine 3 and Institute of Clinical and Molecular Virology,* [4]Department of Medicine 5, Universitätsklinikum Erlangen, Friedrich-Alexander-University Erlangen-Nürnberg

**Introduction:** The beta-coronavirus SARS-CoV-2 induces severe disease (COVID-19) mainly in persons with risk factors, whereas the majority of patients experience a mild course of infection. As the common cold coronaviruses OC43 and HKU1 share some homologous sequences with SARS-CoV-2, beta-coronavirus cross-reactive T-cell responses could influence the susceptibility to SARS-CoV-2 infection and the course of COVID-19. As HIV-1 infection is a potential risk factor for COVID-19, we investigated beta-coronavirus cross-reactive T-cells in a cohort of HIV-1-infected patients on antiretroviral therapy.
**Methods:** We analyzed in IFN-g ELISpot assays T-cell responses against a 15 amino acid long peptide (DP15) from the SARS-CoV-2 nucleoprotein sequence with a high homology to the corresponding sequence in OC43 and HKU1.
**Results:** DP15-specific T-cells were detected in 4 out of 23 (17.4%) SARS-CoV-2-seronegative healthy donors. 44 out of 116 HIV-1-infected patients (37.9%) showed a specific recognition of the DP15 peptide or of shorter peptides within DP15 by CD8+ T-cells and/or by CD4+ T-cells. We could define several new cross-reactive HLA-I-restricted epitopes in the SARS-CoV-2 nucleoprotein. Epitope specific CD8+ T-cell lines recognized corresponding epitopes within OC43 and HKU1 to a similar degree or even at lower peptide concentrations suggesting that they were induced by infection with OC43 or HKU1.
**Conclusions:** Our results confirm that SARS-CoV-2-seronegative subjects can target SARS-CoV-2 not only by beta-coronavirus cross-reactive CD4+ T-cells but also by cross-reactive CD8+ cytotoxic T-cells (CTL). The delineation of cross-reactive T-cell epitopes contributes to an efficient epitope-specific immunomonitoring of SARS-CoV-2-specific T-cells. Further prospective studies are needed to prove a protective role of cross-reactive T-cells and their restricting HLA alleles for control of SARS-CoV-2 infection. The frequent observation of SARS-CoV-2-reactive T-cells in HIV-1-infected subjects could be a reason that treated HIV-1 infection does not seem to be a strong risk factor for the development of severe COVID-19.

### 058
**Paper-ID: 47178, O2**

### Characteristics, morbidity and mortality of SARS-CoV-2 infections in people living with HIV (PLWH) in Germany: a large cohort study

Georg Härter[1], Anja Meurer[2], Ansgar Rieke[3], Farhad Schabaz[4], Christoph Wyen[5], Markus Bickel[6], Heribert Knechten[7], Julia Roider[8], Christoph Spinner[9], Daniel Gillor[10], Stephan Grunwald[11], Ivanka Krznaric[12], Joanna Eger[2], Ines Ruck[13], Nils Postel[14], Robert Baumann[15], Gerd Klausen[16], Luca Stein[17], Markus Müller[18], Steve Rößler[19], Matthias C. Mueller[20], Kevin Ummard-Berger[21], Katrin Zutavern-Bechtold[22], Knud Schewe[23], Christian Hoffmann[23]
*[1]MVZ Medicover Ulm; [2]Zentrum für Innere Medizin und Infektiologie (ZIMI); [3]Gemeinschaftsklinikum Mittelrhein gGmbH Akademisches Lehrkrankenhaus der Univers; [4]MVZ Karlsplatz; [5]Praxis Ebertplatz; [6]Infektiologikum Frankfurt; [7]PZB Aachen; [8]Sektion Klinische Infektiologie, Ludwig-Maximilians-Universität München; [9]TU München, Universiversitätsklinik Rechts der Isar; [10]MVZ Innere Medizin Köln; [11]Zentrum für Infektiologie Berlin/Prenzelberg; [12]Praxis an der Kulturbrauerei; [13]Praxis Dr. Ruck; [14]Prinzmed; [15]Hausarztpraxis Baumann-Sels; [16]Schwerpunktpraxis für Infektionsmedizin am Oranienburger Tor; [17]Infektiologie Seestraße; [18]Gemeinschaftspraxis Schwabstraße 26; [19]Arztpraxis Rößler; [20]MVZ Clotten; [21]UBN/ PRAXIS; [22]Gemeinschaftspraxis für Hämatologie, Onkologie und Infektiologie; [23]ICH Hamburg*

**Introduction:** Case studies suggested no excessive morbidity or mortality of SARS-CoV-2 infections in PLWH with absence of severe immune deficiency.

**Methods:** Ongoing, retrospective analysis of SARSCoV-2 infections in PLWH in different German centers. Since March 2020, anonymized data on age, gender, antiretroviral therapy (ART), CD4 -cell count and HIV-RNA before SARS-CoV-2 infection, comorbidities, symptoms, and outcome have been collected.

**Results:** Until December 2020, 102 patients (84 men, 18 women, median age 47 years) were included. The median CD4 cell count was 666/µl and HIV-RNA was below 50 copies/ml in 95 patients. All patients but one were on ART at SARS-CoV-2 diagnosis. Regimens contained an INSTI in 75, a PI in 18 and a NNRTI in 18 cases. At least one comorbidity was reported for 66 patients, the most common were hypertension (n = 26) and diabetes (n = 10). The most frequent symptoms were cough (63%), fever (53%), and disturbance of smell or taste (28%). Of 21 hospitalized patients, 9 (43%) required intensive care. Compared to nonhospitalized patients, hospitalized patients were older (54 versus 45 years) and had lower current or nadir CD4 cell counts (490/µl versus 724/µl and 194/µl versus 374/µl, respectively). Where information was available, COVID-19 was asymptomatic in 10 (10%), mild in 76, severe in 2 and critical in 10, respectively. All 4 deceased patients were male, older than 55 years and had at least one comorbidity.

**Conclusions:** In this large cohort of PLWH diagnosed with SARS-CoV-2-infection, morbidity and mortality appear to be relatively high. Hospitalized patients were older and had evidence for a more pronounced immune deficiency ($p = 0.03$ for CD4 < 350/µl and CD4 Nadir < 200/µl, using Fisher's exact test). However, due to the retrospective design, possible confounding and a reporting bias cannot be ruled out. More data are necessary to evaluate risk factors for morbidity.

### 059
**Paper-ID: 47498, O3**

### SARS-CoV-2 vaccines in people living with HIV: prepare for flying on sight

Christian Hoffmann[1], Georg Härter[2], Alexander Zoufaly[3]
*[1]ICH Hamburg; [2]MVZ Medicover Ulm; [3]Kaiser-Franz-Josef-Spital Wien*

**Introduction:** Recent cohort studies have shown that people living with HIV (PLWH) may carry a risk for severe courses of COVID-19, especially in the presence of poor immune status. On the other hand, PLWH may also be at higher risk for vaccine related complications. Ad5-specific CD4 T cells have been shown to increase susceptibility to HIV infection and mRNA vaccines may bear a theoretical risk for integration into genomic DNA in the presence of retroviral enzymes such as reverse transcriptase or integrase.

**Methods:** An analysis of inclusion and exclusion criteria of all ongoing Phase III SARS-CoV-2 vaccine trials ("recruiting", "active but not recruiting", "not yet recruiting") listed at www.clinicaltrialgov.com at November 26, 2020.

**Results:** In total, 21 trials with a planned total number of 460,371 participants were listed. Of these, 11 (47.9% of all participants) trials had explicitly excluded PLWH. In one trial, the protocol was later amended or allowed in a small substudy to include PLWH. However, in the trial on BNT162b2, only 59/18.860 (0.3%) subjects receiving the mRNA vaccine were HIV infected. A further 8 (32.5% of participants) trials had excluded "any confirmed or suspected immunosuppressive or immunodeficient state". Only 2/21 trials (both with an adenovirus serotype 26 vector-based vaccine) did not list these exclusion criteria. Both trials will recruit patients in at least one high HIV prevalence country.

**Conclusions:** It seems likely that, except for one adenovirus vector-based vaccine, data for SARS-CoV2 vaccine in PLWH will remain very limited in 2021. Most ongoing trials still exclude those subjects which may need the vaccine the most, namely the immunosuppressed. Without careful pharmacovigilance and post-marketing studies, SARS-CoV-2 vaccination of PLWH, in particular those with severe immune deficiency, will remain a blind flight.

### 060
**Paper-ID: 46630, O4**

### PrEP use and care in times of the SARS-CoV-2-pandemic

Daniel Schmidt[1], Marie Merbach[1], Martin Friebe[1], Knud Schewe[2], Nikola Hanhoff[3]
*[1]Robert Koch Institut (RKI); [2]ICH Study Center Hamburg; [3]Deutsche Arbeitsgemeinschaft niedergelassener Ärzte in der Versorgung HIV-Infizierter e.V. (dagnä)*

**Introduction:** In times of the SARS-CoV-2-pandemic, social life in Germany has changed significantly. Subsequent measures such as closing of bars and clubs as well as general advice for physical distancing have impacted sexual contacts. In healthcare, many HIV-specialists focused their clinical practice on Covid19. We investigated the influence of the pandemic on PrEP use and care as part of the national evaluation of HIV pre-exposure prophylaxis (PrEP) as a service of the statutory health insurance in Germany ("EvE-PrEP").

**Methods:** In April, July and October 2020 questionnaires were sent to 51, 54 and 57 HIV-specialized practices participating in EvE-PrEP. Data surveyed are shown in table 1.

**Results:** On average, 36 centres (67%) responded to the questionnaires. All provided continued PrEP-services in times of Covid-19. In April a decrease in PrEP demand, mostly secondary to the SARS-CoV-2pandemic, was reported by 28 centers (76%) which in July was reversed by an increase seen in 20 centers (56%). A waiting time of $\leq$ 2 weeks for an appointment for PrEP initiation remained stable throughout the pandemic: April, July and October in 68%, 87% and 76% of centers respectively. The total number of PrEP initiations decreased by 55% from 4,218 (September–December 2019) to 1,922 (January–March 2020) and was at 1,193 (April–June 2020) and at 1,247 (July–September 2020). The total number of PrEP-users was 6,590 (September–December 2019), 7,656 (January–March 2020), 7,561 (April–June 2020), and 7,656 (July–September 2020) in the participating centers.
**Conclusion:** Despite the strain of the pandemic on the health care system, the provision of PrEP care could be maintained by HIV-specialized centers in Germany. Reductions in the number of persons initiating PrEP during 2020 may be due to behavioral changes induced by the pandemic, a saturation effect after coverage by statutory health insurance in September 2019 or other unknown barriers to access PrEP.

*Table 1: Questions and topics addressed in the surveys (April - July – October)*

| Survey questions | April 2020 | July 2020 | October 2020 |
|---|---|---|---|
| Interruption of PrEP-service | x | | |
| Case numbers (total number of PrEP-users + number of PrEP-initiations ) | x | x | x |
| Effects on/development of PrEP demand | x | x | |
| Reasons for decreasing demand | x | x | |
| Cancelled control visits | x | | |
| Feasibility of PrEP care (necessity for reorganization of internal structures) | x | | |
| Waiting time for consultation/control | x | x | x |
| Decrease/Increase/no change in waiting time | | x | |
| Reasons for non-prescription | x | | |
| New HIV-infections (before and under PrEP) | | | x |
| Percentage of PrEP-Users insured by statutory health insurance | | | x |

### 061
### Paper-ID: 47487, O5

### High dose lopinavir/ritonavir does not lead to sufficient plasma levels to inhibit SARS-CoV-2 in hospitalized COVID-19 patients

Mario Karolyi[1], Sara Omid[1], Erich Pawelka[1], Dr. Bernd Jilma[2], Thomas Stimpfl[2], Christian Schörgenhofer[2], Tamara Seitz[1], Marianna Traugott[1], Christoph Wenisch[1], Alexander Zoufaly[1]
*[1]Kaiser-Franz-Josef-Spital Wien; [2]AKH Wien*

**Background:** Despite in-vitro activity of Lopinavir/Ritonavir (LPV/RTV) against SARS-CoV-2 large trials failed to show any net clinical benefit. Since SARS-CoV 2 has an EC50 of 16.4 µg/mL this could be due to inadequate dosing.
**Methods:** COVID-19 positive patients admitted to the hospital who received high dose LPV/RTV were included in this ongoing platform trial. High dose (HD) LPV/RTV 200/50 mg was administered 4 tablets bid as loading dose, then 3 tablets bid for up to 10 days. Trough plasma concentration was measured after the loading dose and on day 5–7 in steady state (SS). Post loading dose (PLD) and SS plasma trough levels were compared with SS trough levels from COVID-19 patients who received normal dose (ND) LPV/RTV (2 tablets bid) at the beginning of the pandemic.
**Results:** Fifty-one patients (31.4% female) with a median age of 58 years (IQR 49–71) received HD LPV/RTV. HD-PLD and HD-SS trough level was measured in 43 and 33 patients respectively. ND-SS was available from 8 patients. Median HD-PLD concentration was 24.9 µg/mL (IQR 15.8–30.3) and significantly higher than HD-SS (12.9 µg/mL, IQR 7.2–19.5, $p < 0.001$) and ND-SS (13.6 µg/mL, IQR 10.1–22.2, p = 0.013). HD-SS and NDSS plasma levels were not statistically different (p = 0.507). C-reactive-protein showed a positive correlation with HD-SS (Spearman correlation coefficient rS = 0.42, p = 0.014) and ND-SS (rS = 0.81, p = 0.015) but not with HD-PLD (rS = 0.123, p = 0.43).
**Conclusion:** HD-PLD plasma trough concentration was significantly higher than HD-SS and ND-SS concentration, but no difference was detected between HD-SS and ND-SS trough levels. Due to the high EC50 of SARS-CoV-2 and the fact that LPV/RTV is highly protein bound, it seems very unlikely that LPV/RTV exhibits any antiviral effect in vivo in COVID-19 patients.

### 062
### Paper-ID: 47310, O6

### C5aR inhibition of non-immune cells suppresses inflammation and maintains epithelial integrity in SARS-CoV-2-infected airway epithelia

Wilfried Posch, Jonathan Vosper, Viktoria Zaderer, Cornelia Lass-Flörl, Doris Wilflingseder
*Medizinische Universität Innsbruck*

**Introduction:** Excessive inflammation triggered by a hitherto undescribed mechanism is a hallmark of severe SARS-CoV-2 infections and is associated with enhanced pathogenicity and mortality. Complement hyper activation promotes lung injury and was observed in patients suffering from MERS-CoV, SARS-CoV-1 and SARS-CoV-2 infections.
**Methods:** Human primary normal bronchial (NHBE) and small airway epithelial (SAE) cells were infected with SARS-CoV-2 and analyzed for infection, inflammation, tissue integrity and C3 expression by high content screening at various days post infection.
**Results:** Here we show that NHBE and SAE cells respond to SARS-CoV-2 infection by an inflated local C3 mobilization. SARS-CoV-2 infection resulted in exaggerated intracellular complement activation and destruction of the epithelial integrity in monolayer cultures of primary human airway cells and highly differentiated, pseudostratified, mucus-producing, ciliated respiratory tissue models. SARS-CoV-2-infected 3D cultures secreted significantly higher levels of C3a and the pro-inflammatory cytokines IL-6 and MCP-1.
**Conclusions:** Crucially, we show here for the first time, that targeting the anaphylotoxin receptors C3aR and C5aR in non-immune respiratory cells can prevent intrinsic lung inflammation and tissue damage. This opens up the exciting possibility in the treatment of COVID-19.

### 063
### Paper-ID: 44655, O7

### Regional differences in age distribution and COVID-19 mortality in Germany

Christian Hoffmann[1], Eva Wolf[2]
*[1]ICH Study Center; [2]MUC Research GmbH*

**Introduction:** The striking worldwide differences in country-specific case fatality rates (CFR) have caused many speculations. A variety of heterogeneous reasons have been postulated including viral, medical, genetic, socioeconomic and environmental factors.
**Methods:** We have analyzed the age- and gender-specific data provided by the Robert Koch Institute at the federal state and district level in Germany at the end of the first wave (end of August 2020). A linear regression model was used to depict CFR and the proportion of elderly SARS-CoV-2 cases (80 years or older).

**Results:** There were huge differences between the federal states as well as between the districts. The CFR differed almost threefold between federal states and ranged from 2.02% (MecklenburgVorpommern) to 5.70% (Saarland). The range at the district level ranged from 0.71% (Gütersloh) to 12.16% (Tirschenreuth). The proportion of elderly cases also differed markedly, ranging from 5.79% to 15.05% at the state level and 2.07% to 21.19% at the district level, respectively. The linear regression model showed a strong association between the proportion of elderly cases and CFR (R2 0.767 for states and 0.739 for districts, Fig. 1). Each percentage point increase in the elderly proportion was associated with an increase in the CFR of 0.41 (95% CI 0.28–0.54) at the state level and 0.43 (95% CI 0.36–0.50) at the district level.
**Conclusions:** The CFR and the percentage of elderly people in SARS-CoV-2 infection cases varied considerably on both state and district level in Germany during the first wave. There was a linear relationship, and 76.7% and 73.9% of the variance of the CFR was explained by the share of elderly people. Many of the discussed effects on country-specific CFR appear to be overlaid by different age structures of SARS-CoV-2 infections. An update (including data from the second wave) will be presented.

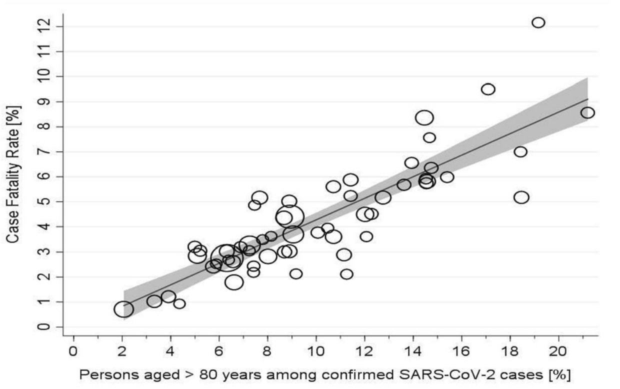

Figure 1. CFR and the proportion of persons over 80 years of age among confirmed SARS-CoV-2 cases in all German districts (with >1.000 SARS-CoV-2 cases on August 21, 2020). The linear fit prediction plot with the 95% confidence interval was estimated by weighted linear regression (weight = number of COVID-19-associated deaths). Circle sizes reflect the district-specific numbers of COVID-19-associated deaths.

**064**
**Paper-ID: 47706, P1**

### HIV therapy adherence during the Covid-19 pandemic

Karoline Gruenberger[1], Piotr Chichon[2], Alexander Zoufaly[1]
*[1]Department for Medicine 4, Klinik Favoriten, Vienna; [2]Department for Respiratory Diseases, Klinik Penzing, Vienna*

**Introduction:** The global pandemic of Covid-19 has brought health care systems all over the world to its limits. Even though the limitations of care for patients with diseases other than COVID-19 cannot be fully estimated as yet. Especially for patients diagnosed with HIV, who under normal circumstances are regularly seen at specialized health care centers, it is of utmost importance to remain on antiretroviral therapy (ART) in order to keep the viral load suppressed and to prevent transmission or development of resistance.
**Methods:** HIV positive patients who were under regular follow-up at the Infectious Diseases departments of Klinik Favoriten or the Klinik Penzing in Vienna for at least 12 months were screened for ART adherence beginning from March 2020 when the first lockdown was initiated. The total number of consultations was calculated per year and month. Patients were split up in 4 groups depending on their frequency of consultation and therapy adherence as indicated by detectable or undetectable viral loads since March 2020. In the first group were those patients who had successfully kept their viral load suppressed at all their consultation(s). The second group comprised all patients who had detectable viremia on at least one occasion and group 3 comprised patients who have had detectable viremia after March 2020 and on more than one occasion even before 2020 indicating ongoing compliance problems. In group four, patients who were always fully suppressed before 2020 but did not show up for the visit after March 2020 were included.
**Results:** Since 2017 1083 HIV positive patients have been in regular care at both treatment centers. From January-October 2019 there were a total of 2755 on-site consultations vs. 1936 on-site consultations in the same period of time in 2020. The mean frequency of visits in 2019 has been 266 patients per month vs. 188 in 2020. During the first Lockdown in March/April 2020 the on-site consultation frequency was immensely reduced to 97 bzw. 39 patients per month (Fig. 1) 85% of the patients in regular care have had a suppressed viral load (below 200cop/ml) throughout the year 2020. In 2% of the formerly successfully treated patients (n = 18) a virological failure has occurred because of new non-adherence or a therapy stop. In further 2% (n = 19) persistent viremia indicating non-adherence was noted which had already been reported from these patients before the covid-19 pandemic. 11% of all patients who before 2020 had been seen on a regular basis (n = 121), did not show up for consultation after the first lockdown in March 2020 until the end of the study mid- November (Fig. 2).
**Conclusion:** The majority of HIV positive patients with regular medical consultation could maintain their therapy successfully throughout the months of the pandemic. In 2% treatment was discontinued or irregularly taken in patients who had formerly had good adherence possibly due to limited possibilities for consultation. In most of these cases the problem could have been solved by continuing the antiviral therapy immediately after the detectable viremia has been detected at the following medical consultation. However, the real dimensions of the collateral damage during the Covid-19 pandemic concerning the HIV treatment adherence cannot yet be measured since 121 patients still did not make an appointment at the endpoint of the study mid-November 2020.

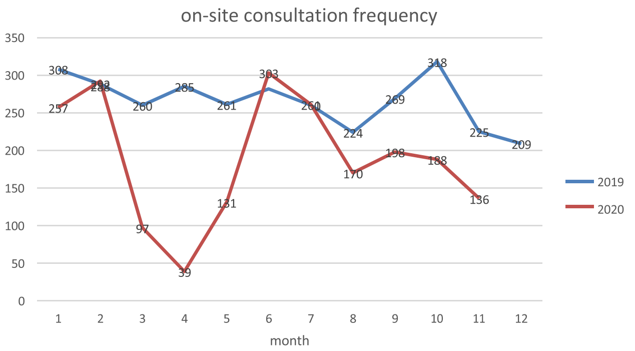

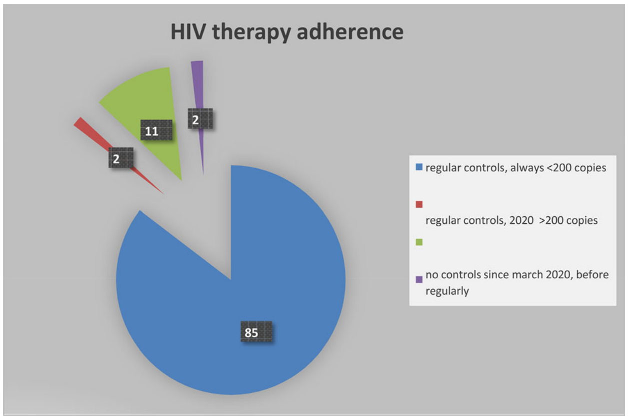

### 065
### Paper-ID: 46923, P2

### IgG seroprevalence of SARS-CoV-2 among individuals with HIV and sustained virologic suppression in Southwest Germany—a seroprevalence study

Lukas Keuser[1], Dominic Kaddu-Mulindwa[2], Jürgen Rissland[3], Stefan Lohse[3], Victoria Werdecker[2], Benedikt Lohr[4], Jens Lutz[1], Ansgar Rieke[1]
*[1]Gemeinschaftsklinikum Mittelrhein gGmbH, Kemperhof Koblenz; [2]Department of Internal Medicine I, Saarland University Medical Center, Saarland University; [3]Department of Virology, Saarland University Medical Center, Saarland University; [4]MVZ für Laboratoriumsmedizin und Mikrobiologie Koblenz-Mittelrhein*

**Introduction:** Seroprevalence studies of SARS-CoV-2 which causes COVID-19 have shown that there is a high number of undiagnosed cases because of the high proportion of asymptomatic or mild infections (approximately 80%). Serological detection of specific antibodies against SARS-CoV-2 might be useful to estimate the true number of infections. Seroprevalence of SARS-CoV-2 in individuals with HIV is lacking. Therefore, we conducted a prospective study to estimate the seroprevalence of SARS-CoV-2 among individuals with HIV in the southwest of Germany.
**Methods:** Serologic testing for immunoglobulin G antibody based on two assays was conducted in 595 (488 male and 107 female) individuals with HIV aged 18–82 years who visited outpatient HIV center of two hospitals from April to July 2020. Three patients had previously been diagnosed with SARS-COV2 infection. Patients were examined by an experienced physician and handed out questionnaires about possible COVID-19-related symptoms and risk factors, e.g. personal contact to COVID-19 patients, recent travels to high risk areas or healthcare workers. Additionally, we tested 50 non-HIVinfected patients receiving post- or pre-exposure HIV prophylaxis.
**Results:** The seroprevalence (in individuals with HIV) was 1.85% (11/595) among them, only 4 had COVID-19related symptoms. One patient with confirmed COVID-19 did not show serological antibody response in repeatedly carried out tests. 3 patients receiving a pre- or post-exposure prophylaxis (3/50) were seropositive (6%), 2 of them were symptomatic. None of the seropositive patients was hospitalized due to COVID-19.
**Conclusion:** Despite the limitation of a small and unrepresentative sample, to our knowledge this is the first study on seroprevalence of SARS-CoV-2 in individuals with HIV. Our study suggests that the seroprevalence of SARS-CoV-2 in HIV individuals is comparable to previously published seroprevalence in the general population in Germany (SeBluCo-Study, German Robert Koch Institut: first intermediate status, 2020-06-30: 1.3%, second intermediate status 2020-11-05: 1.35% seroprevalence).

### 066
### Paper-ID: 46911, P3

### Prevalence of SARS-CoV-2 antibodies in HIV PrEP users during the COVID-19 pandemic: THE PREP-CO STUDY

Simon Weidlich[1], Jochen Schneider[1], Marcel Lee[1], Christiane Schwerdtfeger[1], Birgit Waschulzik[2], Dieter Hoffmann[3], Hrvoje Mijočević[3], Ulrike Protzer[3], Christoph D. Spinner[1]
*[1]Klinikum rechts der Isar-School of Medicine, Technical University of Munich; [2]Institut für Medizinische Informatik, Statistik und Epidemiologie der Technischen Universität München; [3]Institute for Virology, Technical University of Munich*

**Introduction:** The COVID-19 pandemic required control of transmission. Men having sex with men (MSM) are a people group with distinct dating behavior, possibly marking higher infection risk.
**Methods:** After obtaining written informed consent, SARS-CoV-2 serology (Shenzhen YHLO Biotech Co., Shenzhen, China; IgG, IgM) was analyzed in MSM using HIV pre-exposition prophylaxis (PrEP) at a single tertiary university hospital in Munich, Germany, during three-monthly routine PrEP visits. Sexually transmitted diseases (STDs), personal risk behavior and number of sexual contacts were recorded. For this analysis SARS-CoV-2 antibody prevalence was analyzed May–October 2020 in 129 HIV-PrEP users and personal risk behavior was descriptively analyzed.
**Results:** Prevalence-per-month for SARS-CoV-2 IgG was 0% (0/19 subjects) in May, 1% (1/28) in June, 2% (2/32) in July, 0% (0/33) in August, 1% (1/11) in September and 1% (1/6) in October, resulting in an seroprevalence of 3.9% (5/129). None of the PrEP-users lost IgG-titer at month 3 follow-up visit. IgM was detected in three subjects (isolated IgM in one and co-existence with IgG in two subjects). Three subjects self-reported history of COVID-19, of which in two we detected IgG. In two subjects IgG was found without a known COVID-19 infection. At visit on month 3, 44% of subjects estimated their risk for COVID-19 infection as rather low and 40% as intermediate. On records, 2 syphilis infections in July, one N. gonorrhea (NG) and C. trachomatis infections were recorded May to July, whereas 2 with NG and 4 with CT were recorded in August. In September and October only 1 CT was recorded. Self-reported sexual risk behavior is displayed in Table 1.
**Conclusions:** Our monocentric HIV-PrEP cohort showed a seroprevalence of 3.9% within the first 7 months of the Covid-19 pandemic. Self-reported sex behavior remained stable in the reported period, while the number of absolute STIs tended to increase.

**Table 1:** Self-reported sexual risk behavior.

| Year 2020 | May | June | July | August | September | October |
|---|---|---|---|---|---|---|
| *Time since last sex reported by subjects* | | | | | | |
| < 24 hours | 3 (12,5%) | 3 (11,5%) | 7 (26,9%) | 10 (2,5%) | 1 (2,4%) | 4 (9,8%) |
| < 7 days | 9 (37,5%) | 15 (57,5%) | 13 (50%) | 25 (50%) | 25 (61%) | 25 (61%) |
| < 4 weeks | 4 (16,6%) | 7 (26,9%) | 6 (23%) | 8 (16%) | 7 (17%) | 10 (24%) |
| < 3 months | 3 (12,5%) | 1 (3,8%) | 0 (0%) | 7 (14%) | 5 (12,5%) | 1 (2,4%) |
| | | | | | | |
| *Number of sex partners within the last 3 months reported by subjects* | | | | | | |
| 1 | 3 (17,6%) | 8 (27,6%) | 9 (20,5%) | 9 (20,5%) | 6 (16,2%) | 5 (11,6%) |
| 2 | 6 (35,3%) | 4 (12,5%) | 7 (15,9%) | 7 (15,9%( | 6 (16,2%) | 9 (20,9%) |
| 3-5 | 4 (23,5%) | 12 (37,5%) | 9 (20,5%) | 11 (29,7%) | 11 (29,7%) | 14 (32,5%) |
| 6-10 | 4 (23,5%) | 2 (6,3%) | 7 (15,9%) | 8 (21,6%) | 8 (21,6%) | 10 (23,2%) |
| 11-20 | 0 (0%) | 4 (12,5%) | 7 (15,9%) | 3 (8,1%) | 3 (8,1%) | 3 (7,0%) |
| >20 | 0 (0%) | 2 (6,3%) | 5 (11,4%) | 3 (8,1%) | 3 (8,1%) | 2 (4,6%) |

### 067
### Paper-ID: 46925, P4

### Sex, drugs and rock 'n' roll? HIV-PrEP in the era of COVID-19

Luca Cristodero, Kathrin van Bremen, Carolynne Schwarze-Zander, Jan-Christian Wasmuth, Jürgen K. Rockstroh, Christoph Boesecke
*Bonn University Hospital*

**Introduction:** A fixed-dose combination of FTC/TDF for HIV pre-exposure prophylaxis (PrEP) has been covered by German health insurances since September 2019. We aimed to assess the individual sexual risk behavior, the incidence of sexual transmitted diseases (STDs), the adherence and side effects among PrEP users at the HIV outpatient clinic of the University Hospital Bonn.
**Methods:** Single-center, cross-sectional study including data obtained by a self-designed questionnaire and PrEP users' medical history at the HIV outpatient clinic Bonn between September 2019 and August 2020. PrEP users underwent STD/HIV testing in quarterly intervals.
**Results:** Overall, 118 PrEP users were included. 99.2% (n = 117) were MSM, 0.8% (n = 1) were transfemale. Median age was 36 years (IQR 35–40). Median observation period was 9 (IQR 7–11) months. 78% (n = 93) filled out the questionnaire: 94% (n = 88) stated to have had more than one sexual partner in the recent year; the median number of sexual partners was 8 (IQR 6–10) within 12 months before starting PrEP; 61% (n = 58) had unprotected anal sex occasionally. 19% (n = 22) had at least once used HIV post-exposure prophylaxis (PEP). Generally, 35% (n = 41) were diagnosed with a STD in their lifetime before starting PrEP. Under PrEP, 10% (n = 12) were diagnosed with a STD (chlamydia, gonorrhea, syphilis and/or hepatitis C); no one was diagnosed with HIV. 25% (n = 30) stopped PrEP for an extended time period of at least one week, 50% (n = 15) of them specifically due to the COVID-19 pandemic. 6% (n = 7) were using PrEP-on-demand.
**Conclusion:** Our data do not show a PrEP-related increase in incidence of STDs during the observation period. Noteworthy, a quarter of all PrEP users stopped their medication temporarily; only in half of these cases due to the COVID-19 pandemic potentially indicating continuous online dating in private settings. Therefore, PrEP delivering services should not be curtailed in the ongoing pandemic.

### 068
### Paper-ID: 46464, P5

### The COVID-19 pandemic and AIDS Service Organizations (ASO): threat or opportunity for the future? Survey among the ASO organizations in Lower Saxony

Matthias Stoll[1], Christian Gailus[2], Ingrid Mumm[3], Carsten Andreas Werner[4], Claudia Holzki[2], Christian Wichers[5], Jürgen Hoffmann[6], Andreas Paruszewski[2], Christin Engelbrecht[2]
[1]*Medizinische Hochschule Hannover (MHH), Aidshilfe Niedersachsen, Landesverband e.V. (AHN);* [2]*Aidshilfe Niedersachsen, Landesverband e.V. (AHN);* [3]*Your Health-Your Right, Positive Network for Children and Families;* [4]*caanwe-marken.zeichen;* [5]*Hannöversche Aidshilfe (HAH), Aidshilfe Niedersachsen, Landesverband e.V. (AHN);* [6]*Aidshilfe Braunschweig (AH-BS), Aidshilfe Niedersachsen, Landesverband e.V. (AHN)*

**Introduction:** COVID-19 led to a lockdown in spring and autumn 2020. One consequence for ASOs (Aidshilfen) was the additional effort in counselling, in particular within support groups. Particular projects are restricted by the lockdown and financial support for them is threatened. In addition, the collapse of the economy and the tax revenue threatens to restrict private donations and public funding in the future. Flat-rate cuts in the government budget for ASOs were announced in October 2020 for 2021. On the other hand, ASOs were faced with an increased need for advice by their clients due to COVID19 and were increasingly consulted as experts for prevention adapted to the pandemic in the ASO target groups by NGOs and decision-makers.
**Methods:** In October 2020, a survey was carried out between full-time and volunteer employees of ASOs in Lower Saxony using a structured questionnaire with 15 questions about the structure of their own ASO and their previous experience with the possible effects of COVID-19 on their specific work.
**Results:** 22 participants answered: 27% of them on the board of their ASO, 82% employed and 18% voluntary. 68% work in an ASO with $\leq$ 5 employees, 50% with $\leq$ 2 employees. ASOs with $>$ 5 full-time employees (32%) had been involved in advising NGOs and stakeholders more frequently, while smaller ASOs had more frequently conducted personal counselling and carried out tests among their clients. Table 1 shows more details.
**Conclusions:** COVID-19 represents a noticeable obstacle to the counselling and work of the ASOs, which in turn endangers their funding. At the same time, counselling and expert advice on COVID-19 lead to additional work. The majority of those questioned saw short-term risks here that need to be cushioned, as a long-term need for the work of ASOs is presumed.

Table 1:
***Effects of COVID-19 on the work of AIDS organizations and their financial support.***
Estimates of board members and responsible employees of ASOs in a survey in Oct. 2020; Data in percent of respondents.

| Issue | Percent |
|---|---|
| Restriction of existing offers | 82% |
| Endangerment of existing projects | 77% |
| Financing of existing projects at risk | 27% |
| Financial recovery of current project funds | 46% |
| Additional tasks and additional work due to COVID-19 | 32% |
| Increased inquiries / advice on COVID-19 | 55% |
| Confidence in the persistence of governmental financial support under COVID-19 | 46% |
| Insufficient support within the ASO network | 5% |
| COVID as an opportunity for the future work and fundings of the ASO | 52% |

### 069
### Paper-ID: 46711, P6

### Low incidence of SARS-CoV-2 seroconversion in healthcare professionals in the Rhineland area (The CoSHeP study)

Kathrin van Bremen[1], Malte Monin[1], Anna Maria Eis-Hübinger[2], Benjamin Marx[2], Souhaib Aldabbagh[2], Hendrik Streeck[2], Jan-Christian Wasmuth[1], Stefan Schlabe[1], Gereon Jonas Rieke[1], Carolynne Schwarze-Zander[1], Jürgen K. Rockstroh[1], Christoph Boesecke[1]
[1]*Universitätsklinikum Bonn;* [2]*Universität Bonn, Institut für Virologie*

**Introduction:** With the ongoing SARS-CoV-2-pandemic, healthcare professionals (HP) caring for hospitalized COVID-19-patients might be at higher risk for acquiring and spreading SARS-CoV-2 infections. Data on SARS-CoV-2 seroprevalence in HP in Germany are sparse. The CoSHeP study provides novel data on SARS-CoV-2 seroconversion rates in HP at the University Hospital Bonn, a maximum healthcare provider in a region of 900,000 inhabitants.
**Methods:** Single-center, longitudinal observational study investigating the rate of SARS-CoV-2. IgG/seroconversion in HP at 3 time-points. SARS-CoV-2 IgG was measured with Roche Elecsys Anti-SARS-CoV-2 assay. Follow-up testings are planned in November 2020 and February 2021. The full dataset will be presented at the conference in March 2021.
**Results:** 152 HP were included. 62% (n = 94) were female, 38% male (n = 58). Median age was 35 (IQR: 19–68). 54% (n = 82)

worked on intensive care unit, 30% (n = 46) in emergency room, 9% (n = 14) on infectious diseases ward, 7% (n = 10) in infectious disease outpatient clinic. Participants treated a median of 44 (1–900) suspected and 9 (0–75) confirmed cases of COVID-19. 78% (n = 113) doubted having underwent infection with SARS-CoV-2. SARS-CoV-2-IgG was detected in 5 participants (3%; 2 physicians, 3 nurses) in the first round in June 2020, 80% (n = 4) had known their SARS-CoV2-infection before. 60% (n = 3) of infected participants reported contact with suspected or diagnosed SARS-CoV-2-infected in private environment. One person (20%) had been in a high-risk region.
**Discussion:** The low overall incidence of 3% SARS-CoV-2 IgG positivity among HP reveals that professional hygiene standards are effective in preventing SARS-CoV-2 infections in HP taking care of hospitalized COVID-19-patients in areas with low rates of SARS-CoV-2 infection. Noteworthy, based upon identified chains of infection most of the infections were acquired in private environment. These findings suggest that HP were not at significantly higher risk for getting infected with SARS-CoV2 during first wave of the SARS-CoV-2 pandemic.

**070**
**Paper-ID: 47318, P7**

**Role and benefits of infectious diseases (ID) specialists in the COVID-19 pandemics**

Lene Tscharntke[1], Norma Jung[1], Siegbert Rieg[2], Christoph Spinner[3], Frank Hanses[4], Stefan Borgmann[5], Kai Wille[6], Maria Rüthrich[7], Martin Hower[8], Maria Vehreschild[9], Michael von Bergwelt-Baildon[10], Uta Merle[11], Silvio Nadalin[12], Julia Fürst[13], Hartwig Klinker[14], Kerstin Hellwig[15], Ribel Elena[16], Claudia Raichle[17], Lukas Eberwein[18], Anette Friedrichs[19], Dominic Rauschning[20], Katja de With[21], Beate Schultheis[22], Milena Milovanovic[23], Katja Rothfuss[24], Christian Riedel[25], Jörg Schubert[26], Maximilian Worm[27], Thomas Glück[28], Gernot Beutel[29], Marc Bota[30], Michael Schmid[31], Michael Doll[32], Martin Sprinzl[33], Stephan Steiner[34], Stefani Röseler[35], Helga Peetz[36], Caroline Kann[37], Harald Schäfer[38], Ingo Greiffendorf[39], Maximilian Schons[1], Carolin E.M. Jakob[1], Lisa Pilgram[9], Melanie Stecher[1], Jörg Janne Vehreschild[9]
*[1]University Hospital of Cologne; [2]University of Freiburg; [3]Technical University of Munich, School of Medicine, University hospital rechts der Isar; [4]University Hospital Regensburg; [5]Ingolstadt Hospital; [6]University of Bochum; [7]University Hospital Jena; [8]Hospital Dortmund gGmbH; [9]University Hospital Frankfurt, Goethe University Frankfurt; [10]Ludwig Maximilians University; [11]Heidelberg University Hospital; [12]University Hospital Tübingen; [13]University Hospital Erlangen; [14]University of Würzburg Medical Center; [15]Katholisches Klinikum Bochum, Ruhr University Bochum; [16]Hunsrück Hospital Kreuznacher Diakonie; [17]Tropenklinik Paul-Lechler Hospital; [18]Klinikum Leverkusen gGmbH; [19]University Hospital Schleswig–Holstein; [20]Bundeswehr Central Hospital Koblenz; [21]University Hospital Carl Gustav Carus, Technical University of Dresden; [22]University Hospital Marien Hospital Herne; [23]Malteser Krankenhaus St. Franziskus Hospital; [24]Robert Bosch Hospital; [25]Helios Hospital Pirna; [26]Elbland Hospital Riesa; [27]Oberlausitz-Kliniken gGmbH; [28]Hospital Südostbayern AG; [29]Hannover Medical School; [30]Bethesda Hospital Bergedorf; [31]Hegau-Bodensee-Hospital; [32]St. Josefs-Hospital Wiesbaden; [33]University hospital Mainz; [34]St. Vincenz Hospital; [35]Hospital Augustinerinnen; [36]Hospital Preetz; [37]Malteser Hospital St. Hildegardis; [38]SHG Hospital Völklingen; [39]Maria-Hilf Hospital Mönchengladbach*

**Introduction:** The on-going pandemic poses a major public health threat, raising questions regarding current structures of healthcare systems. Since it has been shown that patients with severe infections benefit from being treated by ID specialists, their contribution to decision making processes should be evaluated.
**Methods:** Analyses were based on the multi-center Lean Open Survey on SARS-CoV-2 infected patients (LEOSS). In a first step, the involvement of ID specialists in the care of SARS-CoV-2 infected patients was determined by a survey, which was conducted at 39 LEOSS study sites. In a second step, the data on hospital level was combined with patient data from LEOSS.
**Results:** A total of 39 German hospitals with a size range of 90 to 2,100 beds participated in the survey. In 62.2% (27/39) ID specialists were involved in the care of SARS-CoV-2 infected patients. 15 hospitals were externally ID certificated. Among those, ID specialists were significantly more likely to be involved in organizing COVID-19 inpatient areas [80.0% (12/15) vs 37.5% (9/24), p = 0.019], in treatment recommendations [86.7% (13/15) vs 50.0% (12/24), p = 0.038], and study planning (73.3% (11/15) vs 16.7% (4/24), p = 0.001]. 2,028 patients were included at the 39 LEOSS sites (Table 1), 58.5% were male (1,168/2,028); the most common age category was 76–85 [21.6%, (439/2,028)]. 64.8% (1,315/2,028) SARS-CoV-2 infected patients were documented in certified centers, of whom 25.6% (337/1,315) had a severe course of the disease compared to 35.2% (713/2,028) patients enrolled in non-certified centers, of whom 21.9% (156/713) had a severe course of the disease.
**Conclusion:** Our first results indicate a higher involvement of ID specialists in the care of SARS-CoV-2 infected patients in certified centers. In further analyses, we will use the combination of data on hospital and patient level to investigate the influence of this involvement on process quality and outcomes of patients.

**Table 1.** Characteristics of the included SARS-CoV-2 infected patients in ID certified centers and not ID certified centers.

| | Total | ID certified centers | Not ID certified centers |
|---|---|---|---|
| **Included cases** | 2,028 | 1,315 (64.8%) | 713 (35,2%) |
| **Age** | | | |
| *76 - 85 years* | 439 | 229/1,315 (17.4%) | 210/713 (29.5%) |
| *66 - 75 years* | 357 | 217/1,315 (16.5%) | 140/713 (19.6%) |
| *56 - 65 years* | 419 | 289/1,315 (22.0%) | 130/713 (18.2%) |
| *46 - 55 years* | 313 | 232/1,315 (17.6%) | 81/713 (11.4%) |
| **Sex** | | | |
| Female | 842 | 533/1,315 (40.5%) | 309/713 (43.3%) |
| Male | 1,168 | 782/1,315 (59.5%) | 404/713 (56.7%) |
| **Severe course of disease*** | 493 | 337/1,315 (25.6%) | 156/713 (21.9%) |

*need for catecholamines, life-threatening cardiac arrhythmia, need for unplanned mechanical ventilation (invasive or non-invasive), prolongation (>24h) of planned mechanical ventilation, Liver failure, qSOFA >=2, acute renal failure in need of dialysis

### Sexually Transmitted Diseases

**071**
**Paper-ID: 46655, O1**

**Changes of self-reported sexually transmitted bacterial infections from 2010 and 2017 in two large European samples of men having sex with men**

Ulrich Marcus[1], Massimo Mirandola[2], Susanne B. Schink[3], Lorenzo Gios[4], Axel J. Schmidt[5]
*[1]Robert Koch Institut; [2]University of Verona, Italy; [3]Robert Koch Institute; [4]University of Verona; [5]London School of Hygiene and Tropical Medicine (LSHTM)*

Many European countries reported increased numbers of syphilis, gonorrhea and chlamydia diagnoses among men who have sex with men (MSM) in recent years. Behavior changes and increased testing are thought to drive these increases.
In 2010 and 2017, two large online surveys for MSM in Europe (EMIS-2010, EMIS-2017) collected self-reported data on STI diagnoses in the previous 12 months, diagnostic procedures, STI symptoms when testing, number of sexual partners, and sexual behaviors such as condom use during the last intercourse with a non-steady partner in 46 European countries. Multivariate regression models were used to analyze factors associated with diagnoses of syphilis, gonorrhea/chlamydia, and respective diagnoses classified as symptomatic and asymptomatic. If applicable, they included country-level screening rates.
Questions on STI diagnoses and sexual behaviors were answered by 156,018 (2010) and 125,837 (2017) participants. Between 2010 and 2017, overall diagnoses with gonorrhea/chlamydia and syphilis increased by 75% and 83% across countries. Increases were more pronounced for asymptomatic compared to symptomatic infections. The proportion of respondents screened and the frequency of screening grew considerably. Condomless anal intercourse with the last non-steady partner rose by 62%; self-reported partner numbers grew. Increased syphilis diagnoses were largely explained by behavioral changes (including more frequent screening). Gonorrhea/chlamydia increases were predominantly explained by more screening and a change in testing performance. A country variable representing the proportion of men screened for asymptomatic infection was positively associated with reporting symptomatic gonorrhea/chlamydia, but not syphilis.
The positive association of country-level screening rates with the proportion of symptomatic infections with gonorrhea/chlamydia may indicate a paradoxical effect of screening on incidence of symptomatic infections. Treatment of asymptomatic men might render them more susceptible to new infections, while spontaneous clearance may result in reduced susceptibility. Before expanding screening programs, evidence of the effects of screening and treatment is warranted.

**072**
**Paper-ID: 46657, O2**

Withdrawn.

**073**
**Paper-ID: 46551, O3**

**Partner notification in the case of an STI diagnosis: experiences and needs from the clients perspective**

Martina Schu, K. Enders
*FOGS GmbH*

**Introduction:** In the German government‘s strategy to contain HIV, HBV, HCV and other STI, special importance is given to partner notification (PN) or partner information (in german: PB) when it comes to “breaking chains of infection”. In order to be able to assess the situation in Germany more precisely and to identify the need for action, the Federal Ministry of Health commissioned an analysis of the situation on PB. This concerned partner notification in the context of anonymous testing for chlamydia, gonorrhea and syphilis. In the study conducted in 2019, the experiences and needs of clients in Germany were collected on a larger scale for the first time.

**Methods:** The study was based on a multi-stage survey and evaluation procedure:

1. Systematic literature review on research results, recommendations and guidelines concerning PB.
2. Qualitative study on the implementation of PB in Germany based on interviews with 45 practitioners in 16 test centres.
3. Investigation on clients perspective in the 16 test sites using questionnaire surveys (N = 721) and questionnaire-based interviews on site (N = 168).

**Results:** 30% of the interview partners said that they had been informed by sexual contacts in the past about being at risk of contracting an STI, among them mainly MSM. However, only 10% of the interview partners have received counselling on PB within the framework of test counselling. Most respondents want to be informed about an STI risk in any case, regardless of the channel (e. g. personal, phone, messenger). Almost all respondents would also inform sexual contacts themselves in case of infection, but less so with decreasing relationship intensity and depending on the presence of inhibiting factors. 80% of respondents would like advice on PB options, information material and in-depth counselling. It is important for them to be able to point out concrete help options—also for partners who do not have health insurance. The need for counselling is highest among younger people and first-time testers.
**Conclusion:** Partner notification concerns clients of anonymous testing sites, many feel a sense of responsibility and want to inform their sexual partners. But many find this difficult; there is a clear need for more (advice on) PB, possibly even for provider referral PB. Any kind of information material would also be welcome. The analysis shows typical barriers, identifies initial proposals for solutions and indicates the need for further action and research.

**074**
**Paper-ID: 46958, O4**

**SEXUELL GESUND—a free app for young people created by the HIV/AIDS Consultation Center Styria, Austria**

Flora Hutz, Eva Fellner
*AIDS-Hilfe Steiermark*

**Introduction:** Young people searching for information about sexuality prefer the internet. The quality of information found there, however, is contradictory or simply bad—this is why we decided to develop a high-quality digital source of information for young people.
**Methods:** We developed a needs assessment in order to find out if a new information platform would be received well by young people. We conducted a literature research, complimented with a re-analysis of our own existing recent studies, and we carried out a survey with young people (n = 149).
**Results:** The essential findings of the needs assessment are as follows:

- There was no app containing information about sexual wellbeing in the German-speaking countries.
- 74% of the surveyed youngsters were interested in trying out the app.
- Young people showed interest in a variety of topics, clearly structured and explained, supplemented by photos and contact information of consultation centres.

The app was developed together with the eHealth Institute at the University of Applied Sciences Joanneum. It is optimized for the use on smartphones and can be used without access to the internet. Topics such as body awareness, sex and the law, contraception, HIV and STI

are presented via short videos and complemented with a profound knowledge base. Helpful links, emergency numbers and contact information of consultancy centres can also be found on the app.
**Conclusions:** The app has been available since February 2020 and spread by social and communal networks and via peer groups. Due to the restrictions of this year, we have not been able to use the app in numerous workshops yet. Nevertheless, it has been downloaded 721 times already, which definitely speaks for its good acceptance within the target audience.
The app was honoured with the Styrian "SALUS 2020" award in the area of health promotion.

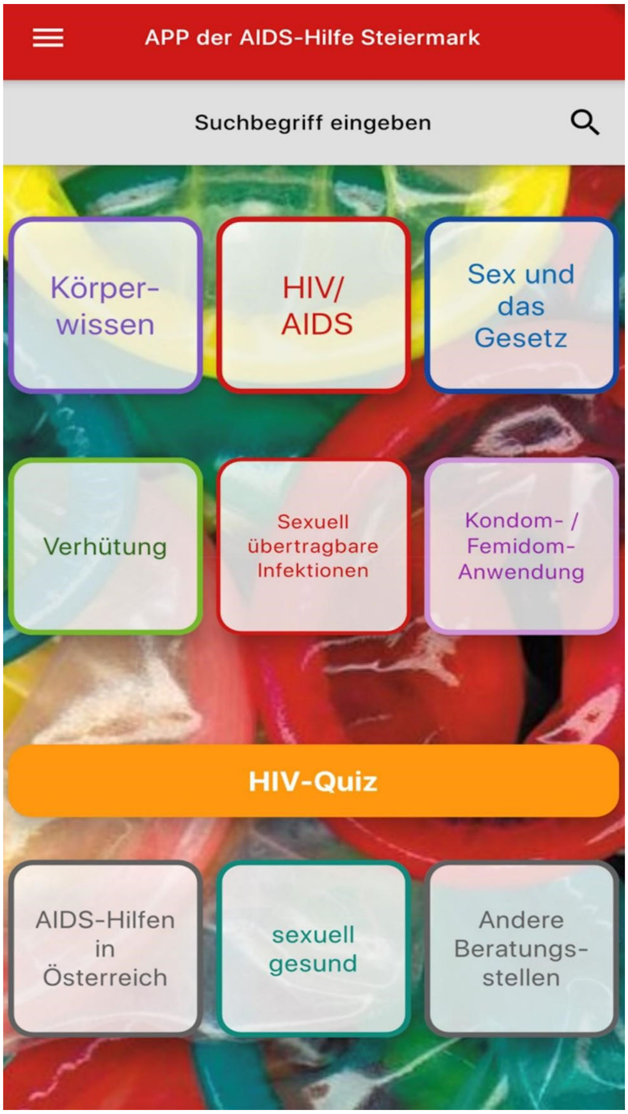

**075**
**Paper-ID: 49999, OE5**

**How present is knowledge about STIs in the general population? Results of the First Nationwide Representative German Health and Sexuality Survey—GeSiD**

Arne Dekker
*Institut für Sexualforschung, Sexualmedizin und Forensische Psychychiatrie, Universitätsklinikum Hamburg-Eppendorf*

**Objectives:** The aim of this presentation is to analyze the awareness of and knowledge about nine STI (HIV/Aids; syphilis; gonorrhoe; hepatitis B; genital herpes; pubic lice; chlamydia; genital warts and trichomonadida) in the German population. To do so, the correlations of the level of information with socio-demographic variables (age, gender, education, migration, regional social deprivation as an external criterion), sexuality-related characteristics (number of sexual partners, sexual orientation, incidence of STI), and subjective satisfaction are examined.
**Methods:** The "German Health and Sexuality Survey (GeSiD)" has collected comprehensive representative representative data on sexual and relationship behavior in Germany, including data on knowledge about STIs. face-to-face interview were conducted with a two-step random sample of 4,955 persons aged 18–75 years. As a selection procedure, a two-step random sample was collected. The participation rate was 30.2% (AAPOR RR4).
**Results:** Knowledge about HIV/AIDS was widespread in all age groups, but other sexually transmitted infections (STI) were significantly less known. Older people and respondents with a low level of education were particularly poorly informed. Among the sexuality-related factors, the number of sexual partners has the strongest effect so far: a higher number of sexual partners is related to a better level of knowledge. In addition, persons who do not describe their sexual orientation as heterosexual as well as persons who have had a STI before were well informed.
**Conclusion:** Heterosexual adults in Germany are insufficiently informed about the risks of STI. Therefore, target group specific efforts are needed to improve knowledge about STI in order to improve the utilization of prevention programs among socially disadvantaged groups.

**076**
**Paper-ID: 46622, P1**

**Assessing HIV, hepatitis B and C, and syphilis among people who inject drugs in Germany—pilot study for a periodical national monitoring system**

Gyde Steffen, Amrei Krings, Ruth Zimmermann
*Robert Koch Institut (RKI)*

**Introduction:** People who inject drugs (PWID) are at high risk of blood-borne and sexually transmitted infections.
Monitoring of nationwide and regional infection point prevalence, risk factors and trends among PWID can inform public health response for targeted prevention and control measures in order to support the HIV/Hepatitis/STI elimination process in Germany.
This pilot study aims to develop, apply and evaluate a periodical monitoring system in two federal states in Germany for future data collection.
**Methods:** Seven hundred people who ever injected blood, aged 16+ years will be recruited by convenience sampling via low threshold drug services or opioid substitution treatment doctors in Berlin and Bavaria. Sociodemographic, behavioural data and dried blood spots (DBS) from capillary blood will be collected from participants. DBS will be tested for HIV, hepatitis B and C (HBV; HCV), and syphilis. Data collection is planned during routine services of the participating facility.
Participating facilities will be allocated to different study arms regarding individual return of results and survey: (1) testing without result return but referral to routine testing of the facility vs. testing without result return but point-of-care testing for HIV and HCV vs. testing with result return through the facility, and (2) survey by self-filling questionnaire vs. interview-assisted questionnaire.

The whole data collection process including the different study arms, language mediation via phone and online training tools for the facilities will be evaluated for feasibility and acceptance during and after the data collection period by surveys and discussion rounds with facilities and participants.
**Conclusion:** The results will be used to assess the reached population, to give a first estimate of the prevalence of HIV, HBV, HCV and syphilis among PWID in Berlin and Bavaria and to roll out a feasible and approved study design for a nationwide periodical monitoring system among PWID.

**077**
**Paper-ID: 46851, P2**

### Use of Online Risk Test (ORT) an anonymous web-based quiz to predict infection risk for sexually transmitted infections: a retrospective analysis

Anja Potthoff[1,2*], Adriane Skaletz-Rorowski[1,2*], Sandeep Nambiar[1,2], Arne Kayser[1,3], Andre Kasper[1,2], Norbert Brockmeyer[1,2]
*[1]Walk In Ruhr (WIR), Center for Sexual Health and Medicine, Bochum, Germany; [2]Interdisciplinary Immunological Outpatient Clinic, Center for Sexual Health and Medicine, Department of Dermatology, Venereology and Allergology, Ruhr Universität Bochum, Germany; [3]Aidshilfe (Aids Service Organization Bochum) e. V., Germany; *Both authors contributed equally to this work*

**Introduction:** Sexually active individuals may want to anonymously evaluate for themselves as to whether they are at increased risk for having a sexually transmitted infection (STI) and make a decision about what to do next. Our goal was to develop and evaluate a simple self-administerable web-based quiz for participants to determine their risk for HIV/STI.
**Methods:** As part of the web-based Online Risk Test/ORT (https://risikotest.wir-ruhr.de) hosted by WIR—Walk In Ruhr Bochum since December 2017, a total of 10,668 users voluntarily took a 25-item risk quiz online. The risk quiz included 25-questions about sociodemographic data, sexuality, various sexual risk behaviour, prior and current STI testing and STI results and PrEP-usage. Data was then stratified by both gender (male, female and transgender) and sexuality (Heterosexual male/female, MSM, WSW) and a retrospective analysis was performed to assess category-specific risk of sexual behavior, STI testing and STI prevalence.
**Results:** Of the 10,668 participants 84.5% were aged 18–39, 7.5% < 18 and 8.1% > 40. 53.1% were male, 46.3% were female and 0.6% transgender. 12.5% were men who have sex with men (MSM), 5.1% were women who have sex with women (WSM), 40.7% were heterosexual men and 41.7% were heterosexual women. Among these 24.9% had > 3 sexual partners, 67.8% engaged in condomless sex, > 60% have never tested for HIV/STI and only 0.4% reported PrEP usage. 0.1% of the cohort was HIV-positive and 9% had prior non-HIV STI of which 1.4% had it multiple times. Among those who had prior STI 60.2% underwent treatment together with their partners, 33.5% patient alone, 6.2% had no treatment and only 66.5% had a test-of-cure.
**Conclusion:** The anonymous HIV/STI online risk test helped to improve prevention and counseling services at the WIR -Walk In Ruhr-Bochum.

**078**
**Paper-ID: 46918, P3**

### High number of asymptomatic sexually transmitted infections among Austrian PrEP users

David Chromy[1], Katharina Grabmeier-Pfistershammer[1], Michael Skoll[1], Veronique Touzeau-Roemer[1], Alexandra Geusau[1], Thomas Reiberger[2], Florian Mayer[3], Robert Strassl[3], Birgit Willinger[4], Armin Rieger[1], Wolfgang Bauer[1]
*[1]Medizinische Universität Wien, Universitätsklinik für Dermatologie; [2]Medizinische Universität Wien, Universitätsklinik für Innere Medizin III, Abteilung für Gastroenterologie und Hepatologie; [3]Medizinische Universität Wien, Klinisches Institut für Labormedizin, Abteilung für Klinische Virologie; [4]Medizinische Universität Wien, Klinisches Institut für Labormedizin, Abteilung für Klinische Mikrobiologie*

**Introduction:** Men who have sex with men (MSM) in Austria represent the population at highest risk for HIV acquisition due to common practice of casual dating, condomless anal intercourse (CAI) and high-risk sex-practices. While pre-exposure prophylaxis for HIV (PrEP) is a well-established and effective prevention strategy against HIV, multiple reports indicate a rise in sexually transmitted infections (STI) due to 'risk compensation'. Recently, we initiated a prospective observational registry study for systematic and longitudinal characterization of Austrian PrEP users.
**Methods:** All individuals presenting for the initiation of PrEP or already using PrEP attending our STI- or HIVoutpatient ward at the Vienna General Hospital since July-2020 are screened for inclusion. Indication for PrEP and visit intervals (every 3 months) are based on the European AIDS Clinical Society Guidelines. Every visit includes a blood draw and swab-collection from the pharyngeal-, urethral and anal mucosa for *Neisseria gonorrhoeae*, *Chlamydia trachomatis* and *Mycoplasma genitalium* testing. Annually, local HPV-testing, anal cytology and stool testing for *Helicobacter pylori*, parasites and a BioFire®-GI panel analysis is performed.
**Results:** Fourty-four patients (all MSM) were included since July-2020 with a median age of 34.7 years. The median number of sex-partners during the last 12 months was n = 15 (range 1–100), 38/44 (86%) had engaged in CAI and 16/44 (36%) reported sexualized drug-use. At inclusion, asymptomatic infection with *N. gonorrhoea*, *C. trachomatis*, *M. genitalium*, *T. pallidum* and *H. pylori* were present in 5/41 (12%), 3/44 (7%), 8/41 (20%), 1/44 (2%) and 5/15 (33%), respectively. Accordingly, 36% of all individuals had at least one infection. Thirteen patients (30%) had a history of syphilis. High-risk HPV-subtypes were found in 23/44 (52%) including 8/23 (35%) with high-grade squamous intraepithelial lesions.
**Conclusions:** Preliminary analyses indicate a high number of asymptomatic STIs among Austrian PrEP users. While PrEP is an important approach to avert HIV transmission, close STI-monitoring seems warranted in this high-risk population.

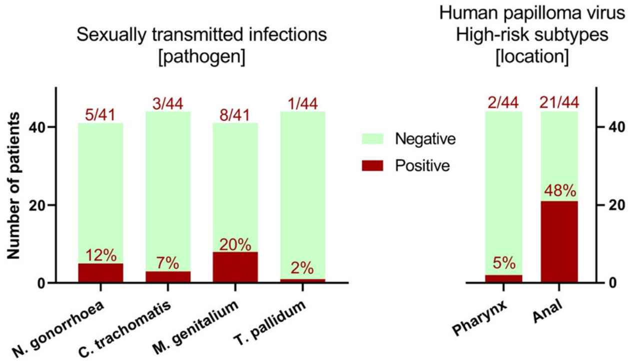

**079**
**Paper-ID: 46959, P4**

### Antimicrobial resistance of *Mycoplasma genitalium* and treatment outcome in men attending a STI and HIV center in Dresden

Petra Spornraft-Ragaller[1], Roger Dumke[2]
*[1]Klinik und Poliklinik für Dermatologie, Universitätsklinikum Carl Gustav Carus an der Technischen Universität Dresden; [2]Institut für Medizinische Mikrobiologie und Virologie an der Medizinischen Fakultät der TU Dresden*

**Introduction:** *M. genitalium* is a frequent cause of sexually transmitted infections (STIs), particularly in men who have sex with men (MSM). Although often asymptomatic, treatment is hampered by growing antimicrobial resistance. We investigated antimicrobial resistance of our recent strains of *M. genitalium* and the outcome of resistance guided therapy.
**Methods:** From 1.1.2019–15.11.2020 71 M. genitalium (M.g.) positive swabs of 53 men were analyzed regarding mutations associated with macrolide (23S rRNA) and fluoroquinolone (parC gene) resistance. Most were MSM (n = 47) with HIV-infection (n = 40). Rectal swabs and urine were obtained on screening, urethral swabs mainly in the presence of symptoms or other STIs.
**Results:** Of 71 strains, 50 exhibited antimicrobial resistance (70%); 39 against macrolides (55% of 71), 3 against quinolones (4%) and 8 (11%) against both macrolides and quinolones. Thus, macrolide resistance occurred in 47 (66%) and quinolone resistance in 11 (15.5%) strains. In 31 patients a test of cure (TOC) was available. Of 13 patients exhibiting antibiotic-sensitive M.g. strains 12 responded to therapy with Azithromycin (AZM) and 1 to Doxycycline. Of 16 patients with macrolide resistant M.g. strains, 10 responded to Moxifloxacin and 3 to Doxycycline. In 1 patient with an unusual makrolide resistance mutation (2084 C → T), AZM still seemed to be effective; in 2 patients antibiotic therapy failed. Alltogether, in 14 of 16 patients with makrolide resistant M.g. TOC was negative (87.5%). In 2 patients, M.g. was resistant against macrolides and quinolones, one of whom had urethritis. In both TOC cleared after treatment with Doxycycline, as did symptoms of urethritis.
**Conclusion:** Antimicrobial resistance of Mycoplasma genitalium was high with 66% against macrolides and 15% against fluoroquinolones. However, resistance guided therapy seems to be effective in most cases and should be recommended, at least in the presence of symptoms. Even in multiresistant strains, Doxycycline can be tried.

**080**
**Paper-ID: 46849, P5**

### Efficacy of syphilis treatment in HIV-infected patients

Justus Vehring[1], Stefanie Sammet[1], Evelyn Heintschel von Heinegg[2], Dirk Schadendorf[1], Stefan Esser[1]
*[1]Department of Dermatology and Venerology, University Hospital Essen, Essen, Germany; [2]Institute of Clinical Microbiology, University Hospital Essen, Essen, Germany*

**Introduction:** The incidence of syphilis coinfections especially in HIV-positive (HIV+) men having sex with men (MSM) is still high in Germany. Only a few prospective randomized studies investigate the efficacy of syphilis treatment reporting failure rates up to 30%.
**Methods:** This monocentric, retrospective analysis included HIV/syphilis coinfected patients treated by the venerologic out-patient clinic at the University Hospital Essen in Germany with completely documented follow-ups including clinical and serologic syphilis course controlled of at least 12 months after new syphilis diagnosis since January 2013 until April 2018. Complete treatment success was defined as disappearance of syphilis associated symptoms, at least four-fold decrease in nontreponemal test titres (RPR or VDRL) and IgM negativity during a one year follow-up.
**Results:** 190 HIV+ patients (98.9% male, 96.3% MSM) with 270 new syphilis cases during the observation period and a mean age of 42.1 ± 11.0 years at baseline presented with primary (11.9%) or secondary syphilis (29.2%), early latent syphilis (48.5%) or late latent syphilis (9.3%), or neurosyphilis (1.1%). Diagnosis of the first syphilis was provided in 25.9% of the cases. 55.6% of all cases were treated with a single i.m. injection of 2.4 million IU Benzathine Benzylpenicillin and 23.3% with three injections over three weeks, 6.7% with Doxycyclin 2 × 100 mg/d p.o. for two weeks, 4.8% with Ceftriaxone 1 × 2 g/d and 1,5% with Penicillin G 3 × 10million IU/d, both i.v. for 14 days. During the 1 year follow-up after syphilis treatment in 98.5% healing of the symptoms was documented, in 91.9% at least four-fold decreases of non-treponemal tests and in 74.8% IgM negativity. Only 8.1% of the cases remain in serofast status. According to the criteria complete serological and clinical treatment success was reached in 72.6% of the cases after one year.
**Conclusion:** Syphilis therapies were clinically and serologically highly effective in HIV+ patients with early syphilis.

Figure 1:
Kaplan-Meier curve in response of at least four-fould decrease in non-treponemal test titres (VDRL) during the observation period.

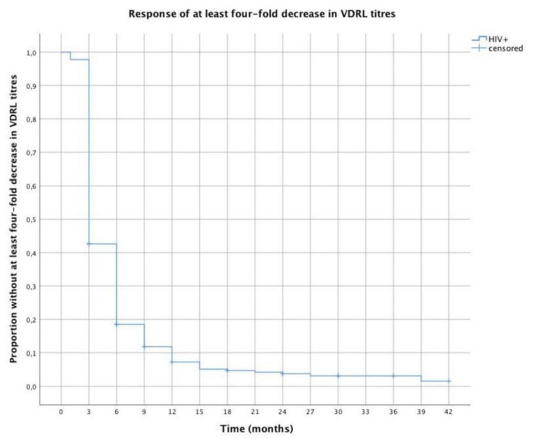

**081**
**Paper-ID: 46553, P6**

### Partner notification in the case of an STI diagnosis: situation and needs from the perspective of anonymous testing centres

Martina Schu, K. Enders
*FOGS GmbH*

**Introduction:** Partner notification (PN) is an important STI control measure worldwide, but with significant differences between countries. WHO, UN-AIDS and IUSTI recommend addressing PN oder Partner Information (in german: PB) systematically, but on a voluntary basis. In the German government's strategy to contain HIV, HBV, HCV and other STI, PB is given special importance in order to "break chains of infection". To assess the situation in Germany more precisely and to identify the need for action, the BMG commissioned an analysis of the situation on PB in anonymous testing centres for chlamydia, gonorrhoea and syphilis.
**Methods:** Systematic research on evidence and guidelines, questionnaire surveys and qualitative individual and group interviews with professionals from 16 anonymous testing centres at eight locations as well as questionnaire surveys and interviews with clients were implemented.

**Results:** In Germany, medical guidelines exist across STI and STI-specific, which recommend—mostly concisely—PB. Guidelines are also available from the public health service in North Rhine-Westphalia and from the DAH; PB plays a minor role here as well. Professional standards for counelling on PB are not very common at anonymous testing centres. Test counselling often focuses on education about (re)infection risks. Active counselling on the various possibilities of PB as well as motivation and support for this matter are rare. In addition to different competences, divergent attitudes are also evident in the public health service and the NGO: In comparison with the ÖGD only half as many professionals of the NGO want to support clients in notifying their sexual partners themselves. Professionals are particularly sceptic concerning provider referral PB.

Professionals vote for more counselling on PB, want more structured approaches as well as professional training and helpful procedures and tools. They consider it important to offer treatment to people who test positive—even without health insurance.

**Conclusion:** Counselling on PB is implemented very differently, there are diverging attitudes and hardly any rules, procedures or tools. Specific competences for PB hardly exist—as well as further training on helpful procedures. The study shows concrete needs for improvement.

**082**
**Paper-ID: 46912, P7**

### Lymphogranuloma venereum in a HIV-positive patient with acute proctitis

Isabel Lange[1], Michael Baier[2], Katja Adler[3], Benjamin Schleenvoigt[1]
*[1]Institut für Infektionsmedizin und Krankenhaushygiene, Universitätsklinikum Jena; [2]Institut für Medizinische Mikrobiologie, Universitätsklinikum Jena; [3]Zentrum für ambulante Medizin, Uniklinikum Jena*

Lymphogranuloma venereum (LGV) is a sexually transmitted infection (STI) caused by invasive serovars L1-L3 of Chlamydia trachomatis. Several studies suggest an increasing trend since 2003 in Europe and hint at an ongoing epidemic, especially in HIV-positive men who have sex with men (MSM. This trend is also visible in Germany.

Recent numbers for all C. trachomatis infections from 2014 from the German institute for public health, Robert-Koch-Institute (RKI) showed a 10% prevalence in all men tested. The only federal state reporting chlamydia infections is Saxony. Here, a steady increase is visible with 26.3/100,000 in 2003 to 102/100,000 in 2012. Serovars were not specified, therefore the number of LGV is unknown.

Since 50% of C. trachomatis infections in men are asymptomatic, regular screening represents a necessary measure to make infections visible, provide suitable treatment and stop transmissions. Here we present a case of a HIV-positive 53-year old male patient with a rectal LGV infection with proctitis that was found coincidentally through an age-advised colorectal cancer screening. With the diagnosis of distal proctitis, a screening for C. trachomatis and Neisseria gonorrhoeae via rectal swab resulted in a positive polymerase-chain-reaction (PCR) for C. trachomatis DNA. A follow-up PCR for the major outer membrane protein (MOMP) was positive. Serovar L2 was found. The final diagnosis of LGV was established and therapy with doxycycline 100 mg, twice a day was prolonged to 21 days. The patient's symptoms resolved.

**Conclusion:** It is important to screen for LGV via MOMP-PCR in chlamydia cases, especially in MSM to prevent severe complications like intestinal fistulas that can lead to symptoms like chronic inflammatory bowel disease (CIBD). All federal states should report cases of C. trachomatis and determine serovars to elaborate data about chlamydia infections, stop transmissions and improve sexual health in Germany.

### Pediatrics, pregnancy

**083**
**Paper-ID: 46919, O1**

### Dynamics of body weight and BMI after change of therapy in HIV-positive children and adolescents—GEPIC

Manuel Küster[1], Marla Braun[1], Stefan Schöning1, Robin Kobbe[2], Bernd Buchholz[3], Cornelia Feiterna-Sperling[4], Ulrich Baumann[5], Ulf Schulze-Sturm[2], Christoph Königs[1], for the GEPIC study group
*[1]Goethe Universität Frankfurt am Main; [2]UKE Hamburg Eppendorf; [3]UMM Universitätsmedizin Mannheim; [4]Charité - Universitätsmedizin Berlin; [5]MHH Hannover*

**Introduction:** An increased of BMI after initiation of cART, in particular but not only among patients with lower baseline-BMI or as part of immune reconstitution, has been shown in cohort studies of adult PWH. Weight gain, originally associated with therapeutic success in PWH, is now associated with overweight in 50% of cases with an increased risk of secondary diseases. This seems to be more pronounced in association with Dolutegravir and Tenofovir-AF. Data on children and adolescents are not available.

**Methods:** The 'German Cohort of Children and Adolescents exposed or infected with HIV—GEPIC' collects clinical, immunological and virological data from HIV-positive children and adolescents living in Germany. Weight gain after a change of therapy was examined. Children and adolescents with CD4+ T-cell-baseline less than age-specific norm at the beginning of DTG-treatment were excluded to acknowledge weight gain as reversible catabolism.

**Results:** Study inclusion criteria were met by 176 children or adolescents and 571 therapy changes were evaluated. Antiretroviral regimen of 19 children and adolescents with normal count of CD4+ T-cells included DTG. After starting DTG, annual BMI increase of 1.92 over observation period of 15 months ($P < 0.001$) was observed. While baseline median BMI at start of DTG was 20.38, after 15 month it was 22.78. The antiretroviral regimen included Tenofovir-AF in 20 children and in this subgroup BMI gain of 0.84 annually ($P < 0.001$) was detected. For other antiretroviral substances there was no significant increase in median BMI. In the total cohort, 23% and 16% of the infected girls and boys were overweight (cf. German KIGG's study of healthy children and adolescents: 15%).

**Conclusion:** A significant weight gain was observed in children and adolescents starting a DTG- or TAF-containing in cART-regimen. The impact on increased body weight on morbidity and adherence especially in adolescence needs to be discussed.

**084**
**Paper-ID: 47324, O2**

### Success of antiretroviral therapy in HIV positive children and adolescents in relation to guideline adherence

Manuel Küster[1], Christoph Königs[1], Marla Braun[1], Robin Kobbe[2], Cornelia Feiterna-Sperling[3], Ulrich Baumann[4], Bernd Buchholz[5], Stefan Schöning[1], Ulf Schulze-Sturm[2], for the GEPIC Study Group

[1]Goethe Universitaet Frankfurt am Main; [2]UKE Hamburg-Eppendorf; [3]Charité-Universitätsmedizin Berlin; [4]Medizinische Hochschule Hannover (MHH); [5]UMM Universitätsmedizin Mannheim

**Background:** The Pediatric Working Group AIDS, a working group of the DAIG e.V., regularly publishes guidelines for antiretroviral therapy in children and adolescents. Data about therapy, its success and guideline adherence are still missing in Germany. The aim of this study was to evaluate a potential correlation between success of therapy and adherence to the guidelines.
**Methods:** We evaluated the guideline adherence in 143 children and adolescents in a period of time from 1999 to 2019 in the German Pediatric and Adolescents HIV Cohort (GEPIC). Guideline adherence was evaluated to the valid guideline at time of therapy initiation. Periods of guidelines were 1999–2001, 2002–2006, 2007–2011, 2012–2019. Afterwards the duration of the first prescribed therapy was determined and compared between the groups.
**Results:** 71% of the evaluated children received a therapy according to the guidelines. Children and adolescents treated according to current guidelines showed a longer duration of first-line regimens compared to those receiving a different regimen. When analyzing adherence to the current and the following guideline, adherence rose to 88%. The duration of first-line therapy did not differ for PI- or NNRTI-based regimens. The median duration of PI- or NNRTI-based therapy was 32.91 and 35.21 months, respectively (P = 0.57). There was no difference in the duration of therapy according to sex (P = 0.5).
**Conclusions:** A therapy according to the guidelines correlated with a longer duration of first-line therapy. Guidelines had a positive impact on success of therapy, whilst showing a need for more recent updates, as well as the need for fast implementation of guidelines to allow an optimal success of therapy.

### 085
### Paper-ID: 46703, O3

### HIV-testing and counseling during pregnancy—where do we stand in 2020?

Laila Cravat, Waltraut M. Merz[1], Kathrin van Bremen[2], Jan-Christian Wasmuth[2], Christoph Boesecke[2], Jürgen Rockstroh[2], Carolynne Schwarze-Zander[2], Brigitte Strizek[1]
[1]Universität Bonn, Universitätsfrauenklinik; [2]Universität Bonn, medizinische Klinik I

**Introduction:** Multimodal HIV transmission prophylaxis can reduce mother-to-child transmission to < 1%. In Germany counseling about the universal recommendation of HIV testing is mandatory, but the HIV test itself is performed on an opt-in basis. The aim of our study was to investigate the implementation of the mandatory counseling and the uptake of HIV testing in the Bonn patient population during pregnancy, as recommended in the German maternity guidelines.
**Methods:** In the period from June to October 2020, pregnant women at the University Women's Hospital Bonn were interviewed in the antenatal clinic and in the maternity ward with the help of an anonymous questionnaire about the counseling on HIV testing and its implementation during pregnancy. On the other hand, in the prepartum and postpartum wards, women´s maternity passports were reviewed with regard to the documentation of HIV counseling and the performance of the HIV test.
**Results:** A total of 291 questionnaires from pregnant women were analyzed. The median age of the women was 33 [IQR = 30;36]. The women were 94.2% Caucasian, 0.3% African, 1.4% American, and 4.1% Asian. None of the women had been diagnosed with HIV infection during this pregnancy. Counseling for HIV testing was recalled by 53% of the women, 14% could not recall counseling, and 32% indicated that no counseling had been provided. Documentation of counseling for HIV testing and performance of HIV testing was collected in 401 maternity records. In 8% of them (33/401), neither counseling for HIV testing nor test performance were documented. Documentation of counseling without documentation of test performance was present in 2.5% (10/401) of the maternity passports.
**Conclusion:** Despite the legal change regarding the HIV test counseling obligation during pregnancy and the offer of free testing, documentation of test performance is missing in every tenth maternity passport. Only about half of the women can recall having received counseling about the recommendation for HIV testing. Further measures are needed to optimize the reliability of counseling and testing and thus prevent potential HIV mother-to-child transmissions.

### 086
### Paper-ID: 46956, P1

### HIV-positive mothers and changing medical recommendations on infant feeding—an ethnographic approach

Lea Dickopf

**Introduction:** This research focuses on the struggles of being a good mother while facing stigmatization because of HIV. It gives insight on the individual strategies in handling prejudicial social expectations and the difficult relation of HIV-positive mothers and the medical field towards breastfeeding. It also explores motives regarding the choice of feeding method and the obstacles mothers face when trying to make an autonomous and informed decision.
**Methods:** 18 Interviews were conducted, transcribed and coded with a three-phase coding model. The theory building was methodologically guided by Charmaz 'Constructing Grounded Theory' (Charmaz 2014: Constructing Grounded Theory). I address a range of challenges of ethnographic research e.g. the power relations between interviewer and interviewee when dealing with discrimination and stigma. Collaborative texts and experience reports written by the interviewees are also part of the research.
**Results:** For HIV-positive women, motherhood can mean regaining agency over a body that was formerly only addressed as deficient and contagious. Motherhood can create a new impetus to fight against stigma, but it can also be a reason not to talk openly about one's infection, when mothers fear negative consequences for their child. Networking with other HIV-positive mothers is important, especially when mothers face different recommendations from different doctors, institutions or countries.
**Conclusion:** Just as motherhood itself, breastfeeding can be an important source of identity for HIV-positive mothers who fear that they are a not good enough person/woman/mother. The new DAIG guidelines 2020 call to support mothers who wish to breastfeed. Nevertheless, it needs to be ensured that these new recommendations reach the local information centers and medical practices. To eliminate the still existing uncertainties, we need an interdisciplinary European research that includes mother milk testing and the consideration of the emotional demands of mothers.

### 087
**Paper-ID: 46947 P2**

**Breastfeeding with HIV: striving for normality in motherhood**

Eva Simakoloyi, Ute Lange
*Hochschule für Gesundheit (hsg) Bochum*

**Introduction:** In the former guideline valid for Germany and Austria, HIV positive mothers were recommended to exclusively feed their newborns with formula milk. Despite the recommendations and expected challenges, some of the women decided to breastfeed for personal reasons. The aim of this study is to look at the subjective experiences of HIV positive women while breastfeeding, the influencing factors in the decision-making process and their related needs in the pre- and postnatal care.
**Methods:** A qualitative research design with three semi-structured interviews and content analysis was used. The sample included three HIV positive women with breastfeeding experiences from four weeks to ten months.
**Results:** The results of the study reveal that HIV positive women want to experience normality in motherhood through breastfeeding. In the decision-making process the women gather knowledge about breastfeeding mainly by themselves and often lack uptodate information from medical professionals. Access to appropriate midwifery care is a challenge. The care by medical professionals often leads to negative experiences in the breastfeeding period and ultimately affect the overall period of early motherhood.
**Conclusions:** The recently adapted guideline for positive women in Germany and Austria includes participatory decision-making as a recommendation. HIV positive women need unbiased counselling and care if they decided to breastfeed. Midwives and doctors should inform themselves on HIV and pregnancy and breastfeeding by participating in trainings and consulting uptodate literature to render more informed services. In the network of the helping system working with HIV positive women around childbirth, midwives can play a more prominent role by offering lactation consultancy in postnatal care. The availability of midwives specialised in care for positive women as well as their contact lists (local and national) at HIV specialised physicians, self-help associations, hospitals and gynaecologists are highly desirable.

### 088
**Paper-ID: 46561, P3**

**"Aids, Children and Families": a network focusing on the central topic of women and HIV in Lower Saxony, initiated by the Lower Saxony AIDS-Service-Organization (AHN)**

Ingrid Mumm[1], Christin Engelbrecht[2], Kerstin Göllner[3], Caroline Herberhold[4], Ellen Kiebacher[5], Marja Rathert[6], Matthias Stoll[7]
[1]*Your Health-Your Right, Positive Network for Children and Families;* [2]*Aidshilfe Niedersachsen, Landesverband e.V. (AHN);* [3]*Braunschweiger Aids-Hilfe e.V.;* [4]*Göttinger AIDS-Hilfe e.V;* [5]*AIDS-Hilfe Oldenburg e.V.;* [6]*Freelance consultant for intercultural health;* [7]*Medizinische Hochschule Hannover (MHH), Aidshilfe Niedersachsen, Landesverband e.V. (AHN)*

**Introduction:** Mother-to-child-transmissions (MTCT) in Germany has been low in the past decade: no cases of MTCT in 2019 (RKI-report 01/2020). Crucial for this trend is an increased access to HIV-testing programs during pregnancy and the subsequent initiation of antiretroviral therapy. Consequently, our networks focus shifted from HIV-positive children to women living with HIV (WLWHIV). Hence, "Aids, Children and Families" strives to expand anonymous and free of charge test offers for women outside of pregnancies and seeks to enhance a qualified transfer of knowledge about HIV/Aids and women, facilitated by exchange of information about interdisciplinary approaches, vaginal delivery, breastfeeding and gender-specific medical care options for WLWHIV throughout the whole region.
**Methods:** Established in 2013, the network has since been led by the coordination committee of the LowerSaxony AIDS-Service. Since 2016 its work has been increasingly focused on health care for WLWHIV, and granting them an easier access to important information for their respective living situation. Thus the network:

- Offers workshops on self-empowerment;
- Supports the demand for a non-discriminatory medical health care system;
- Represents the subject of women and HIV in national and international scientific meetings;
- Strengthens the cooperation and exchange between physicians, hospitals, health authorities and AIDS organizations in Lower Saxony;
- Synergistic cooperation with the specialized clinics and hospitals;
- Participation in and contribution to existing networks, work groups and committees;
- Certified conferences on "Gynecology and HIV", special trainings for midwives on "Pregnancy and HIV";
- In cooperation with the German AIDS-Service-Organization (DAH): Professionally edited brochures and close collaboration with their anti-discrimination commission.

Detailed data will be presented at DÖAK 2021.
**Conclusion:** Independent, informed decisions by WLWHIV who know and exercise their rights has had positive effects on their mental wellbeing. The approach of an empowering education in combination with respecting individual decisions made by women within existing guidelines has proven successful.

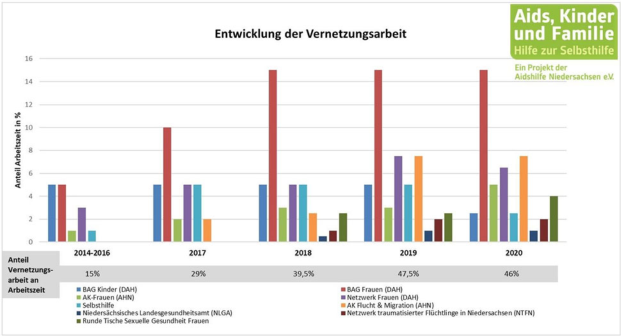

### Pre-exposure prophylaxis (PrEP)

### 089
**Paper-ID: 46632, O1**

**National Evaluation of the introduction of HIV pre-exposure prophylaxis as a service of the statutory health insurance in Germany (EvE-PrEP)**

Daniel Schmidt, Martin Friebe, Christian Kollan, Marie Merbach, Barbara Bartmeyer, Viviane Bremer
*Robert Koch Institut (RKI)*

**Introduction:** The introduction of the HIV pre-exposure prophylaxis (PrEP) as a service of the statutory health insurance (SHI) since

September 2019 is being monitored and evaluated within the framework of a research project financed by the Federal Ministry of Health. The effects of PrEP as a new prevention tool on the incidence of HIV and other sexually transmitted infections (STI) will be investigated.
**Methods:** The evaluation period is 01.01.2020–31.12.2020. HIV and STI surveillance data, various studies, prescription data and data from statutory health insurances are used. PrEP users are involved through surveys in several studies integrated in EvE-PrEP. PrEP use in HIV centres is being investigated. In a participative approach interest groups of PrEP users are involved through a community advisory board.
**Results:** Consortium partners are the German Association of Physicians Specializing in HIV Care (dagnä), the University Duisburg-Essen, the University Bonn, the University Bremen and several health insurances. The project lead is at the Robert Koch Institute (RKI). The community advisory board consists of representatives from different areas such as male and female sex work, PrEP users, African migrants and the German AIDS Service Organization. A software tool was developed at the RKI to anonymously monitor PrEP use at HIV centres in collaboration with the dagnä. In order to acknowledge the complexity of the research questions EvE-PrEP works with a multi- and interdisciplinary approach in a collaboration of various partners, studies and the participation of PrEP related communities (Fig. 1).
In March 2021 more results will be presented.
**Conclusions:** The introduction of PrEP as a service of the SHI in Germany offers potential to decrease new HIV infections. Questions remain regarding the influence of PrEP use on other STI as well as who may benefit from PrEP besides MSM. The evaluation project EvE-PrEP will investigate these questions.

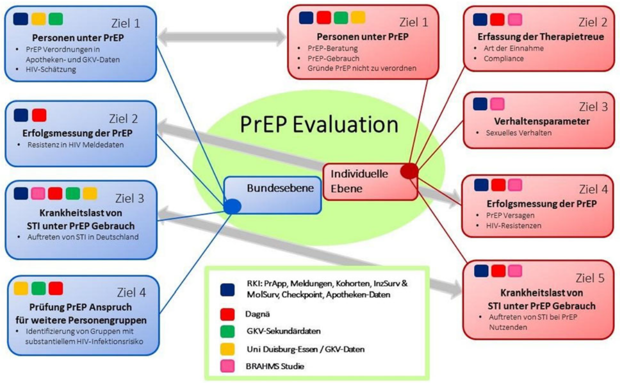

### 090
### Paper-ID: 46654, O2

### Estimating the number of HIV pre-exposure prophylaxis (PrEP) users among men having sex with men (MSM) in Germany, 2020

Ulrich Marcus, Daniel Schmidt, Susanne B. Schink, Uwe Koppe
*Robert Koch Institut*

Data on PrEP use in Germany is not collected systematically, and hence the number of PrEP users among men having sex with men (MSM) in Germany is unknown.
We estimated the number of PrEP users using drug prescription data provided by Insight Health, information on on-demand/intermittent PrEP use from the PrApp study, and the number of profiles on the gay dating platform ROMEO® indicating PrEP use, based on assumptions on the proportion of on-demand/intermittent PrEP users and the average number of PrEP pills used. We used two scenarios to estimate the number of PrEP users in Germany in 06/2020.
In 2020, we estimated that 10,788 quarterly prescriptions for PrEP were filled. For scenario 1, we extrapolated the number of PrEP users projecting that 20% of the users use PrEP on-demand as found in the PrApp study. We assumed that one monthly prescription fulfills the average needs of 3 on-demand users. In addition, we assumed that 20% of the PrEP users take PrEP on private prescription and from informal sources. Thus, we estimated an overall number of 15,600 MSM in this scenario.
In scenario 2, we use the number of PrEP users as stated in ROMEO® profiles. Since we know from the PrApp study that approximately 30% of all users do not declare PrEP use in their online profile, we extrapolated that there are an overall of 22,300 PrEP users. Distributions of PrEP users from scenario 1 and 2 by federal state were highly correlated, except for a higher proportion of PrEP profiles on ROMEO® in Berlin compared to the proportion of PrApp survey participants using PrEP.
In 06/2020, we estimated between 15,600 and 22,300 PrEP users in Germany. The largest discrepancy regarding the proportion of PrEP users between the two scenarios was observed for Berlin.

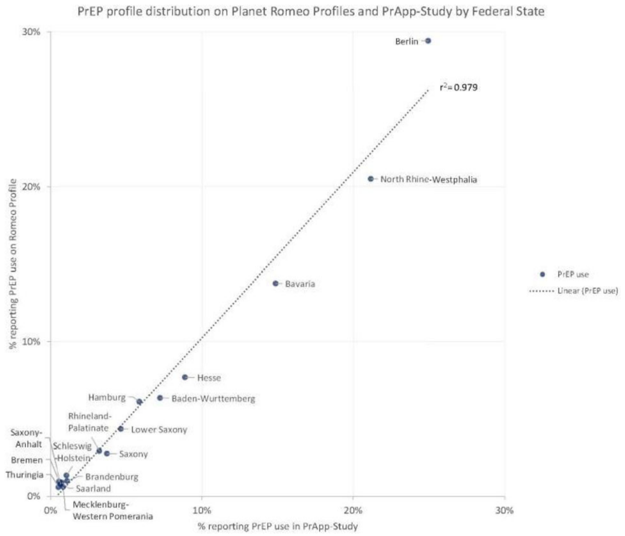

### 091
### Paper-ID: 46970, P1

### DISCOVER STUDY for HIV pre-exposure prophylaxis (PrEP): no evidence of risk compensation in participants taking F/TDF or F/TAF for PrEP through 96 weeks

Ivanka Krznaric[1], Peter Shalit[2], Karam Mounzer[3], Laura Salazar[4], Yongwu Shao[5], Pamela Wong[5], Ramin Ebrahimi[5], Moupali Das[5], Diana M. Brainard[5], Christoph C. Carter[5], Jason Hindman[5], Patrick Philibert[6], Colm Kerr[7]
[1]*Praxis an der Kulturbrauerei;* [2]*Peter Shalit MD and Associates;* [3]*Perelman School of Medicine, University of Pennsylvania;* [4]*Hoag Medical Group;* [5]*Gilead Sciences Inc.;* [6]*Hôpital Européen;* [7]*Trinity College Dublin*

**Introduction:** In DISCOVER, emtricitabine plus tenofovir alafenamide (F/TAF) was non-inferior to F/tenofovir disoproxil fumarate (TDF) for prevention of HIV infection in participants who reported sexual behavior associated with HIV acquisition risk. Here, we report the baseline prevalence and longitudinal trends in HIV sexual risk behaviors during the DISCOVER trial, and assess for risk compensation, defined as an increase in risk behavior due to a reduction in perceived risk.

**Methods:** DISCOVER is an ongoing multi-center randomized controlled trial in which 5,335 men who have sex with men (MSM) and transgender women were randomized 1:1 and received F/TAF or F/TDF for PrEP. The primary endpoint occurred when half of participants reached 96 weeks of followup. Sexual behavior was assessed by Computer-Assisted Self-Interview questionnaire, including number of Sex Partners, Condomless Insertive Anal Sex (CIAS) Partners, and Condomless Receptive Anal Sex (CRAS) partners. Self-reported sexual behaviors from baseline through the primary endpoint were assessed using descriptive statistics.
**Results:** Between Sep 2016-June 2017 participants were randomized and received once-daily blinded tablets of F/TAF (n = 2694) or F/TDF (n = 2693) plus matched placebo. Baseline demographic, clinical, and risk behavior were balanced between arms. The median age was 34 years, 474 (9%) were black, and 1318 (24%) were of Hispanic or Latinx ethnicity. Most participants (91%) self-identified as gay; 385 (7%) as bisexual, 41 (1%) as heterosexual, and 71 (1%) as trans women. There were no longitudinal changes in the mean number of Sex Partners, Condomless Insertive Anal Sex Partners and Condomless Receptive Anal Sex Partners (Figure). These sexual behavior findings mirror the lack of change in STI rates observed in participants throughout the same timeframe (data not shown).
**Conclusions:** In DISCOVER, participants reported a stable number of total and condomless sexual partners through 96 weeks. These data suggest that risk compensation did not occur in DISCOVER.

**Figure.** Longitudinal trends in sexual behaviors in DISCOVER.

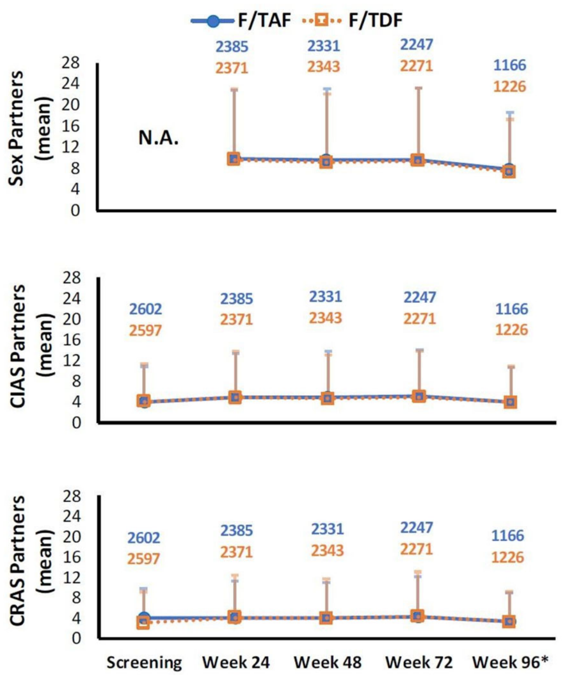

* Primary endpoint occurred when 50% of participants reached 96 weeks of follow-up

### 092
### Paper-ID: 46850, P2

### An innovative intersectoral collaborative model for HIV-pre-exposure prophylaxis (PrEP)-care in Germany

Patrick Ahaus[1,2], Adriane Skaletz-Rorowski[1,2], Arne Kayser[2,3], Janet Wach[2,3], Sandeep Nambiar[1,2],
Norbert H. Brockmeyer[1,2], Anja Potthoff[1,2]
[1]*Interdisciplinary Immunological Outpatient Clinic, Center for Sexual Health and Medicine, Department of Dermatology, Venereology and Allergology, Ruhr Universität Bochum, Germany;* [2]*Walk In Ruhr (WIR), Center for Sexual Health and Medicine, Bochum, Germany;* [3]*Aidshilfe (Aids Service Organization Bochum) e. V., Germany;* [4]*Public Health Department, Bochum, Germany*

**Objective:** The Walk In Ruhr (WIR)—Centre for Sexual Health and Medicine is an innovative intersectoral collaborative model for PrEP care in Germany. The current study describes the characteristics of PrEP users at the WIR, the prevalence of adverse drug reactions, the impact of PrEP use on sexual risk behaviour and the incidence of sexually transmitted infections (STI).
**Methods:** The study included 139 subjects who initiated PrEP between 10/2017 and 12/2018. The subjects were queried using an in house-developed questionnaire on their socio-economic and demographic backgrounds and sexual risk behaviour. In addition, clinical and laboratory examinations were performed.
**Results:** PrEP users had an average age of 38 years, 99.6% of them were men who had sex with men (MSM) and were characterized by a high educational status and were mostly well employed. Post PrEP initiation, nausea/vomiting, gastrointestinal symptoms and headache were the most commonly reported adverse drug reactions, particularly in the initial stages of PrEP use. Among PrEP users sexual risk behaviour increased significantly as evidenced by reduced condom use and increased number of sexual partners. During the course of this study, 148 instances of STI were reported in 68 subjects. None of the participants were infected with HIV.
**Conclusion:** The current study is a first of its kind in Germany to evaluate a PrEP-user cohort after the approval of PrEP but before the costs began to be covered by statutory health insurance. An intersectoral collaborative model for PrEP care such as ours is not only able to ensure uncomplicated access to PrEP prescriptions for high-risk individuals but also guarantees optimal support during the course of their PrEP uptake.

### 093
### Paper-ID: 46848, P3

### HIV pre-exposure prophylaxis (PrEP) during the COVID-19 pandemic

Mona Uhrmacher[1], Patrick Ahaus[2], Adriane Skaletz-Rorowski[1,2], Arne Kayser[2,3], Sandeep Nambiar[1,2], Norbert H. Brockmeyer[1,2], Anja Potthoff[1,2]
[1]*Interdisciplinary Immunological Outpatient Clinic, Center for Sexual Health and Medicine, Department of Dermatology, Venereology and Allergology, Ruhr Universität Bochum, Germany;* [2]*Walk In Ruhr (WIR), Center for Sexual Health and Medicine, Bochum, Germany;* [3]*Aidshilfe (Aids Service Organization Bochum) e. V., Germany*

**Introduction:** At the beginning of COVID 19 pandemic (3/2020), public access to many health facilities were notably reduced and all non-urgent patient appointments were cancelled. The impact of these as well as the contact restriction measures needs to be surveyed and studied thoroughly. At the Walk In Ruhr—Centre for Sexual Health and Medicine, an innovative intersectoral collaborative model for PrEP care in Germany, more than 500 PrEP users are being supported since 2016.
**Methods:** This survey includes 139 subjects who initiated PrEP between 09/2019 and 03/2020 and whose costs are covered by statutory health insurance. These subjects were queried using an in house-developed questionnaire on their socio-economic and demographic backgrounds, sexual risk behaviour and for impact of COVID 19 pandemic. In addition, clinical and laboratory examinations were performed.
**Results:** PrEP users had an average age of 34 years, 99.6% of them were men who had sex with men (MSM). Although care of PrEP users at the WIR remained uninterrupted and follow-up appointments were encouraged using a recall system, we observed that 40 of the PrEP users discontinued PrEP appointments from 3/2020 to 11/2020. Currently, the survey is ongoing and its evaluation will be presented.
**Conclusions:** The COVID 19 pandemic has had a strong negative impact on PrEP uptake in Germany. However, it remains to be ascertained whether such a decrease in PrEP uptake has subsequently impacted HIV prevention.

### 094
### Paper-ID: 46557, P4

### Harm reduction strategies and pre-exposure-prophylaxis (PrEP) among methamphetamine using men who have sex with men

Henrike Schecke
*LVR-Klinikum Essen*

**Introduction:** Men who have sex with men (MSM) are a vulnerable subgroup for problems with substance use. Substance use, including crystal methamphetamine, in sexual settings ("chemsex"), has been an issue of concern in some MSM communities. Substance use in sexual settings is correlated with sexual practices associated with the acquisition and transmission of sexually transmitted infections, including HIV and hepatitis C. Research on Pre-Exposure-Prophylaxis (PrEP) and other harm reduction strategies among methamphetamine using MSM is limited.
**Methods:** The analysis refers to a subset of participants from the German Chemsex Survey, an MSMcommunity recruited, self-completed online survey with a self-selected convenience sample. Participants who used crystal methamphetamine for sex (n = 130) were compared to participants who did not use drugs for sex (n = 177). The comprehensive survey comprised 420 different items considering recreational substance use, substance use in sexual settings, harm reduction strategies, mental health, sexual transmitted infections, and mental health care service utilization.
**Results:** A total of 1,583 men started the survey; 1,050 participants provided information on substance use. Twenty-seven percent of participants used crystal methamphetamine in the last 12 months, and of those, 89% used methamphetamine in a sexual setting and 50% reported injecting methamphetamine. Regarding mental health and HIV-related aspects, participants who reported methamphetamine use for sex were more likely to report symptoms of major depression, being HIV positive, and taking HIV pre-exposure prophylaxis (PrEP) than participants who did not report methamphetamine use. The vast majority (80%) of participants who reported methamphetamine use for sex reported a range of drug- and sex-related harm reduction practices.
**Conclusion:** MSM who used methamphetamine for sexual purposes seemed to be aware of potential health risks associated with their substance use and utilized harm reduction strategies and biomedical HIV prevention strategies like PrEP.

### 095
### Paper-ID: 47278, P5

### HIV seroconversions on PrEP in a major inner-city clinic population

Hans-Jürgen Stellbrink, Michael Sabranski, Thomas Buhk, Knud Schewe
*ICH Hamburg*

**Introduction:** TDF/FTC PrEP effectively prevents HIV infection, but failures have been described. We wanted to understand the characteristics of PrEP failures in our population.
**Methods:** HIV seroconversions in subjects on TDF/FTC PrEP, their pattern of PrEP use, interruptions, errors in drug intake, and missed visits were analyzed. Subjects were included if a minimum of one TDF/FTC prescription and one on-PrEP HIV test were documented.
**Results:** Among 901 TDF/FTC PrEP users (intended continuous use: 764, on demand use: 138) until 12/2020, seroconversion occurred in 3 MSM. Patients 2 and 3 reported clinical symptoms preceding diagnosis, patient 1 was asymptomatic, with persistent incomplete seroconversion after 6 months on ART. Patients 2 and 3 had missed a routine visit between January and September/June 2020 before HIV diagnosis. On subsequent ART, two patients reached HIV RNA $< 50$ c/ml; patient 2 still had 360 c/ml after 28 days.
**Conclusions:** HIV seroconversion on PrEP was very rare. One patient probably became infected before PrEP, one used OD PrEP irregularly, and one seroconverted on continuous PrEP despite high adherence. Seroconversion may occur with few symptoms and a corresponding low viral load as in patient 1, indicating incomplete virus suppression during seroconversion. Genotypic resistance to FTC (M184V) occurred in patient 3 only. His diagnosis at a late Fiebig stage indicates FTC selective pressure for some time before HIV diagnosis.
These cases demonstrate the importance of early monitoring to exclude HIV infection before PrEP (patient 1), of avoiding incorrect PrEP use (patient 2), and early diagnosis of failure on continuous PrEP in order to minimize the risk of resistance (patient 3).
Of note, patients 2 and 3 had missed a routine visit during the SARS-CoV-2 epidemic and lock-down, indicating that contact reductions might have contributed to insufficient PrEP monitoring, reduced or irregular intake and therefore to PrEP failure.

| Pat. | PrEP before HIV+ test (days) | PrEP Use | Age at HIV-Dx (Yrs) | Last HIV test before HIV+ (days) | Serology (western blot) Fiebig stage | Clinical Symptoms | HIV RNA (c/mL) | Resistance, Subtype |
|---|---|---|---|---|---|---|---|---|
| 1* MSM | 20 | Cont | 25 | -49 | II | None | 130 | n.a.*** |
| 2 MSM | 226 | OD** | 32 | -231 | V | Fever, sore throat, cough, night sweats | 3.000.000 | E138A CRF02_AG |
| 3 MSM | 595 | Cont | 31 | -146 | VI | Fever, sore throat | 17400 | M184V |

*possible risk contact within 3 weeks prior to PrEP start; **taken irregularly, only 3 pills/event; ***too low for resistance analysis; Cont=continuous, OD= on demand PrEP use

### 096
### Paper-ID: 47261, P6

### HIV post-exposure prophylaxis (PEP)—the perspective of the A&E department at a Tertiary Referral Centre with a specialized HIV centre

Stefan Schlabe[1], Christoph Boesecke[1], Carolynne Schwarze-Zander[1], Kathrin van Bremen[1], Ingo Gräff[2], Burger Christof[3], Jan-Christian Wasmuth[1], Jürgen K. Rockstroh[1]
*[1]Universitätsklinikum Bonn, Medizinische Klinik I; [2]Universitätsklinikum Bonn, Interdisziplinäres Notfallzentrum (INZ); [3]Universitätsklinikum Bonn, Orthopädie und Unfallchirurgie*

**Introduction:** Administration of antiretreviral therapy following exposure to HIV (HIV-PEP) has become a mainstay of risk reduction. While regularly in the hands of ID specialists, occupational medicine departments and STI clinics, studies showed that Emergency physicians can reliably prescribe PEP, but studies on PEP in Accident&Emergency departments (A&E) are still scarce.
**Methods:** We analysed retrospectively data of all identifiable patients presenting to the Internal Medicine Specialist with reported potential HIV exposure event in the A&E of the University Hospital of Bonn from 2012 to 2020. We assessed gender, age, exposure event, time, prescription of PEP, linkage to care and accordance with current guidelines and compared with 101 patients linked to our walk-in-clinic 2014–2017.
**Results:** 315 patients (71.7% male; median age 31y) presented to A&E after 345 potential exposure events (occupational 31.3% (108), non-occupational 66.7% (230)). Only 76 presentations (22%) occured at office time, 140 (40.6%) on weekends, median time after event was approximately 12 h (80.4% $<$ 24 h). Most exposure events were sexual contacts (198; 57.4%, 96 MSM, 97 heterosexual), needle injury (81; 23.5%) and contact to blood (44; 12.7%). 33 events (9.6%) were connected with sexual assault or violence (rape 12, bites 7, attacks 9, violent needle stitch 3, stealthing 2). 75 index persons (21.7%) were known HIV positive with only 10 below detection limit ($<$ 40 copies/mL). In 152 cases (44.1%) PEP was prescribed. Only 92 patients were linked to specialist care in our walk-in-clinic, 79 after prescribing PEP. 7.8% of indications of PEP withhold or administration were different from guideline recommendations.
**Conclusions:** Evaluation of HIV-PEP is a regular event in our A&E, especially at out-of-office time underlining their role. Compared to our walk-in-clinic, we saw higher frequency of non-occupational and heterosexual exposure events, violent cases and more often unclear status of the index person. A&E-Management and linkage to care is still challenging.

### 097
### Paper-ID: 47229, P7

### Measuring PrEP users' customer satisfaction with the pharmacy services of 'Marien Apotheke Wien'

Irene Polt[1], Sandra Dohr[1], Viola Reiner[1], Laura Gaugg[1], Frank Michael Amort[1], Karin Simonitsch[2], Jürgen Königshofer[2]
*[1]FH JOANNEUM Bad Gleichenberg, Institut Gesundheits- und Tourismusmanagement; [2]Marien Apotheke Mag. pharm. Karin Simonitsch e.U.*

**Introduction:** Pre-exposure prophylaxis (PrEP) is a medicamentous HIV prevention strategy, prescribed for people with substantial risk of HIV. Therapies with PrEP include quarterly screenings and health examinations. In Austria, the costs for PrEP are currently not covered by public health insurance. PrEP is available at 'Marien Apotheke Wien', which aims to measure customer satisfaction of PrEP users concerning their pharmacy services.
**Methods:** The method is comprised of four different parts: A structured database inquiry to illustrate the current state of research on PrEP, customer satisfaction and pharmacy services. Guided interviews with experts in the field of customer satisfaction will provide a basis for the development of further survey instruments. Customer satisfaction of PrEP users will be measured with standardized questionnaires (accessible via QR code) with 100–150 answers to be expected. Open qualitative interviews with three PrEP users serve to gain an insight into people's experiences using PrEP. Based on a descriptive analysis, the results will be processed and recommendations will be put forward.
**Results:** The project started in October 2020 and will be completed in June 2021. So far, research shows that PrEP has a high potential for HIV prevention as it is very effective and rarely causes severe side effects, however the role of pharmacies in service provision and customer satisfaction has not been researched in that area. The expert interviews show that customer satisfaction is a multidimensional and complex concept. When offering pharmacy services, attention needs to be paid to subtlety and discretion. The survey to be conducted in spring 2021 will be the first in German language measuring customer satisfaction and providing recommendations for improving pharmacy services for PrEP users.
**Conclusions:** This research project will be able to present for the first time an analysis of customer satisfaction with pharmacy services related to PrEP in Austria.

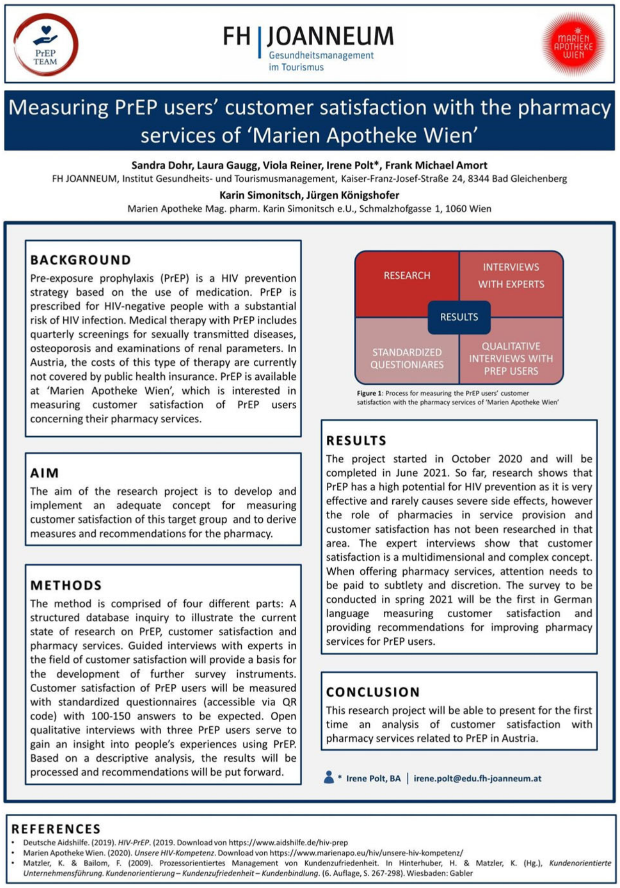

098
Paper-ID: 47284, P8

### No increased exposure to hepatitis E virus (HEV) in PrEP users

Guido Schäfer, Anja Hüfner, Till Koch, Hanna Matthews, Stefan Schmiedel, Sabine Jordan, Olaf Degen, Sven Pischke
*Universitätsklinikum Hamburg-Eppendorf*

**Introduction:** As for hepatitis A virus (HAV) it has already been shown that there is an increased risk of exposure in MSM, controversial data exist for hepatitis E virus (HEV). Some studies showed an increased anti-HEV seroprevalence in MSM, others could not confirm this. We investigated anti HEV seroprevalence in men receiving HIV-pre-exposure prophylaxis (PrEP).
**Patients and methods:** A total of 61 PrEP-using men (age 21–56 years, mean 34, standard deviation 8 years) and a control cohort of 1000 blood donors were studied. Testing was performed for anti HEV IgG and IgM (Wantai assay).
**Results:** 18.0% (n = 11) of PrEP users were IgG positive and 1 was IgM positive (1.6%). This was not significantly different from blood donors (166/1000 IgG positive [16.6%], 11/1000 IgM positive [1.5%]). The age of IgG-positive PrEP users did not differ from IgG-negative ones.
**Conclusion:** In contrast to HAV-transmission, sexual transmission does not appear to play a role for HEV-transmission.

099
Paper-ID: 49998, P9E

### Opportunities to improve HIV pre-exposure prophylaxis (PrEP) implementation in persons at risk of acquiring HIV—results of a survey among physicians in Germany

Mary Katherine Sammons[1], Matthew Gaskins[1], Frank Kutscha[1], Alexander Nast[1], Ricardo Niklas Werner[1]
*[1]Klinik für Dermatologie, Venerologie und Allergologie, Division of Evidence-Based Medicine (dEBM), Charité-Universitätsmedizin Berlin*

**Background:** Physicians in Germany are entitled to bill for PrEP-related services with the statutory health insurance if they are certified in accordance with the national HIV/AIDS quality assurance agreement ('HIV-specialists'). Non-HIV-specialists of certain specialties may acquire the approval to bill for PrEP-related services if they accomplish further training, including a 16-h internship in an HIV care facility. Because these facilities are rare, particularly in rural regions in Germany, the certification requirements repre-sent a substantial barrier, and could in consequence lead to gaps in the availability of PrEP services.
**Methods:** A random sample of 2,200 physicians (GPs, internists, dermatologists, urologists) and the members of the German STI and AIDS societies (DSTIG and DAIG) were invited to participate in our survey. We asked for the proportion of proactive advice on PrEP provided in appointments with MSM and trans persons who met the criteria to be of-fered PrEP according to the German-Austrian guideline ("at-risk patients"). Self-assessed knowledge and attitudes towards PrEP were assessed in form of summa-tive scores (0–20 points), calculated from individual items.
**Results:** 154 physicians participated [HIV-specialists: 47.7%, Non-HIV-specialists: 52.3%]. The proportion of proactive PrEP-advice was higher among HIV-specialists than among non-HIV-specialists [Mdn = 30.0% (IQR = 63.5) vs. Mdn = 0.0% (IQR = 11.3); $p < 0.001$]. Both the knowledge and the attitudes towards PrEP were higher/more positive among HIV-specialists than among Non-HIV-specialists [knowledge score: Mdn = 20.0 (IQR = 0.0) vs. Mdn = 4.0 (IQR = 11.0), $p < 0.001$; attitudes score: Mdn = 18.0 (IQR = 3.0) vs. Mdn = 13.0 (IQR = 5.25), $p < 0.001$]. In the multiple linear regression, however, only knowledge about PrEP, but not HIV-specialist status, was an independent predictor of the proportion of proactive PrEP-advice.
**Conclusions:** The findings point to opportunities for improving PrEP implementation in Germany, given that the variability of PrEP knowledge was high among Non-HIV-specialists. Targeted training and lower-threshold certification requirements to bill for PrEP-related services could help improve PrEP access, particularly in rural areas where HIV-specialists are rare.

### Prevention other than PrEP

100
Paper-ID: 47249, O1

### Are gender nonconforming people exposed to a higher risk of HIV compared to their gender conforming peers?—results of a retrospective study at Checkpoint BLN, Germany

Christoph Weber[1], D. Paulina Matyjas[1], Yvonne Delsemmé[1], Jacques Kohl[1], Jochen Drewes[2]
*[1]Checkpoint BLN; [2]Freie Universität Berlin*

**Introduction:** Gender nonconforming people (GNC) are key population in HIV prevention worldwide, with a constellation of social stressors and structural inequalities leading to increased risk of HIV infection. Between 10 and 15% of visitors of Checkpoint BLN define themselves as trans or non-binary. Yet little is known about this population in Germany. Therefore, we retrospectively examined the differences between gender nonconforming and their gender conforming peers at Checkpoint BLN.
**Methods:** Retrospective exploration of 2037 questionnaires and clinical data of visitors of Checkpoint BLN between 4/2019 and 04/2020 regarding socio-economic characteristics, substance consumption and sexual behavior. The gender nonconforming group (GNC = trans × M/F and non-binary persons) was compared with users who define themselves as gender conform (GC = cisM/F). We applied Pearson's chi-square for categorical data and the t-test for metric data.
**Results:** The mean age: 30.8 years (SD: 7.96; R: 16–75). GNC (n = 187) compared to GC (n = 1845) showed no differences regarding age, sexual orientation, origin, education and chemsex usage. The GNC differed significantly for precarious income (OR: 1.612 [95% CI 1.14–2.29]; p = 0.007), were more often engaged in sex work (OR: 4.415 [95% CI 2.68–7. 29]; $p < 0.001$), had health insurance less frequently in Germany (OR: 0.572 [95% CI 0.40–0.81]; p = 0.001) and used PrEP less frequently (OR: 0.367 [95% CI 0.16–0.84]; p = 0.014). GNC had more gender nonconforming sexual partners (OR: 13.347 [95% CI 9.58–18.59], $p < 0.001$) and more sexual partners in the previous 6 months (OR: 1.45 [95% CI 1.06–1.97], p = 0.02), with no differences in the frequency of condomless anal intercourse and STI/HIV diagnoses.
**Conclusion:** Gender nonconforming show significant differences to their conforming peers and need to be addressed separately in order to lower barriers to HIV-prevention. Their specific prevention needs are currently underserved in Germany. Further research should focus more on this growing population.

### 101
### Paper-ID: 47292, O2

### Still, virus is undetectable! Results on the sex and relationship of people living with HIV and the changes achieved through "Treatment as Prevention"

Franziska Hartung[1], Janine Dieckmann[1], Heike Gronski[2], Matthias Kuske[2], Marie-Theres Piening[1], Eléonore Willems[2]
*[1]Institut für Demokratie und Zivilgesellschaft; [2]Deutsche Aidshilfe e.V. (DAH)*

**Introduction:** People living with HIV (PLHIV) experience stigmatisation and discrimination (S&D), especially in relationships and their sex lives—a domain where people are extremely vulnerable and which is strongly linked to the own identity. Still, the importance of this life domain is often neglected in HIV stigma research. Therefore, the participatory research project "positive stimmen 2.0" of the Deutsche Aidshilfe and the Institute for Democracy and Civil Society particularly addresses this issue.
**Methods:** The results are based on an online survey with PLHIV (N = 935) and peer-to-peer Interviews, which where conducted during 2020 in Germany. In a mixed-method design, the data of the two quantitative surveys are supplemented with qualitative focus groups (survey period: November 2020–March 2021) in order to achieve a deeper understanding of the topic. One focus of the study examines the connection between HIV-related S&D, a satisfying sex life, and how "Treatment as Prevention" (TasP) affects this.
**Results:** 55 percent of the participants reported being sexually rejected at least once in the year prior to the study due to their HIV infection. 28 percent have experienced offensive sexual comments. We also found strong correlations between high internalized stigma and subjectively perceived impairments in sex life and relationships. Regarding knowledge of TasP, PLHIV reported strong empowering effects. Nevertheless, question remains whether effects of TasP are not just cognitively, but also influence emotions and actions of PLHIV. Although 96 percent reported to trust the protective effects of therapy, 32 percent are still sometimes afraid of infecting someone.
**Conclusion:** S&D can affect PLHIV's satisfaction with sex life and relationships. Therefore, in the context of a comprehensive understanding of sexual health, these life domains should be given greater attention in medical care, educational outreach and HIV counseling. Future research should focus on effects of stigmatisation of HIV by TasP.

### 102
### Paper-ID: 47048, P1

### Are we reaching the at-risk patient populations for HIV and other STI?

Christoph Boesecke[1], Torben Kimhofer[2], Christopher Tocha[3], Oliver Schubert[3], Rainer Rybak[3], Klaus Kuhlen[1], Christoph Klaes[3], Jürgen Rockstroh[1]
*[1]Universitätsklinikum Bonn; [2] Murdoch University; [3] AIDS-Hilfe Köln*

**Introduction:** Here we report annual results of STI screenings and survey data from a large community based STI testing Checkpoint in Cologne, Germany.
**Methods:** From January 2017 to December 2019, data on STI screening, clinical, demographic, sexual information was anonymously recorded for individuals attending the Checkpoint in Cologne. Visitors were screened for HIV, syphilis, chlamydia, gonorrhea, hepatitis C using point of care testing kits.
**Results:** Overall, screening was performed on 11,456 visitors aged 16–85 years for three main reasons: recent HIV risk situation (46%), routine testing (29%), beginning of a new relationship (24%). Largest visitor group constituted men who have sex with men (MSM, 44%), followed by men who have sex with women (MSW, 29%), women having sex with men (WSM, 22%). MSM engaged on average with a higher number of sex-partners than MSW and WSM with 36% having 2–5 sex-partners and 10% having $\geq$ 26 per year. The annual number of visitors on PrEP (96% MSM) increased steadily, with a total of 29 visitors in 2017, 54 in 2018 and 123 in 2019 ($p < 0.001$). MSM group had the highest disease frequency (chlamydia: 140, gonorrhea: 123, syphilis: 88, HIV: 56, HCV: 2). STI frequency in PrEP users was highest for chlamydia/gonorrhoea (7%, 9% and 12% for 2017, 2018 and 2019, respectively). Despite increased PrEP prevalence in Germany since 2017 (covered by health insurance from 09/2019 onwards) no decline in HIV infection rates was observed with 20, 29 and 24 cases in 2017, 2018 and 2019, respectively ($p = 0.46$). 56 new HIV infections were seen in MSM, 4 in MSW, 7 in WSM.
**Conclusions:** Checkpoint was able to detect relevant STIs in 5%. Despite increased HIV awareness and PrEP roll-out MSM remain at highest risk for contracting HIV highlighting the continuous need for low-threshold STI screening capacities.

### Chemsex, drug use

### 103
### Paper-ID: 46558, O1

### Chemsex and mental health of men who have sex with men in Germany

Annette Bohn
*LVR-Klinikum Essen*

**Introduction:** Chemsex is defined as using certain substances immediately before or during sexual activities to facilitate, prolong and/or intensify sexual experience, mainly by some communities of men who have sex with men (MSM). Four substances are typically associated with chemsex: methamphetamine, mephedrone, GHB/GBL, and ketamine. So far, chemsex behavior in Germany has not been studied on a larger scale. The study aims to describe general characteristics and aspects of mental health among a sample of German MSM who engage in chemsex and to describe potentially adverse consequences of chemsex behavior.
**Methods:** The study refers to a subset of participants from the German Chemsex Survey, an MSMcommunity recruited, self-completed online survey with a self-selected convenience sample. A group of participants who used methamphetamine, mephedrone, GHB/GBL, and/or ketamine in a sexual setting in the last 12 months (n = 280, chemsex group) was compared to a group of men who did not use substances in a sexual context (n = 177, non-chemsex group) in regard to mental health measures, sexual health, HIV infections and experiences of non-consensual sex acts.
**Results:** 41.2% of the chemsex group reported being HIV positive, 2.0% reported being infected with hepatitis C. Significantly more men from the chemsex group were HIV-positive than those from the non-chemsex group. The chemsex group showed significantly higher mean scores for depression, anxiety, and somatization than the non-chemsex group, but effect sizes were low. The chemsex group reported significantly higher incidences of non-consensual sex acts compared with the nonchemsex group.

**Conclusions:** The rate of 41.2% HIV positive in the chemsex group is higher than the rate of HIVpositive German MSM in 2010, which was 8.0%. Although heightened mean scores for mental health measures were found for the chemsex group, there was no difference in distribution of clinically relevant symptoms between the groups.

### 104
### Paper-ID: 46961, O2

### Substance use and sexuality: perspectives from addiction treatment patients and professionals

Daniel Deimel, Niels Graf
*Deutsches Institut für Sucht- und Präventionsforschung, KatHO NRW*

**Introduction:** Research demonstrates manifold associations between sexuality and substance use as, for example, a higher risk of developing substance use disorders due to sexual abuse or the disinhibitory effects of specific substances which may facilitate and intensify risky sexual encounters. These associations indicate that sexual activities represent a risk of relapse after completion of addiction treatment. Yet, there is hardly any research on substance use and sexual behaviors in patients with a substance use disorder. This presentation, therefore, examines if and how issues of sexuality gets are addressed in addiction treatment.
**Methods:** Expert interviews (n = 30) with professionals and a self-report questionnaire among patients (n = 480) from outpatient and inpatient addiction treatment facilities in Germany.
**Results:** Depending of the substance primarily used and the gender of the patients, patients link sexual thoughts and behaviors to drug use. Correspondingly, both professionals and patients view sexuality as a highly relevant issue. Nonetheless, drawing attention to sexuality within addiction treatment is not an institutionalised practice. Instead, a treatment of the issue depends of the motivation and knowledge of single professionals. Moreover, the issue is not treated with the help of acknowledged concepts (e. g. sexual anamnesis), but based on personal thoughts and experiences.
**Conclusions:** Despite its relevance, sexuality is rarely addressed in addiction treatment. Therefore, a systematic consideration of sexuality within addiction treatment sensitive to patients´ gender and sexual orientation is necessary. This requires awareness raising among addiction treatment professionals as well as training specifically focused on talking about sexuality.

### 105
### Paper-ID: 47251, P1

### Sexualized drug use among MSM taking PrEP—a cohort analysis

Guido Schäfer, Olaf Degen, Sabine Jordan, Stefan Schmiedel, Anja Hüfner, Till Koch
*Universitätsklinikum Hamburg-Eppendorf*

**Introduction:** Epidemiological data on sexualized drug use (chemsex), especially in Germany are scarce and there is a lack of knowledge of quality and frequency of the use of recreational drugs during sex among MSM. We present first outcomes of a cohort of MSM, presenting for HIV-PrEP care in our tertiary infectious diseases unit.
Methods: Starting January 2020, all of the PrEP users, presenting in our infectious diseases unit, were asked to fill a questionnaire about specific PrEP-related items like sexual risk behaviour and chemsex.
**Results:** Presentation of the first results of (as of 12/2020) 137 participants.

- Do you use alcohol and/or drugs during sex: yes, often: 9%, yes, occasionally 56%, no, never 35.1%
- Which drugs/substances are used? Alcohol 72 (82.8%), erectile dysfunction agents (e.g. Viagra®, Cialis®, Levitra®) 12 (13.8%), Poppers 50 (57.5%), GBL/GHB 3 (3.4%), MDMA 11 (12.6%), Cocaine 6 (6.9%), Amphetamine 10 (11.5%), Methamphetamine 1 (1.1%), Ketamine 2 (2.3%), Cannabis 22 (25.3%), Methadone 2 (2.3%), Mephedrone 5 (5.7%).
- In which settings and how are the drugs taken? Injected (into the vein) 2 (3.8%), injected (but not into a vein/vessel) (2, 3.8%), sniffed 22 (42.3%), inhaled 35 (67.3%), orally taken 25 (48.1%), used own utensils 16 (61.5%).
- If shared utensils: with partner in relationship 2 (20%), with a good friend/regular sex partner 8 (80%), with a casual sex partner 2 (20%).

**Conclusion:** More awareness on sexualized drug use is needed, especially as there is consistent data about increasing frequency of chemsex, especially among MSM. We contribute data on qualitative and quantitative use of recreational drugs and specific drug-related behaviour during chemsex.

### 106
### Paper-ID: 46945, P2

### ChemSex—guidance and knowledge transfer for chems-users, HCPs, and NGOs: three interlinked projects in Vienna

Thomas Baumgartner[1], Matthias Egger[2], Birgit Leichsenring[3]
[1]*Aids Hilfe Vienna;* [2]*Gilead Sciences;* [3]www.med-info.at

**Introduction:** Consumption of chemical substances in a sexual context (ChemSex) by men who have sex with men and trans*persons (Chems-Users) appears to be increasing in Austria. However, ChemSex is rarely addressed in its multifactorial entirety due to content- and target group-specific orientations of healthcare facilities. Therefore, Chems-Users and healthcare providers (HCPs/NGOs) lack suitable contact points or specialized colleagues, respectively. Raising awareness, education as well as guidance and networking within and between both groups (Chems-Users and HCPs/NGOs) are essential.
**Methods:** Initiated through "HIV and Your Body" public information events, a ChemSex Network for Viennese healthcare providers was founded. Collaborations within this network led to development of a novel Guidance Tool for Chems-Users and HCPs/NGOs. For both groups, knowledge transfer and networking will be strengthened by continuous public events.
**Results:** Established network meetings enable exchange between Viennese professionals which are or may be confronted with ChemSex. The wide range of participating healthcare providers reflects the complexity of the topic. Joint and mutual trainings offer cost-effective education for HCPs/NGOs from different disciplines. Network-contacts increasingly facilitate interdisciplinary collaborations, also regarding care of individuals. Additionally, Austria's first online ChemSex Guidance Tool emerged from active networking: Viennese healthcare providers professionally dealing with the topic can be found on a simply structured homepage. A first evaluation of the tool applying a quick survey is planned for early 2021. Experiences from public Information Events show that regarding ChemSex, formats not targeting a specific audience may be advantageous, since Chems-Users and HCPs/NGOs can equally benefit and establish unprejudiced contact to one another.

**Conclusions:** Networking as well as merging expertise and resources may seem trivial but require initial steps and sustained efforts. By interlinking three unique Austrian projects, the ChemSex Network, the ChemSex Guidance Tool, and public ChemSex Information Events, a resource-saving sustainable foundation for addressing ChemSex-related topics was created.

**107**
**Paper-ID: 46636, P3**

**Harm reduction in North-Rhine Westphalia: a recommendation for risk reduction and health promotion for people who use drugs**

Domenico Fiorenza[1], Friederike Carnehl[2]
*[1]Geschäftsstelle der AG AIDS-Prävention NRW; [2]Landesstelle Sucht NRW*

**Introduction:** Plenty of scientific evidence demonstrates the positive impact of harm reduction measures on the health and well-being of people who use drugs (PWUD). As a matter of health equity, these resources should be accessible for all PWUD. However, availability differs considerably within North-Rhine Westphalia (NRW); the prevalence of HIV, viral hepatitis and other drug-related harms remains a concern (Deimel et al., 2018). These remaining gaps in the access to harm reduction provoked the initiation of a working group to elaborate region-wide recommendations.
**Methods:** Two committees representing the federal state of NRW, municipalities and nongovernmental organizations ("AG AIDS-Prävention NRW" for the field of HIV prevention, "Beirat der Landesstelle Sucht NRW" for the field of drugs and addiction) delegated members to a common working group which met for three workshops in 2019. The formulation of the recommendation was based on a literature review and the equal, open discourse in the working group.
**Results:** The recommendation „Harm Reduction: Risiken mindern – Gesundheit fördern" was passed by both committees in August 2020. It encourages HIV and drug help services and authorities to open a discourse on services for PWUD, to use of all options at hand, and to prioritise health promotion.
**Conclusions:** There are three main conclusions on structural (1), procedural (2) and substantial (3) level:

1. Cooperation is key. Cooperation between different albeit overlapping help systems (HIV/AIDS and drugs/addiction) has proven to be highly instructive for the overall process.
2. Open dialogue is essential. It is worth the effort to initiate a discourse without proscriptions, to understand each other's mindset in order to reach for a common goal.
3. Harm reduction is, in theory, a well-established concept to improve the health and well-being of PWUD. The new recommendation sets a standard, encouraging a further diffusion of harm reduction in NRW and beyond.

**108**
**Paper-ID: 47705, P4**

**Linkage to care among people who inject drugs via harm reduction services in 6 German cities: facilitators and barriers**

Anna Dichtl[1], R. Zimmermann[2], N. Graf[3], D. Schaeffer[4], K. Dettmer[5], M. Gerlich[6]

*[1]Institute of Addiction Research, Frankfurt University of Applied Sciences, Frankfurt, Germany; [2]Department of Infectious Disease Epidemiology, Robert Koch Institute Berlin, Germany; [3]German Institute of Addiction and Prevention Research, Catholic University of Applied Sciences, Cologne, Germany; [4]Deutsche Aidshilfe, Berlin, Germany; [5]Independent consultant, on behalf of Federal Centre for Health Education, Cologne, Germany; [6]Federal Centre for Health Education, Cologne, Germany*

**Introduction:** HIV and hepatitis C (HCV) care cascades constructed from a multicentre sero-behavioural study in Germany identified insufficient testing and treatment rates for infected people who inject drugs (PWID). To identify barriers and evaluate effective measures, the 'HIV?, Hepatitis?, Das CHECK ich!' project (2017–2020) was set up, offering HIV/HCV counselling, point-of-care, confirmatory testing and linkage-to-care by staff in low-threshold harm reduction services (HRS) in 6 German cities between January 2018 and end of August 2019.
**Methods:** Qualitative evaluation addressed facilitators and barriers of testing, counseling, linkage-to-care by nine semi-structured face-to-face interviews with HCV positive-tested PWID (5 cities), a focus group with six HRS-staff (5 study-cities) and two semi-structured telephone interviews with treating medical doctors (MDs; 2 cities). Focus group and interviews were audio-recorded, transcripted, coded via MAXQDA and analysed.
**Results:** Important barriers for treatment initiation included insufficient or mis-information on treatment options and where to get treated, absence of a trusting and supporting person, language barriers during counselling and testing, imprisonment and absence of health insurance as well as absence of trained staff and regular funding in the HR services.
Effective linkage-to-care relied basically on an existing trusting relationship between PWID and HRS-staff. Besides providing information on treatment options, availability of translation during counselling and accompaniment to medical appointments played a significant role. Prompt treatment initiation after testing, treatment by HRS-doctors/MDs providing opioid-substitution treatment and secured treatment during imprisonment were other facilitators for treatment initiation, as well as MDs proactively initiating treatment and good cooperation between HRS and MDs.
**Conclusion:** HRS as a trusting environment proved well accepted and supportive as setting to initiate HIV/HCV counselling, testing and to support linkage to care in PWID. Barriers on system and provider level were identified and need to be addressed. For an expansion of these services in HRS, regular translation service for counselling, providing funding to increase trained HRS staff, and improving cooperation between HRS and MDs are recommended.

**109**
**Paper-ID: 46494, P5**

**We Care! A drug user interview project on screening and linkage-to-care (SLTC) in Hepatitis C (HCV)**

Willehad Rensmann[1], Dirk Schäffer[2], Elena Reuß[3], Monika Weischenberg[3], Michael Juckel[3], Jens Reimer[4], Stefan Christensen[5]

*[1]Aidshilfe dortmund e.v.; [2]Deutsche Aidshilfe e.V.; [3]Gilead Sciences GmbH; [4]Universität Hamburg; [5]Centrum für Interdisziplinäre Medizin, Münster; Universitätsklinikum Münster*

**Introduction:** Hepatitis-C-SLTC measures often fail to reach drug users in Germany. This is also observed in the Dortmund drug counselling facility (DCF) K!CK, which offers HCV-specific counselling/testing. Yet, knowledge about barriers and resources regarding testing, therapy initiation/implementation and health-related behavior is scarce. The “We Care!” project investigated these aspects and aimed to develop a lifeworld-oriented concept of action in addiction care.

**Methods:** 15 qualitative/guideline-based interviews with clients were conducted in DCFs in Dortmund and Cologne. An expert committee accompanied the study design, evaluation of results and development of recommendations.

**Results:** Factors influencing behavior towards HCV-related health are complex and vary inter-individually. Level of knowledge, psychosocial situation and consumption behavior are secondary: Thus, appropriate health behavior is also possible under difficult conditions. Subjectively perceived perspectives on life have a decisive impact, both encouraging and inhibiting. Exemplarily, clients lack confidence to be able to complete therapy. This is reinforced by them being called “untreatable”. Further, the benefits of therapy are questioned by the non-medical/medical care system due to an allegedly increased reinfection risk. HCV-infected persons often feel alone, partially excluded and generally overextended. Transparency regarding therapy frameworks is lacking. Professional support, especially at therapy initiation, plays a beneficial role. Inadequate health behavior is often associated with a denying/resigned reaction pattern.

**Conclusions:** A change of perspective from addiction assistance to holistic health promotion is required to support favorable health behavior of clients. Consulting situations must consider the complexity and diversity of the influencing factors. Firm cooperation of drug help and clinicians, the active raising of health issues and professional support can promote HCV-related health behavior. Measures should be community-based rather than individual-centered and follow a normalization strategy (i.e. involve peers and strengthen regular routine rather than risk testing). Motivation strategies focusing on subjectively perceived positive life perspectives and individual benefits should be promoted.

References:

Rensmann, Willehad (2020). “We care”: Qualitative Studie zum HCV-bezogenen Gesundheitsverhalten. Drogengebrauchender. Dortmund. Available via https://bit.ly/3iSpQTi

This project was conducted in cooperation with Gilead Sciences GmbH and financially supported by Gilead Sciences GmbH.

Project lead: Rensmann W., committee of experts: Reimer J., Christensen S., Schäffer D.

Disclosure of interests

J. Reimer: Speakers bureau: Camurus, Hexal, Indivior; Advisory: Camurus, Gilead, Sanofi-Aventis;

S. Christensen: Lectures and consulting services: Abbvie, Camurus, Gilead, Indivior, Janssen, MSD, ViiV;

D. Schäffer: Gilead, Abbvie; W. Rensmann: Gilead.

### Social aspects, stigmatization, barriers

### 110
### Paper-ID: 46741, O1

### The impact of socioeconomic deprivation on late HIV diagnoses: a cross-sectional study in Germany

Annemarie Pantke, Viviane Bremer, Barbara Gunsenheimer-Bartmeyer, Uwe Koppe, Matthias an der Heiden, Jens Hoebel, Niels Michalski, Norbert Bannert, Kirsten Hanke
*Robert Koch-Institut (RKI)*

**Introduction:** Late HIV diagnoses are associated with significantly poorer health outcomes and facilitate onward transmission. This study examined the effect of regional socioeconomic deprivation on late HIV presentation.

**Methods:** We used data from the InzSurv-HIV study between 2011 and 2018 containing information of the statutory notification of newly diagnosed HIV cases and the timing of HIV diagnosis determined by the BED-Capture-ELISA from dried blood spots. Data on regional socioeconomic deprivation was derived from the German Index of Socioeconomic Deprivation (GISD). The GISD data was merged to the InzSurv-HIV dataset on the basis of the 3-digit ZIP code. Outcome measures were a long-standing infection based on the ELISA result or an infection at the clinical stage of AIDS as diagnosed by a physician and reported via the statutory notification. The effect of socioeconomic deprivation on the timing of HIV diagnosis was analysed using multivariable logistic regression models.

**Results:** Overall, 66.2% (n = 6,185) of HIV patients had a long-standing infection and 13.1% (n = 1,536) had reached the clinical stage of AIDS at the time of diagnosis. The highest proportions of late diagnoses were found among people aged 50+ (71.2% long-standing and 23.9% AIDS) and those with heterosexual transmission (76.5% long-standing and 16.4% AIDS). Men who have sex with men (MSM) living in regions belonging to the highest deprivation quintile were more likely to have a longstanding infection (OR: 1.29, 95% CI 1.04–1.60) as well as more likely to have developed AIDS (OR: 1.34, 95% CI 1.03–1.74) compared to the medium and low (base) deprivation quintiles. No effect was observed in the heterosexual transmission group.

**Conclusions:** In order to decrease late HIV diagnoses in Germany, efforts in promoting HIV awareness and regular testing behaviours have to be increased especially for heterosexual persons regardless of socioeconomic background and MSM in regions of higher socioeconomic deprivation.

### 111
### Paper-ID: 46950, O2

### Anticipated and experienced stigma as predictors for contact with healthcare services among sex workers

Deborah Hacke
*Modellprojekt Roter Stöckelschuh, BesD e.V.*

Sex work related stigma both influences mental and physical health among sex workers and their approach to help seeking in the healthcare sector. The fear of rejection or bad treatment keeps sex workers from disclosing their work as an important information to healthcare professionals. Sex workers wish for healthcare services which meet their demands for a sensitive, confidential, and accepting treatment. The project Red Stiletto (dt: Roter Stöckelschuh) by the German Professional Association for Erotic and Sexual Service Providers (dt: Berufsverband für erotische und sexuelle Dienstleistungen e.V.) faces those needs by providing an online map for sex workers which indicates sex worker friendly places to go. The project is cooperating closely with active and former sex workers by surveying their needs and experience reports, and carrying the resulting knowledge to further training about sex workers as clients for healthcare professionals. The project and it’s training program pursue the goal of harm reduction by lowering access barriers to healthcare services for sex workers and supporting their legal and health equality in society. For making the project successful it will be essential to make it known to healthcare professionals working in fields that are frequented by sex workers because of their engagement in sex work or in fields in which being engaged in sex work can be an important information for the treatment. Such fields can range from general practitioners to gynecologists, to psychotherapists, to infectious disease specialists, and to specialized medical practices for HIV-positive clients. Practitioners should gain further knowledge about sex workers' health and needs in their facilities to have the ability to meet the demands of this highly vulnerable target group that often faces multiple discrimination in healthcare services.

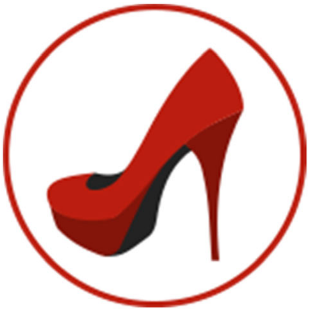

**112**
**Paper-ID: 47281, O3**

### Is queer a fashionable term or does it describe an actual difference? Explorative retrospective cross-sectional study within cis men at Checkpoint BLN, Berlin

D.Paulina Matyjas[1], Jochen Drewes[2], Yvonne Delsemmé[1], Jacques Kohl[1], Christoph Weber[1]
*[1]Checkpoint BLN; [2]Freie Universität Berlin*

**Introduction:** Queer is an emerging self-description among MSM. The term is not clearly defined and the notion that queer—often used to pronounce social and political differences—is a sexual orientation on its own is disputed among scholars. But what are the actual differences between queer (queCM), homosexual (hoCM), and bisexual cis men (biCM) regarding their risk factors for STI and HIV infection?
**Methods:** A total of 1.236 cis-male users of Checkpoint BLN (queCM: n = 103, 8.3%; hoCM: n = 859, 69.5%, biCM: n = 274, 22.2%) were assessed between 04/2019 and 04/2020 by questionnaire regarding socio-demographic characteristics, sexual behaviour and clinical data for STI/HIV were compared between groups. Non-parametric methods (Chi2 test, Kruskal–Wallis test) were used.
**Results:** The mean age was 29.4 (SD: 5.35). QueCM were more often born abroad (72.5% vs. 62.2% vs. 48.1%); $p < 0.001$), had less often German health insurance (71.7% vs. 79.8% vs. 83.9%; $p = 0.033$) and were more often unemployed (22.4% vs. 13.7% vs. 12.1%; $p = 0.035$). QueCM reported each higher numbers of sex partners (mean: 7.3 vs. 5.9 vs. 5.5; $p = 0.001$), of condomless intercourse (past 6 months) (2.1 vs. 1.5 vs. 2.1; $p < 0.001$), of trans and non-binary sex partners (35.0% vs. 4.1% vs. 18.6%; $p < 0.001$) and were more often involved in chemsex activities (42.2% vs. 29.3% vs. 25.6%; $p = 0.007$). PrEP was used equally often by queCM and hoCM and less often by biCM (11.7% vs. 14.9% vs. 3.7%; $p < 0.001$). There were no differences for gonorrhoea or chlamydia infections, as well as for syphilis and HIV infections.
**Conclusion:** There are specific differences in socio-demographic characteristics, as well as in sexual behaviour, substance use and choice of partner without differences for STI and HIV infections among queer cis men compared to their homosexual and bisexual peers.Differences of MSM subgroups need further investigations.

**113**
**Paper-ID: 47320, O4**

### Multiple stigmatisation and HIV: focus on vulnerable groups. Results on multiple stigmatisation and discrimination of people living with HIV

Marie-Theres Piening[1], Janine Dieckmann[1], Heike Gronski[2], Franziska Hartung[1], Matthias Kuske[2], Eléonore Willems[3]
*[1]Institut für Demokratie und Zivilgesellschaft; [2]Deutsche Aidshilfe e.V. (DAH); [3]Deutsche Aidshilfe e.V. (DAH)*

**Introduction:** HIV is still associated with the attribution as "other" and lifestyles and sexualities deviant from heteronormative ideas. People living with HIV (PLHIV) are not only discriminated and stigmatised because of their infection, but also because they belong to one or more stigmatised groups or are attributed with them. Therefore, the multidimensional experiences of PLHIV has to be considered in HIV research in particular. The research project "positive stimmen 2.0" focuses on that and takes concepts of intersectionality and syndemic into account.
**Methods:** The results are based on an online survey with PLHIV (N = 935) and peer-to-peer Interviews, which where conducted during 2020 in Germany. In a mixed-method design, the data of the two quantitative surveys are supplemented with qualitative focus groups (survey period: November 2020–March 2021) in order to achieve a deeper understanding of the topic. In addition to HIV-related stigmatisation and discrimination (S&D), overlapping and intertwining experiences of discrimination based on other attribution or group affiliations associated with HIV were also examined.
**Results:** Three quarters of the participants do not talk openly about their HIV infection. We found a negative correlation between stigma and openness. In addition, experiences of attributions and multiple discrimination play a role: A quarter of the participants agree that they have been attributed with having (allegedly) many sexual partners because of their HIV status. Furthermore, it was found that people who belong to further stigmatised groups, especially (Black) People of Color, show higher internalised HIV-related stigmatisation and lower life satisfaction.
**Conclusion:** Narratives about HIV are still related to overlapping and intertwining stigmas, which is embodied in the experience of S&D of PLHIV. This implies that anti-discrimination work must increasingly focus on multiple stigmatised and vulnerable groups as well as the reduction of social inequalities and stigma in general and in relation to HIV.

**114**
**Paper-ID: 46562, P1**

### Discrimination against a disabled person living with HIV (PLWHIV). An exceptional case without consequences for those who discriminate. A call for more awareness

Karin Cohrs[1], Kerstin Mörsch[2], Christin Engelbrecht[3], Christian Gailus[3], Matthias Stoll[4]
*[1]Hildesheimer AIDS-Hilfe e.V.; [2]Deutsche Aidshilfe e.V (DAH); [3]Aidshilfe Niedersachsen, Landesverband e.V. (AHN); [4]Medizinische Hochschule Hannover (MHH), Aidshilfe Niedersachsen, Landesverband e.V. (AHN)*

**Introduction:** Discrimination against people living with HIV remains a persistent problem in the health sector, although it is there that sufficient specific knowledge should be assumed.
**Methods:** Incident report.

**Results:** Mid-60s man, HIV-positive, antiretroviral well controlled, haemophiliac. He lives in a small, rural community. His HIV infection is not known in his social environment. A joint (knee)-surgery was performed for his haemophilic arthropathy. Postoperative he temporarily became a nursing case. The nursing home arranged a transport to a scheduled appointment with an infectiologist in another city. A large, state-approved aid organization was commissioned with this. The driver asks the nursing staff in the presence of the conscious and fully oriented patient about the clinical picture of his customer. He got the (completely dispensable) answer from the nurse that the patient is HIV-positive.
The driver then, contrary to duty, refuses the transport trip. The patient informed his wife by phone, while she was waiting for him with a wheelchair in front of the doctor's office. She called another emergency service who also refused the transport. The intended visit to the doctor could not take place. Multiple discrimination took place:
→ Humiliating conversation over the patient's head.
→ Breach of confidentiality.
→ Refusal to provide a service for no objective reason.
→ No effort on the part of the nursing home to support the patient in his project.
**Conclusions:** This case shows that the health care workers (ambulance, nursing staff) were not sufficiently trained in terms of confidentiality and hygiene regulations. Anti-discrimination work by the AIDS organizations is important, but not enough on its own. Making cases of blatant discrimination public, including among the supervisory authorities, and, if necessary, taking legal action against discrimination on the basis of the German General Equality Act (AGG), could lead to greater awareness of the problem.

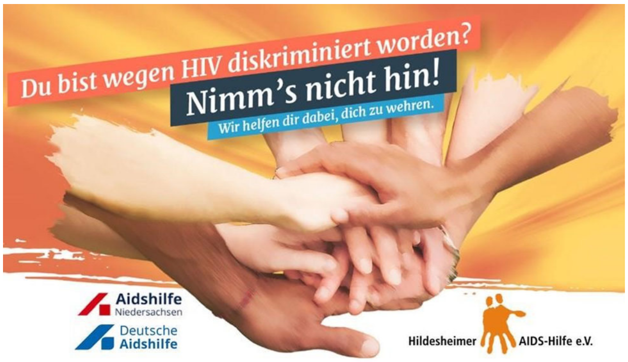

### 115
### Paper-ID: 46932, P2

### Two-way communication activities and sharing of treatment-related information between people living with HIV and their Healthcare providers

Alexandra Wigger[1], Bettina Berkowitsch[2], Ama Appiah[3], Rüdiger Allmann[1], Adam Stubbs[3], Patricia de los Rios[4], Chinyere Okoli[3]
[1]*ViiV Healthcare Munich Germany;* [2]*GSK Austria;* [3]*ViiV Brentford UK;* [4]*ViiV Quebec Canada*

**Introduction:** Patient-healthcare provider (HCP) communication is a "two-way street". We examined past sharing of health information between people living with HIV (PLHIV) and HCPs.
**Methods:** Data from 170 adult PLHIV from Austria (AT, 50) and Germany (DE, 120) in the Positive Perspectives 2 study were analyzed. We measured associations between being told by HCPs about U = U, and self-reported health outcomes, adjusting for gender, sexual orientation, age, and country.

**Results:** Mean age and HIV duration were 42.1 and 9.1 years, respectively; 60.0% [102/170] were men. The proportions reporting past, issue-specific discussions with their HCP were: among those concerned about long-term ART impact, 46.7% [42/90]; among those perceiving ART limits their life, 46.9% [23/49] (AT = 27.8% [5/18] vs. DE = 58.1% [18/31], p = 0.041); among those who ever wanted a different ART, 74.3% [52/70]. Overall, 74.7% [127/170] ever discussed U = U with their HCP, and these individuals had higher odds of optimal physical (AOR = 2.32, 95% CI 1.08–5.63), mental (AOR = 4.21, 95% CI 1.93–9.18), and overall health (AOR = 3.93, 95% CI 1.77–8.69); treatment satisfaction (AOR = 4.22, 95% CI 1.91–9.32), and sharing their HIV status with others besides their HCPs (AOR = 5.93, 95% CI 2.37–14.79). Overall, 70.0% felt sufficiently informed by their HCP to be involved in care, 69.4% were updated on new treatment options, 72.9% were asked their views before new treatments, 70.0% if they experienced side-effects, and 65.3% about any treatment concerns. Top perceived communication barriers among all participants were fear of being perceived as "difficult" (27.6%), difficulty bringing issue up (20.6%), and the perception nothing much could be done (18.8%) (Fig. 1). The percentage citing 'not enough time/opportunity' as a barrier was 24.0% [12/50] in AT vs 7.5% [9/120] in DE (p = 0.003).
**Conclusion:** PLHIV were less likely to discuss negative (e.g. undesirable medication effects) than positive issues (e.g. new treatments). U = U discussions with HCPs were associated with better health related outcomes. Addressing barriers that have a chilling effect on PLHIV's willingness to broach salient health issues may improve health outcomes.

| | Austria (N = 50) | Germany (N = 120) | Austria + Germany (N = 170) | All 25 surveyed countries (N = 2389) |
|---|---|---|---|---|
| **Perceived comfort discussing with HCP** | | | | |
| How my HIV medication affects other medications/drugs/pills I take * | 52.0 | 68.3 | 63.5 | 60.4 |
| Long-term side effects of my HIV medication * | 50.0 | 66.7 | 61.8 | 58.6 |
| The impact HIV is having on my life generally | 50.0 | 65.8 | 61.2 | 56.4 |
| Side effects of my HIV medication | 50.0 | 65.0 | 60.6 | 60.6 |
| Illnesses caused by HIV | 52.0 | 60.0 | 57.6 | 58.2 |
| Skipping/missing medication or forgetting to take my pill(s) each day * | 44.0 | 60.8 | 55.9 | 56.3 |
| The safety of others/preventing transmission | 48.0 | 54.2 | 52.4 | 59.8 |
| My emotional well-being | 50.0 | 50.0 | 50.0 | 53.5 |
| Privacy and not disclosing my HIV status | 48.0 | 41.7 | 43.5 | 52.0 |
| Having children | 34.0 | 47.5 | 43.5 | 44.9 |
| **HCP engagement** | | | | |
| HCP has told me about "undetectable = untransmittable" (U=U) | 84.0 | 70.8 | 74.7 | 66.5 |
| HCP seeks my views about treatment before prescribing an HIV medication | 76.0 | 71.7 | 72.9 | 62.8 |
| HCP asks me frequently about any side effects I might be experiencing | 80.0 | 65.8 | 70.0 | 63.0 |
| I am given enough information to be involved in making choices * | 82.0 | 65.0 | 70.0 | 62.6 |
| HCP tells me about new HIV treatment options that become available | 72.0 | 68.3 | 69.4 | 59.1 |
| I feel I understand enough about my HIV treatment * | 84.0 | 61.7 | 68.2 | 71.5 |
| HCP asks me if I have any concerns about my HIV medication | 72.0 | 62.5 | 65.3 | 65.1 |
| I would like to be more involved when it comes to treatment decisions | 54.0 | 50.8 | 51.8 | 65.1 |
| **Perceived communication barriers** | | | | |
| I don't want to come across as a 'difficult' patient | 36.0 | 24.2 | 27.6 | 26.7 |
| I'm not sure how to bring it up | 12.0 | 24.2 | 20.6 | 18.8 |
| I don't believe they can do much about my concerns | 16.0 | 20.0 | 18.8 | 21.3 |
| Don't think provider's priorities are the same as mine | 12.0 | 19.2 | 17.1 | 20.1 |
| I don't feel confident enough | 14.0 | 16.7 | 15.9 | 18.5 |
| I feel my main HIV care provider knows best | 20.0 | 11.7 | 14.1 | 22.6 |
| I don't want to take up more of their time | 22.0 | 10.8 | 14.1 | 16.0 |
| I don't feel it is important enough to bother them | 18.0 | 11.7 | 13.5 | 13.4 |
| Not enough time or the opportunity * | 24.0 | 7.5 | 12.4 | 19.3 |

### 116
### Paper-ID: 47294, P3

### Positive stimmen 2.0—a participative research project on stigmatization and discrimination of People Living with HIV in Germany

Matthias Kuske[1], Janine Dieckmann[2], Franziska Hartung[3], Heike Gronski[1], Marie-Theres Piening[3], Eléonore Willems[1]
[1]*Deutsche Aidshilfe e.V (DAH);* [2]*Institut für Demokratie und Zivilgesellschaft;* [3]*Institut für Demokratie und Zivilgesellschaft*

**Introduction:** 'Positive stimmen 2.0' is a follow-up study of the first implementation of the 'People Living with HIV (PLHIV) Stigma Index' in Germany 2012. The stigma index is a standardized instrument that gains empirical evidence on the impact of stigmatization and discrimination (S&D) on PLHIV. Beyond that, the current project focusses on additional research questions with a second study. Based on the findings, recommendations of action are developed for organizations, policy and other stakeholders. A main characteristic of the project is its participatory approach.
**Methods:** Core element of the project is the implementation of the second PLHIV Stigma Index in Germany (study I). 33 PLHIV were trained as peer-to-peer-interviewers. They conducted around 400 interviews in 2020 using the international standardized questionnaire. Study II extends the examination of outcomes of S&D on psychological wellbeing, sexual behavior, and the impact of multidimensional discrimination. Using a mixed methods design, it combines quantitative data (N = 935 PLHIV) with qualitative results of six focus groups. Participation of PLHIV is realized throughout the research process by peer-to-peer-interviewers and a community-based advisory board.

**Results:** First results of study II show that many PLHIV live well thanks to medical therapy. However, a large proportion is still confronted with HIV-related S&D in their lives. 90% answered that they 'live well with their HIV infection'. However, 52% of all participants stated that they are affected by prejudices about HIV. 56% stated that they experienced at least one discriminatory incident in the health care system during the last year, such as refusal of a service or obvious marking of the patient file.

**Conclusions:** By its participatory character, 'positive stimmen 2.0′ not only examines important empirical results, but also increases empowerment and engagement of PLHIV. All results will be published in 2021 and provide community-based recommendations to fight HIV-related S&D.

**117**
**Paper-ID: 47308, P4**

### Netzwerk Plus Frankfurt

Nils von Hentig[1], Ute Engelbach[2], Annette Piecha[3], Simon Merz[4], Eva Futterer[4], Carsten Gehrig[4]
*[1]BAG Darab-von Hentig; [2]Klinikum der Goethe Universität Frankfurt; [3]HIV contact; [4]AIDS-Hilfe Frankfurt*
Netzwerk Plus

Up to date, stigmatization and discrimination of people living with HIV/AIDS (PLWH) in the German health service is common. About 70% of PLWH have made this experience during the past 12 months of their treatment history, especially in non-HIV treating professions, such as general practitioners, dentists, surgeons, gynecologists and others. This leads to a high mental burden when attending other doctors than their HIV-practitioner and also to avoiding necessary medical treatments due to the fear of experiencing objection.

Netzwerk Plus was created to address this problem to medical specialists, who do not have much experience in treating PLWH. Netzwerk plus offers an online-based, low-threshold, modular structured, advanced training program for the intercourse with PLWH and provides more relevant information about the infection itself. Besides conveying basic information, e.g. ways of infection, hygienic standards, drug-drug interactions, it is a very special concern of Netzwerk Plus to also create more awareness about the psychiatric and psychosomatic disorders, which can occur in the context of HIV and AIDS, and which are oftenly unrecognized and thus treated very late. Participants can acquire a label and register their practice in a positive list, which is hosted by the AIDS-Hilfe Frankfurt (AHF) to guide patients to specialists welcoming PLWHA. Each module lasts approximately 45 min online and includes a final test presenting 10 questions. It is certified by the Medical Chamber of the State of Hessen. The modules are structured as follows: #1: Basics; #2: Subject-specific education; #3: Awareness raising, also for medical staff; #4: HIV and psyche.

Netzwerk Plus was founded by a group of specialists in internal medicine, pharmacologists, psychiatrists, and members of the medical authorities, community and AHF in the year 2018.

After having finished its test run, Netzwerk Plus will be online in January 2021.

**Open Access** This article is licensed under a Creative Commons Attribution 4.0 International License, which permits use, sharing, adaptation, distribution and reproduction in any medium or format, as long as you give appropriate credit to the original author(s) and the source, provide a link to the Creative Commons licence, and indicate if changes were made. The images or other third party material in this article are included in the article's Creative Commons licence, unless indicated otherwise in a credit line to the material. If material is not included in the article's Creative Commons licence and your intended use is not permitted by statutory regulation or exceeds the permitted use, you will need to obtain permission directly from the copyright holder. To view a copy of this licence, visit http://creativecommons.org/licenses/by/4.0/.

### Author Index

**A**
Aboud M 019, 020
Acosta R 031
Adam A 037
Adler K 082
Ahaus P 092, 093
Ait-Khaled M 020, 023
Ajana F 020
Akinwunmi B 011
Al Tayy A 047
Albanese M 002, 005
Albayrak-Rena S 040
Aldabbagh S 069
Allmann R 018, 115
Amort FM 097
an der Heiden M 012, 013, 110
Andreatta K 031
Angelis K 021
Ankert J 053
Antinori A 019
Appiah A 018, 115
Arastéh K 044
Arendt G 046, 048, 049
Arribas J 019

**B**
Baier M 082
Baker M 008
Balogh A 041
Bannert N 004, 110
Bartmeyer B 089
Bauer W 078
Baum A 010
Baumann R 041, 058
Baumann U 083, 084
Baumgarten A 041
Baumgartner T 106
Begley R 007
Behnke S 050
Behrens G 038
Belonosova E 009
Benn P 010
Bergmann S 057
Berhe M 021
Berkowitsch B 018, 115
Bermejo-Jambrina M 003
Bernhardt K 019
Berretta M 045
Beutel G 070
Bickel M 029, 045, 053, 058
Bilbilis K 043
Bischoff J 036
Bisshop F 020
Blair C 025
Bode M 011
Boesecke C 035, 036, 047, 051, 067 069, 085, 096, 102
Boffito M 037
Bogner JR 006, 015
Böhm M 014
Bohn A 103
Borgmann S 070
Bota M 070
Bower M 045
Brainard DM 025, 091
Braun P 017, 029, 030
Braun M 083, 084
Breitschwerdt S 047
Bremer V 089, 110
Brito M 045
Brockmeyer NH 040, 043, 077, 093
Bruening J 024
Brunetta J 028
Brunner C 054
Brust J 029, 030
Buchenberger D 011
Buchholz B 083, 084
Buggisch P 042
Buhk T 037, 095

**C**
Cahn P 019
Calvo M 039
Carnehl F 107
Carter CC 025, 091
Chang S 031
Chen H-R 002
Chichon P 064
Chiu A 007
Chounta V 010, 011
Christensen S 109
Christian S 035
Christof B 096
Chromy D 078
Chung A 001
Clarke A 019
Cohrs K 114
Collins SE 025
Collins S 031
Cordes C 024, 035
Cornejo AT 026
Corpus J 007
Crauwels H 008, 009, 010
Cravat L 085
Cristodero L 067
Curbelo R 028
Cutrell A 008, 009

**D**
D'Amico R 009
D'Antoni ML 031
Dalessandro M 021
Das M 091
Daugaard G 045
Däumer M 029, 030
de los Rios P 018, 115
de Ruiter A 022
de Wet J 026
De Wit S 020
de With K 070
Degen O 052, 053, 098, 105
Deimel D 104
Dekker A 075
Delsemmé Y 100, 112
DeMoor R 009
Dettmer K 108
Dewasurendra D 054, 055, 056
Dichtl A 108
Dickhoff M 048
Dickopf L 086
Dieckmann K-P 045
Dieckmann J 101, 113, 116
Dieing A 045
Dimmers F 046
Dohr S 097
Dolff S 040
Doll M 070
Dorey D 008
Draenert R 006
Drewes J 100, 112
Dumke R 079
Duvivier C 028
Dvory-Sobol H 007
Dymek KM 021

**E**
Eberwein L 070
Ebrahimi R 091
Eger J 006, 058
Egger M 106
Eis-Hübinger AM 069
El Kenz B 057
Elena R 070
Elliot E 037
Emmerich C 027
Enders K 073, 081
Engelbach U 117
Engelbrecht C 068, 088, 114
Engels R 005
Eron JJ 025
Esser S 012, 013, 029, 030, 040, 043, 080
Estrada V 023

**F**
Falk L 005
Feiterna-Sperling C 083, 084
Feldt T 033
Fellner E 074
Fenske S 037
Fetscher H 023
Finkenzeller T 009
Finkielsztejn L 011
Fiorenza D 107
Fischer C 004
Ford S 008
Ford SL 009, 010
Fousse M 050
Friebe M 060, 089
Friedrichs A 070
Fuchs A 013, 033
Funke B 024
Fürst J 070
Futterer E 117

**G**
Gailus C 068, 114
Galit A 001
Gallant J 031
Gapp M 002
Gartland M 019
Gaskins M 099
Gaugg L 097
Gehrig C 117
Geijtenbeek T 003
Geldmacher C 006
Gerlich M 108
German P 007, 025
Gersbacher E 006
Geusau A 078
Gillor D 017, 058
Gios L 071
Girard P-M 019
Gisinger M 054, 055, 056
Glück T 070
Goffinet C 004
Göllner K 088
Gottwald FE 015
Goulder P 001
Grabmeier-Pfistershammer K 078
Graf N 104, 108
Gräff I 096
Graham H 031
Greiffendorf I 070
Griffith S 009
Gronski H 101, 113, 116
Grove R 023
Gruenberger K 064
Grunwald S 058
Gulminetti R 023
Gunsenheimer-Bartmeyer B 012, 013, 110
Gute P 030

**H**
Haberl A 053
Hacke D 111
Hagins D 023
Hanhoff N 041, 060
Hanke K 110
Hansen S 037
Hanses F 070
Harrer EG 057
Harrer T 057
Harrington CM 010
Härter G 058, 059
Hartung F 101, 113, 116
Haubrich R 026, 027
Häussinger D 033
Heger E 014, 033
Heiken H 035
Heinzelbecker J 045
Heinzkill M 026
Heit M 050
Held K 006
Heldwein S 016, 029, 030
Hellwig K 070
Hentrich M 045
Herberhold C 088
Hillenbrand H 027
Hindman J 091
Hocqueloux L 026
Hoebel J 110
Hoffmann C 029, 030, 037, 041, 045 058, 059, 063
Hoffmann D 066
Hoffmann J 068
Hofmann K 002
Höller H 016
Holzki C 068
Hosmann K 044
Hove K 010
Hower M 040, 070
Huang JO 010
Hüfner A 052, 098, 105
Hung C-C 019
Hurissa Z 033
Hutz F 074

**I**
Ibarra Fonseca GJ 015
Ivanova A 041

**J**
Jäger H 016, 041
Jakob CEM 070
Jakobs J 038
Jansen J 004
Jarso G 033
Jeffrey J 008
Jensen B-EO 013, 014, 033, 035
Jessen H 027, 035
Jilma B 061
Jöckel K-H 040, 043
Jonsson-Oldenbüttel C 016
Jordan S 052, 098, 105
Juckel M 109
Jung N 070
Jurinovic V 045

**K**
Kaddu-Mulindwa D 050, 065
Kaiser R 014, 033
Kaiser F 040
Kann C 070
Karolyi M 061
Kasper A 077
Katner H 009
Kayser A 077, 092, 093
Kazmierski J 004
Keppler O 002, 005
Kerr C 091
Keuser L 065
Khaykin P 035
Kiebacher E 088
Kim C 026
Kimhofer T 102
Kitchen M 054, 055, 056
Klaes C 102
Klausen G 058
Klinker H 070
Knechten H 017, 027, 028, 029, 030, 058
Knickmann M 017
Knops E 014, 033
Kobbe R 083, 084
Koch T 098, 105
Koegl C 024
Kohl J 100, 112
Kollan C 012, 013, 034, 083, 084, 089
Königshofer J 097
Koppe U 012, 013, 090, 110
Köthemann W 017
Kreuter A 043
Krings A 076
Krznaric I 029, 030, 035, 045, 058 091
Kuhlen K 102
Kuhlendahl F 042
Kümmerle T 035
Kuritzkes DR 008
Kuske M 101, 113, 116, 083, 084
Kutscha F 099
Kuzmicic N 049

**L**
Landthaler M 004
Lange I 082
Lange U 087
Lapuente D 057
Lass-Flörl C 062
Lax H 043
Lee M 066
Lehmann C 053
Leichsenring B 106
Lengen S 017
Leone PA 021
Lesmeister F 045
Ling J 007
Lohr B 065
Lohse S 065
Lorenzen T 042
Lübke N 014, 033
Luedde T 033
Lutz J 007, 065
Lutz T 010

**M**
Mack F 027, 028
MacKay A 037
Madero JS 019
Maier C 057
Maischack F 040
Man C 019, 023
Marcus U 071, 090
Margolis DA 008, 009, 010
Marongiu A 028
Martin H 025, 031
Martin R 031
Marx B 069
Matthews JE 021
Matthews H 098
Matyjas DP 100, 112
Mavarani L 040
Mayer F 078
McKellar M 025
McNicholl I 031

Meixenberger K 004
Merbach M 060, 089
Merle U 070
Merrill D 021
Merz WM 085
Merz S 117
Meurer A 058
Michalski N 110
Migaud P 044
Mijočević H 066
Milovanovic M 070
Mirandola M 071
Monin M 035, 069
Morell EB 009
Mörsch K 114
Mosthaf F 041
Mounzer K 091
Mueller MC 058
Muigg A 054
Müller M 029, 030, 041, 044, 058
Mumm I 068, 088
Münchhoff M 001, 002
Mutevelli J 047

**N**
Nachbaur G 011
Nadalin S 070
Nambiar S 077, 092, 093
Nascimento M-C 020
Nast A 099
Necchi A 045
Neuwirth A 017
Nganou-Makamdop K 057
Nguyen C 021
Noe S 016, 038, 041

**O**
Oette M 043
Okoli C 018, 115
Omid S 061
Orhan E 046, 048, 049
Orkin C 009
Orth HM 033
Ortiz R 019, 021
Osiyemi O 020, 025
Otremba B 045
Owen A 037

**P**
Panstruga P 017
Pantke A 110
Pappa K 019, 020
Parks D 022
Paruszewski A 068
Patel P 008, 009
Pauli R 024, 027
Pawelka E 061
Peetz H 070
Perno CF 008
Petersen J 042
Pfister D 045
Philibert P 091
Piecha A 117
Piening M-T 101, 113, 116
Pilgram L 070
Pischke S 098
Plettenberg P 042
Plettenberg A 042
Pletz M 053
Polli JW 008
Polt I 097
Porteiro N 010
Porter DP 031
Portilla J 020
Pörtner K 012, 013
Posch W 003, 062
Postel N 006, 024, 027, 041, 058
Postmus D 004
Potthoff A 040, 043, 077, 093
Protzer U 066
Punekar Y 022

**Q**
Quercia R 008
Quirishi N 017

**R**
Raichle C 070
Ramgopal M 021, 025
Ramroth H 026
Rathert M 088
Rauschning D 070
Reiberger T 078
Reimer J 109
Reiner V 097
Reinsch N 040
Rensmann W 109
Reuß E 109
Rey PM 045
Rhee M 007
Riedel C 070
Rieg S 070
Rieger A 056, 078
Rieger A
Rieke A 025, 027, 058, 065
Rieke A
Rieke GJ 051, 069
Rissland J 065
Rittweger M 044
Rizzini P 011
Rockstroh JK 013, 019, 024, 031, 036, 045, 047, 051, 067, 069, 085, 096, 102
Rogers L 006
Roider J 001, 006, 058
Rolle C-P 021
Römer K 017, 038
Röseler S 070
Rößler S 058
Rothfuss K 070
Roustand L 011
Routy J-P 020
Rubach M 020
Ruck I 058
Rüger S 006
Ruppert N 011
Rüthrich M 070
Rybak R 102

**S**
Sabranski M 032, 037, 095
Sahali S 026
Salazar L 091
Salim A 051
Sammet S 080
Sammons MK 099
Sarcletti M 054, 055, 056
Sauer S 004
Schabaz F 016, 058
Schadendorf D 040, 043, 080
Schaeffer D 108
Schäfer G 013, 052, 098, 105
Schäfer H 070
Schäffer D 109
Schapiro JM 008
Schecke H 094
Scherzer J 008, 011
Schewe CK 037, 041, 058, 060, 095
Schink SB 071, 090
Schlabe S 069, 096
Schleenvoigt B 053, 082
Schmid M 070
Schmidt D 012, 013, 034, 060, 089, 090
Schmidt B 040
Schmidt K 057
Schmidt AJ 072
Schmiedel S 052, 098, 105
Schmuth M 055
Schneider J 035, 066
Scholten S 017, 035
Schöning S 083, 084
Schons M 070
Schörgenhofer C 061
Schreiber S 027
Schröder C 022
Schu M 073, 081
Schubert J 070
Schubert O 102
Schultheis B 070
Schulz C 053
Schulze-Sturm U 083, 084
Schürmann D 012, 013
Schwarze S 038
Schwarze-Zander C 036, 047, 051, 067, 069 085, 096
Schwerdtfeger C 066
Seitz T 061
Sewald X 005
Seybold U 015, 053
Shalit P 091
Shao Y 091
Sierra Madero J 023
Sievers J 019, 023
Simakoloyi E 087
Simonitsch K 097
Singh Y 009
Singh T 021
Skaletz-Rorowski A 077, 092, 093
Skoll M 078
Slim J 025

Smith KY 009, 010
Smith K 019, 020
Sohn M 004
Spinner CD 026, 035, 038, 058, 066, 070
Spornraft-Ragaller P 035, 079
Spreen WR 008, 009, 010
Spriewald B 057
Sprinzl M 070
St Clair M 008, 009
Stecher M 070
Steffen G 076
Stein L 058
Steiner S 070
Stellbrink H-J 007, 009, 012, 027, 032, 035, 037, 039, 095
Stephan C 024, 028, 053
Stimpfl T 061
Stirner R 006
Stocker H 044, 053
Stoehr A 029, 030, 042, 053
Stöhr A 026, 045
Stoll M 012, 013, 034, 068, 088, 114
Strassl R 078
Streeck H 038, 069
Strizek B 085
Stubbs A 018, 115
Swindells S 010

**T**
Talarico CL 008, 010
Tan DHS 009
Tenbusch M 057
Thams C 038
Thiagarajah S 009
Thorpe D 026, 028
Timm J 014
Tocha C 102
Torres AT 028
Tossonian H 028
Touzeau-Roemer V 078
Traugott M 061
Trebicka J 036
Trottier B 028
Tsai H-C 023
Tscharntke L 070
Tufa TB 033

**U**
Überla K 057
Uhrmacher M 093
Umland T 039
Ummard-Berger K 058
Underwood M 019
Underwood MR 021
Urbaityte R 019, 023

**V**
van Bremen K 036, 051, 067, 069, 085, 096
Van de Velde N 011
van Eygen V 008
van Lunzen J 008
Van Solingen-Ristea R 008, 009, 010
van Welzen B 026, 028
van Wyk J 019, 020, 021, 022, 023
Van Zyl L 010
Vandermeulen K 008, 009, 010
Vanveggel S 008, 010
Vecchio F 011
Vehreschild M 070
Vehreschild JJ 070
Vehring J 080
Vitcetz SN 004
von Bergwelt-Baildon M 070
von Heinegg EH 080
von Hentig N 117
von Krosigk A 016
Vosper J 062

**W**
Wach J 092
Wagenpfeil G 050
Waizmann M 028
Wang Y 008
Wang R 020
Waschulzik B 066
Wasmuth J-C 012, 013, 029, 030, 036, 047, 051, 067, 069, 085, 096
Weber C 100, 112
Weidlich S 066
Weischenberg M 109
Wenisch C 061
Werdecker V 065
Werner CA 068
Werner RN 099
Wesselmann H 053
West SK 007
White CT 008
White KL 031
Wichers C 068
Wiehler H 043
Wieland U 043
Wiese C 016
Wiesmann F 017
Wigger A 018, 038, 115
Wilflingseder D 003, 062
Wilkin A 025
Wille K 070
Willems E 101, 113, 116
Willinger B 078
Willkom M 031
Witte V 024
Wolf E 016, 029, 030, 035, 041, 063
Wong A 026
Wong P 091
Worm M 070
Wright J 020
Wu S 008
Wurapa A 021
Wyen C 020, 024, 029, 030, 058
Wyler E 004
Wynne BR 019, 020, 021, 023

**Y**
Yavuz A 043

**Z**
Zaderer V 062
Zahn A 039
Zeggagh J 026
Zimmermann R 076, 108
Zolopa A 023
Zoufaly A 059, 061, 064
Zutavern-Bechtold K 058
